# Polygenic risk scores predict diabetes complications and their response to intensive blood pressure and glucose control

**DOI:** 10.1007/s00125-021-05491-7

**Published:** 2021-07-06

**Authors:** Johanne Tremblay, Mounsif Haloui, Redha Attaoua, Ramzan Tahir, Camil Hishmih, François Harvey, François-Christophe Marois-Blanchet, Carole Long, Paul Simon, Lara Santucci, Candan Hizel, John Chalmers, Michel Marre, Stephen Harrap, Renata Cífková, Alena Krajčoviechová, David R. Matthews, Bryan Williams, Neil Poulter, Sophia Zoungas, Stephen Colagiuri, Giuseppe Mancia, Diederick E. Grobbee, Anthony Rodgers, Liusheng Liu, Mawussé Agbessi, Vanessa Bruat, Marie-Julie Favé, Michelle P. Harwood, Philip Awadalla, Mark Woodward, Julie G. Hussin, Pavel Hamet

**Affiliations:** 1 Department of Medicine, University of Montréal, CRCHUM, Québec, Canada; 2 The George Institute for Global Health, University of New South Wales, Sydney, NSW Australia; 3 Clinique Ambroise Paré, Neuilly-sur-Seine, and Centre de Recherches des Cordeliers, Paris, France; 4 Department of Physiology, University of Melbourne, Melbourne, VIC Australia; 5 Center for Cardiovascular Prevention, First Faculty of Medicine, Charles University in Prague and Thomayer Hospital, Prague, Czech Republic; 6 Oxford Centre for Diabetes, Endocrinology and Metabolism, University of Oxford, Oxford, UK; 7 University College London, Institute of Cardiovascular Science, London, UK; 8 School of Public Health, Faculty of Medicine, Imperial College London, London, UK; 9 School of Public Health and Preventive Medicine, Monash University, Melbourne, VIC Australia; 10 Boden Institute, University of Sydney, Sydney, NSW Australia; 11 Istituto Auxologico Italiano, University of Milano, Bicocca, Italy; 12 Julius Centre for Health Sciences and Primary Care, University Medical Centre Utrecht, Utrecht, the Netherlands; 13 Beijing Hypertension League Institute, Beijing, China; 14 Ontario Institute for Cancer Research, Toronto, ON Canada; 15 Department of Molecular Genetics and Dalla Lana School of Public Health, University of Toronto, Toronto, ON Canada; 16 The George Institute for Global Health, School of Public Health, Imperial College London, London, UK; 17 Montreal Heart Institute, Research Center, Montréal, Québec Canada; 18 Department of Medicine, Faculty of Medicine, Université de Montréal, Montréal, Québec Canada

**Keywords:** ADVANCE trial, Cardiovascular complications, Genetics, Polygenic risk score, Prediction, Renal complications, UK Biobank

## Abstract

**Aims/hypothesis:**

Type 2 diabetes increases the risk of cardiovascular and renal complications, but early risk prediction could lead to timely intervention and better outcomes. Genetic information can be used to enable early detection of risk.

**Methods:**

We developed a multi-polygenic risk score (multiPRS) that combines ten weighted PRSs (10 wPRS) composed of 598 SNPs associated with main risk factors and outcomes of type 2 diabetes, derived from summary statistics data of genome-wide association studies. The 10 wPRS, first principal component of ethnicity, sex, age at onset and diabetes duration were included into one logistic regression model to predict micro- and macrovascular outcomes in 4098 participants in the ADVANCE study and 17,604 individuals with type 2 diabetes in the UK Biobank study.

**Results:**

The model showed a similar predictive performance for cardiovascular and renal complications in different cohorts. It identified the top 30% of ADVANCE participants with a mean of 3.1-fold increased risk of major micro- and macrovascular events (*p* = 6.3 × 10^−21^ and *p* = 9.6 × 10^−31^, respectively) and a 4.4-fold (*p* = 6.8 × 10^−33^) higher risk of cardiovascular death. While in ADVANCE overall, combined intensive blood pressure and glucose control decreased cardiovascular death by 24%, the model identified a high-risk group in whom it decreased the mortality rate by 47%, and a low-risk group in whom it had no discernible effect. High-risk individuals had the greatest absolute risk reduction with a number needed to treat of 12 to prevent one cardiovascular death over 5 years.

**Conclusions/interpretation:**

This novel multiPRS model stratified individuals with type 2 diabetes according to risk of complications and helped to target earlier those who would receive greater benefit from intensive therapy.

**Graphical abstract:**

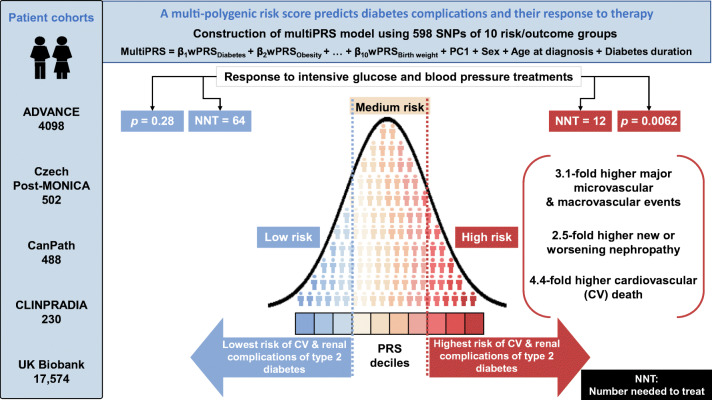

**Supplementary Information:**

The online version contains peer-reviewed but unedited supplementary material available at 10.1007/s00125-021-05491-7.

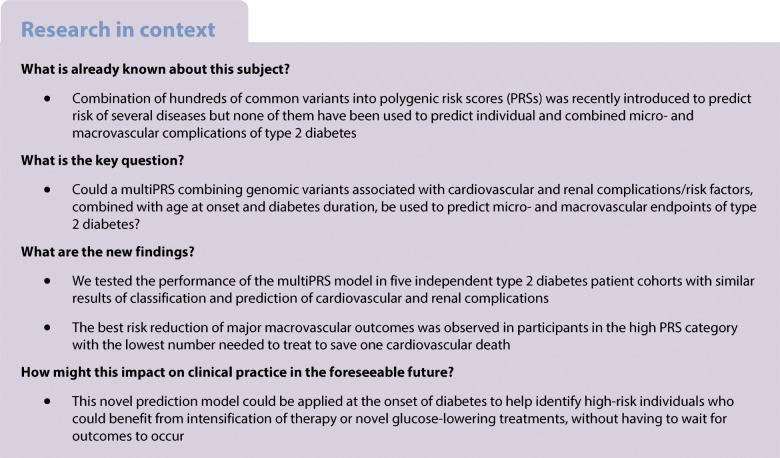



## Introduction

Diabetes increases the risk of serious and costly cardiovascular and renal complications [1, 2]. Prediction of risk prior to development of these complications is crucial to enable the targeting of individuals who could benefit from early intervention [3]. Genetic information, with which one is born, can be used to enable early detection of risk. Genome-wide association studies (GWAS) identified multiple common variants associated with type 2 diabetes [4–6], renal [7, 8] and cardiovascular diseases [9], and hypertension [10]. Individually, these genetic variants account for only a small effect size but the combination of hundreds of them into polygenic risk scores (PRSs) or genome-wide polygenic scores was recently introduced to predict risk of diseases [11–14] including type 2 diabetes [15]. Recent studies suggest that combining several PRSs of related traits into a joint model could optimise prediction [16–18]. Our aim was to develop a multi-polygenic risk score (multiPRS) prediction model that did not include any past risk/outcome data. Because of their common risk factors, overlap of its pathogenic mechanisms and correlations among them, a multiPRS composed of ten weighted PRSs (10 wPRS), gathering genomic variants associated with cardiovascular and renal complications and their key risk factors, was combined with sex and first principal component (PC1) of ethnicity, age at onset and diabetes duration into one logistic regression model, to classify or to predict micro- and macrovascular endpoints of type 2 diabetes [19–23]. The performance of the model was assessed by C-statistics in individuals of European descent with type 2 diabetes in the ADVANCE trial [24, 25] extended to its post-trial follow-up, ADVANCE-ON [26], for a total of nearly 10 years of observation. The multiPRS developed in ADVANCE was validated in participants with type 2 diabetes in the UK Biobank [27] and three smaller external cohorts. We also assessed whether this approach could help in early identification of individuals who could benefit most from the intensive therapy such as administered in ADVANCE [28, 29].

## Methods

### Ethics

The ADVANCE, CLINPRADIA, CanPath and post-MONICA studies were approved by the ethics commitees of their coordinating centres and by each participating centre. Only participants who provided written informed consent to the genetic sub-studies took part in the analysis. A material transfer agreement was signed with UK Biobank that covers Research Tissue Bank (RTB) under projects 49731 and 59642. The data analysis was approved by the ethics committee of the Centre hospitalier de l’Université de Montéal (CHUM).

### Patient cohorts

Five cohorts were studied here, details of which can be found in the electronic supplementary material (ESM) Methods. ADVANCE (Action in Diabetes and Vascular Disease: Preterax and Diamicron MR Controlled Evaluation) was used to construct the prediction model and to assess its clinical utility. It was a 2 × 2 factorial design, RCT of BP lowering (perindopril-indapamide vs placebo) and intensive glucose control (gliclazide-MR-based intensive intervention with a target of 6.5% [48 mmol/mol] HbA_1c_ vs standard care) in individuals with type 2 diabetes (ClinicalTrials.gov registration no. NCT00145925). A total of 11,140 participants were recruited from 215 centres in 20 countries. Participants were older than 55 years and diagnosed with type 2 diabetes after the age of 30 years. ADVANCE-ON was a 5 year post-trial observational extension of ADVANCE conducted in 80% of participants [26]. Here, we studied a subset of 4098 genotyped individuals of European descent with type 2 diabetes whose clinical characteristics are summarised in ESM Table 1. Data from the UK Biobank [18] were used to validate the model developed in ADVANCE and construct an independent model in 17,604 participants of white British origin with type 2 diabetes (ESM Fig. 1, ESM Table 1). The model was validated in three independent cohorts with phenotypes available in each one. The Czech post-MONICA (the WHO Monitoring Trends and Determinants in Cardiovascular Disease) study was a cross-sectional survey investigating the determinants of cardiovascular risk factors in a 1% random sample of the general population in nine districts of the Czech Republic, stratified by age and sex [30]. Among the 502 individuals genotyped, 106 had albuminuria. CLINPRADIA (Management of Albuminuria in Hypertensive Diabetics) (ClinicalTrials.gov registration no. NCT 01907958) was a multicentre study to evaluate the management of microalbuminuria in hypertensive patients with type 2 diabetes in Canada. The study was performed in 230 individuals with type 2 diabetes (mean age 67 years). Forty per cent of participants had albuminuria at study entry. CanPath (Canadian Partnership for Tomorrow’s Health) brings together five Canadian regional cohorts: 488 (mean age 58 years) individuals of European origin with type 2 diabetes were analysed here.

### Outcomes description

Macroalbuminuria is defined as urinary albumin/creatinine ratio (UACR) of >33.9 mg/mmol (300 mg/g). Low eGFR is defined as eGFR below 60 ml min^−1^ [1.73 m]^−2^. New or worsening nephropathy is defined as the development of macroalbuminuria, doubling of serum creatinine to a level of at least 200 mmol/l, or end-stage renal disease (ESRD). ESRD is defined as the need for dialysis or renal transplantation, or death due to renal disease. The outcome ‘major microvascular events’ is a composite of ESRD, defined as the requirement for renal replacement therapy, death from renal disease, requirement for retinal photocoagulation or diabetes-related blindness in either eye. The outcome ‘major macrovascular events’ is a composite of non-fatal myocardial infarction, non-fatal stroke or cardiovascular death. The outcome ‘combined major microvascular or macrovascular events’ is defined as death from CVD, non-fatal stroke or non-fatal myocardial infarction, and new or worsening renal or diabetic eye disease.

### Creation of wPRS and multiPRS

We identified 26 factors and outcomes that we grouped into ten groups of risk/outcomes: diabetes, obesity, BP, albuminuria, GFR, biomarkers, lipids, stroke, CVDs and low birthweight, with SNPs obtained from 47 publications cited in ESM Table 2 of large-scale GWAS consortia conducted in hundreds of thousands of individuals of European descent. We used their summary statistics, included in the National Human Genome Research Institute GWAS Catalog and HuGE navigator (https://www.ebi.ac.uk/gwas/home), and extracted 598 SNPs, listed in ESM Table 3 together with their effect size (β). Descriptive summary statistics were computed, using frequencies (%) for categorical variables and means (±SD) for continuous variables. A binomial test was used to compare the two proportions of categorical variables. We constructed 10 wPRS for the ten risk groups, as different SNPs contribute with different weights to the PRS value, by summing the product of the number of risk alleles for each participant by the effect size of those SNPs, i.e.${wPRS}_i^k={\sum}_{j=1}^m{X}_{ij}^k\times {\upbeta}_j^k$, where ${X}_{ij}^k$ is the allele frequency of *i*^*th*^ subject in *j*^*th*^ SNP for *k*^*th*^ phenotype and β is the effect size attributed to the SNP for the same phenotype in the original GWAS (ESM Figs 2, 3 and ESM Table 3). The effect size attributed to each of the 598 SNPs was obtained from the same group of complications or risk factors. As an example, the effect size of a SNP associated with diabetes was used in the generation of the PRS_diabetes_ only. If the same SNP was also shown to be associated with albuminuria in meta-analyses, the effect size used in the other PRS was the one from the original meta-analysis of albuminuria and not the effect size derived from the meta-analysis of diabetes. Variants in five genes (*TCF7L2*, *ADCY5*, *FTO*, *GCKR* and *HNF1A*) were used in two PRSs. The number of SNPs or the unit used is not the same for the 26 predictors, so the wPRS had to be scaled by the sum of its effect coefficients and multiplied by the number of loci of that specific trait. With this scaling, each risk predictor will have an equivalent weight at an equivalent number of loci. The 10 wPRS, sex, PC1 of ethnicity, age at onset and diabetes duration were included as covariates in a logistic regression model, that we named multiPRS model, to classify prevalent or to predict new type 2 diabetes complications as illustrated in ESM Fig. 3. For clarity, we generated a PRS for each of the ten following traits: PRS_diabetes_, PRS_obesity_, PRS_blood pressure_, PRS_albuminuria_, PRS_glomerular filtration rate,_ PRS_biomarkers_, PRS_lipids_, PRS_stroke_, PRS_cardiovascular diseases_ and PRS_low birthweight_. These 10 wPRS were constructed as described above and the effect sizes from the logistic regression model for each of these 10 wPRS are shown in ESM Fig. 3.

We developed the model using the ADVANCE cohort (referred to herein as the ADVANCE model) and compared the accuracy of the model using C-statistics with tenfold cross-validation with a 10–90 data split of ADVANCE, to avoid overfitting. We tested the model in the UK Biobank (used as an external validation dataset) and three other smaller cohorts of individuals with type 2 diabetes, using pROC package (https://cran.r-project.org/web/packages/pROC/pROC.pdf) in R. Given the size of the UK Biobank resource, an alternative (white British-specific) model was also fit to the UK Biobank samples, with tenfold cross-validation, to replicate the approach (referred to herein as the UK Biobank model).

### Statistical analyses

MultiPRS thirds and treatment effects were examined through cumulative hazards curves with the use of Cox proportional hazard models. The logrank test was used to compare the cumulative hazards over the period of 9.5 years (ADVANCE and ADVANCE-ON) to examine trial and post-trial effects of the intensive BP-lowering and intensive glucose therapies on cardiovascular death, all-cause death and ESRD in the three genetic risk groups. The Hosmer–Lemeshow test is used to test for goodness of fit of multiPRS in logistic regression models. The test assesses whether the observed event rates match expected event rates in subgroups of the model population. The subgroups analysed are sex and ethnicity. Unless stated otherwise, a *p* value less than 0.05 is considered statistically significant. Additional details of statistical analyses, genotyping, imputation as well as the stepwise approach for selection of SNPs and creation of multiPRS are included in ESM Methods, ESM Figs 2, 3 and ESM Tables 2, 3.

#### Genotyping and imputation

ADVANCE participants were genotyped using the Affymetrix Genome-Wide Human SNP Arrays 5.0 or 6.0 or the Affymetrix UK Biobank Axiom Arrays (Affymetrix, Santa Clara, CA, USA). The genotype calling of participants from UK Biobank was performed by Affymetrix on two closely related purpose-designed arrays: ~50,000 participants were run on the UK BiLEVE Axiom array and the remaining ~450,000 were run on the UK Biobank Axiom array. The post-MONICA samples were genotyped with the Affymetrix Genome-Wide array 6.0 and those from CLINPRADIA with UK Biobank Axiom arrays. Participants in CanPath were genotyped using UK BioBank Axiom or GSA Illumina arrays. Quality control was performed on the final genotypes before imputation as described in ESM Methods [25]. A PC analysis using 34,570 independent SNPs on individuals of European descent in ADVANCE was done with EIGENSOFT 3.0 package (https://github.com/argriffing/eigensoft, version 3.0). It was used to adjust for genetic ethnicity and all individuals from other cohorts were projected onto these PCs (ESM Fig. 4). Only PC1 was used here, as it reflects best the genetic structure from East (Balto-Slavic) and West (Celtic) of Europe within these cohorts. Additional PCs could be needed when expanding to other geo-ethnic groups, such as southern European individuals for which population structure is reflected on second PC (PC2) (ESM Fig. 4).

#### Hierarchical clustering

We performed unsupervised hierarchical clustering (hclust complete method) as described by the R Core Team [31] on the Euclidean distance matrix of the predicted risk values of our models (myocardial infarction, stroke, heart failure, major macrovascular events, cardiovascular and all-cause death). Heatmaps were constructed using R heatmap.2 from gplots library [31, 32].

#### Prediction using clinical risk scores

For comparison between clinical and multiPRS-based scores, we calculated the ADVANCE clinical risk score that includes age at diagnosis, known duration of diabetes, sex, and baseline pulse pressure, treated hypertension, atrial fibrillation, retinopathy, HbA_1c_, UACR and non-HDL-cholesterol [33], and the widely used Framingham risk score (FRS) that includes age, sex, total cholesterol, HDL-cholesterol, smoking status, diabetes, systolic BP and BP treatment as predictors [34].

## Results

### Performance metrics of the multiPRS model as a classifier and predictor of cardiovascular and renal outcomes of type 2 diabetes

**Fig. 1:**
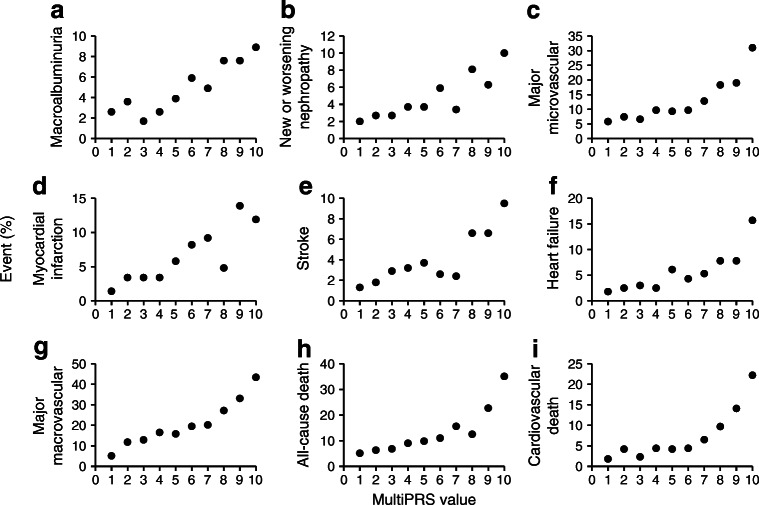
(**a**–**i**) Percentage of events along multiPRS deciles. ADVANCE participants were stratified into equal deciles along multiPRS scoring, from lowest to highest score. Each point represents the percentage of event occurrence in the decile

We developed a multiPRS model using the ADVANCE cohort, with tenfold cross-validation (referred to herein as the ADVANCE model). The baseline characteristics of 4098 individuals with type 2 diabetes in ADVANCE are shown in ESM Table 1. They were recruited in 14 European countries and in Australia, New Zealand and Canada allowing other European-descent cohorts to be projected appropriately onto principal components reflecting population structure (ESM Fig. 4). The percentages of ADVANCE participants who had an outcome including death during the 5 year follow-up increased exponentially according to multiPRS score rising sharply in the last 3 deciles of the distribution (Fig. 1). The ADVANCE model was validated in the UK Biobank and three smaller cohorts of individuals with type 2 diabetes (Table 1). The ADVANCE multiPRS model had similar area under the receiver operating characteristic (ROC) curves, AUCs (95% CI) for classification of prevalent stroke, myocardial infarction, low eGFR and albuminuria in ADVANCE and UK Biobank (Table 1). The AUCs were higher for stroke and myocardial infarction in the CanPath cohort while they were almost identical for albuminuria in ADVANCE, UK Biobank and CLINPRADIA. The multiPRS had a low but significant AUC for albuminuria in individuals with the metabolic syndrome from the general population of post-MONICA. Given the size of the UK Biobank resource (ESM Table 1), the multiPRS model was also fit to the UK Biobank samples, with tenfold cross-validation, to replicate the approach (referred to herein as the UK Biobank model). The UK Biobank model’s AUCs were similar to those of the ADVANCE model when tested in the UK Biobank (Table 1). These results show that the multiPRS model developed in ADVANCE had a similar discrimination power for cardiovascular and renal complications of type 2 diabetes in different patient cohorts.

**Table 1:** Performance (AUC) of the multiPRS model for prevalent and incident cases of T2D complications in different cohorts

T2D complication	Training/testing cohorts		Validation cohorts			
	ADVANCE model *n* = 4098	UKBB model *n* = 17,574	UK Biobank *n* = 17,574	CanPath *n* = 488	CLINPRADIA *n* = 230	Czech post-MONICA *n* = 502
Stroke	0.61 (0.59, 0.64)	0.61 (0.59, 0.63)	0.59 (0.57, 0.60)	0.80 (0.63, 0.97)	–	–
Myocardial infarction	0.58 (0.56, 0.60)	0.67 (0.66, 0.68)	0.63 (0.62, 0.64)	0.78 (0.68, 0.89)	–	–
Low eGFR	0.72 (0.70, 0.74)	0.70 (0.69, 0.72)	0.67 (0.65, 0.69)	–	–	–
Macroalbuminuria	0.63 (0.59, 0.68)	0.65 (0.62, 0.69)	0.63 (0.59, 0.66)	–	0.62 (0.53, 0.71)	0.56 (0.50, 0.62)
Incident stroke	0.62 (0.58, 0.67)	0.65 (0.62, 0.67)	0.62 (0.59, 0.65)	–	–	–
Incident myocardial infarction	0.64 (0.61, 0.68)	0.65 (0.63, 0.67)	0.61 (0.59, 0.64)	–	–	–

Data expressed as AUC (95% CI)

30 participants with missing genotypes were excluded from the analysis of UK Biobank

The multiPRS model is composed of the 10 wPRS, PC1, sex, age at diagnosis and diabetes duration. The classification of cases vs non-cases of cardiovascular and renal complications of T2D by the multiPRS model was assessed in parallel in the ADVANCE (ADVANCE model) and the UK Biobank (UKBB model) sets. Incident cases are defined as having an outcome during the study (free of outcome at baseline), and control participants were those who did not have a specific outcome at any time during the study. AUCs and percentile-based CIs were estimated from ROC curves and calculated from the predicted risks derived from the regression models with tenfold cross-validation. The predictors of the ADVANCE model (dataset where the model was constructed) were also tested in the UK Biobank that was used as a validation dataset. The ADVANCE model was also assessed in three independent cohorts for complications available in each of them

T2D, type 2 diabetes

The primary outcome of the ADVANCE trial was the composite of major macrovascular and microvascular events as intention-to-treat by intensive control of BP [24, 26, 28, 29]. The AUCs of Table 2 represent the discrimination between incident cases, defined as having an outcome during the ADVANCE trial (free of outcome at baseline), and control participants who did not have a specific outcome at any time during the study. The AUCs were 0.67 (95% CI 0.65, 0.70) for combined micro- and macrovascular events, 0.67 (0.64, 0.70) for microvascular and 0.68 (0.66, 0.70) for macrovascular events (Table 2). The ADVANCE model predicted incident stroke with the same AUC of 0.62 in ADVANCE and UK Biobank (Table 1). It predicted incident myocardial infarction with AUC values of 0.64 (0.61, 0.68) in ADVANCE and 0.61 (0.59, 0.64) in UK Biobank; these AUC values were slightly lower than with the UK Biobank predictive model (AUC = 0.65 for both incident stroke and myocardial infarction) (Table 1).

**Table 2:** Prediction performance and risk stratification thresholds of incident cases in ADVANCE

Prediction of incident cases (*n*)	ADVANCE			High risk (30%)			High risk (10%)		
	PP %	AUC 95% CI	AUC^1^ 95% CI	OR	PPV %	NPV %	OR	PPV %	NPV %
Combined micro- or macrovascular (844)	40	0.67 (0.65, 0.70)	0.71 (0.68, 0.74)	3.1	68	54	3.5	80	60
Major microvascular (334)	13	0.67 (0.64, 0.70)	0.68 (0.64, 0.72)	3.1	28	86	3.7	41	81
Major macrovascular (559)	21	0.68 (0.66, 0.70)	0.73 (0.70, 0.76)	3.1	44	74	3.5	55	66
Stroke (154)	4	0.66 (0.62, 0.71)	0.74 (0.70, 0.79)	3.1	12	94	2.9	16	96
Myocardial infarction (192)	7	0.67 (0.63, 0.70)	0.69 (0.65, 0.74)	2.2	16	92	2.1	19	94
Heart failure (225)	6	0.68 (0.65, 0.72)	0.74 (0.68, 0.78)	3.1	15	93	3.9	29	90
Macroalbuminuria (150)	4	0.65 (0.60, 0.69)	0.67 (0.62, 0.72)	2.3	19	91	2.1	27	97
Low eGFR (1009)	41	0.64 (0.62, 0.66)	0.69 (0.66, 0.72)	4.0	59	55	5.1	74	46
New/worsening nephropathy (198)	5	0.64 (0.60, 0.68)	0.66 (0.62, 0.70)	2.5	27	94	2.5	21	95
Cardiovascular death (283)	7	0.72 (0.69, 0.75)	0.78 (0.74, 0.81)	4.4	20	91	4.7	35	87
All-cause death (549)	13	0.69 (0.67, 0.72)	0.74 (0.72, 0.77)	3.1	33	84	4.4	47	74

The multiPRS model is composed of the 10 wPRS, PC1, sex, age at diagnosis and diabetes duration. The number of cases as well as the period prevalence (PP) of each event during the 5 year follow-up of ADVANCE are indicated. AUC represents the discrimination between incident cases, defined as having an outcome during the 5 years of ADVANCE (free of outcome at baseline), and control participants who did not have a specific outcome at any time during the ADVANCE trial. AUC^1^ was calculated using a control group that includes normotensive participants only. PPV and NPV were adjusted for the prevalence of the specific outcome. OR: frequency of a specific outcome in high-risk group/frequency of the outcome in the remainder of the population

Outcomes are defined in the Methods section

Higher AUCs (AUC^1^ in Table 2) were observed for most outcomes when cases were compared with normotensive individuals who did not have a specific outcome at any time during the study with AUCs^1^ around 0.70 for most cardiovascular outcomes and death. Adjustment for treatment assignment did not modify the AUC values (ESM Table 4). The multiPRS model was well calibrated (expected vs observed event rates are similar) for cardiovascular death in the whole population (π = 0.67) with better fit (the closer the π value is to 1, the better the fit) for men (π = 0.66) than women (π = 0.48) and for Slavic (π = 0.77) than Celtic (π = 0.44) individuals. The best fit was observed for all-cause death, π-values exceeding 0.8 in both Celtic (West of Europe) and Slavic (East of Europe) individuals (ESM Fig. 5).

These results indicate that the multiPRS model can predict individual as well as combined cardiovascular and renal complications of type 2 diabetes before these complications appear.

### Risk stratification

**Fig. 2:**
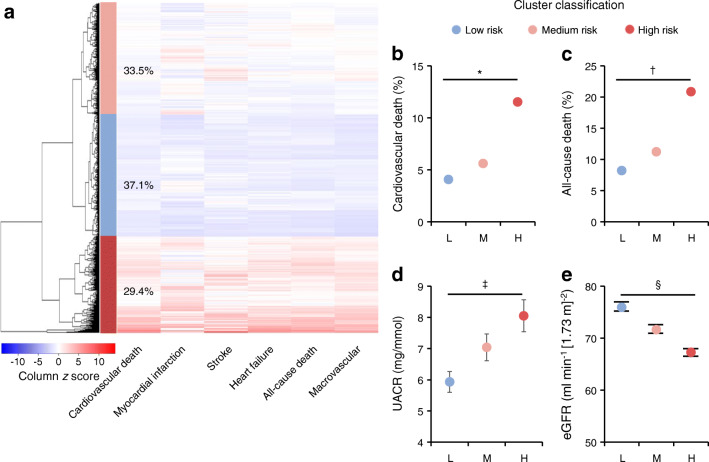
(**a**–**e**) Clustering of combined macrovascular disease risk by multiPRS using unsupervised hierarchical clustering algorithm. This clustering method identified three main clusters of individuals with *low (blue; L),* medium (pink; M) or *high* (red; H) combined macrovascular risk representing 37.1%, 33.5% and 29.4%, respectively, of ADVANCE participants. (**a**) The multiPRS values for each participant and each outcome are represented by *z* score (blue colour: negative score, red colour: positive score) in the heat map. (**b**, **c**) The incidence (%) of cardiovascular (*p = 1.5 × 10^−13^) (**b**) and all-cause death (^†^*p* = 1.8 × 10^−21^) (**c**) were compared between the high and the low clusters. (**d**, **e**) Differences in UACR (^‡^*p* = 1 × 10^−4^) (**d**) and eGFR (^§^*p* = 2 × 10^−44^) (**e**) values were determined between the high and the low clusters. eGFR is based on CKD-EPI formula

In order to determine the optimal risk thresholds, we explored two possible cut-offs: one of 10% that corresponds to the last decile of multiPRS score shown in Fig. 1 and a higher threshold of 30% that corresponds to the last 3 deciles. As expected, the positive predictive value (PPV) was higher for the top 10% than the 30% high-risk threshold (Table 2). However, when the frequency of the high risk is lower than the prevalence of the complication, as in the case of total death, low eGFR, micro- and macrovascular events and their combination that have prevalence higher than 10%, the PPV is high but the sensitivity (% of people who are at risk are detected) is low, and the 10% high-risk threshold will identify a lower percentage of individuals who will have an outcome. False negatives must be minimised and moderate PPV (with its greater proportion of false positives) is more acceptable as no harm is likely to be done in protecting patients against a diabetic complication even if that complication would not occur without it. The 30% threshold showed in general similar or even better negative predictive values (NPVs) than the 10% threshold. Furthermore, the ratio of events between the 10% and 30% thresholds and the remainder of the population demonstrated a clear enrichment of at-risk individuals with no clinically relevant differences between the two thresholds. For instance, the ADVANCE participants in the 30% high-risk threshold had a mean of 3.1-fold increased risk of major micro- and macrovascular events (*p* = 6.3 × 10^−21^ and *p* = 9.6 × 10^−31^, respectively), and a 4.4-fold (*p* = 6.8 × 10^−33^) increased risk of cardiovascular death than the remainder of participants. The pertinence of the 30% high-risk cut-off was confirmed by unsupervised hierarchical clustering analysis that identified three main clusters of individuals representing 37.1%, 33.5% and 29.4% of ADVANCE individuals having a *low*, medium or *high* risk of major macrovascular events including myocardial infarction, stroke, heart failure, and cardiovascular death (Fig. 2a). Twenty per cent of individuals in the high multiPRS stratum have died during the 5 years of the ADVANCE trial compared with 8% in the low multiPRS category (Fig. 2c). The difference between these two groups was also highly significant for renal events known to contribute to the high mortality rate in high-risk individuals (Fig. 2d,e).

### Contribution of genomic and non-genomic factors to the risk prediction model

**Fig. 3:**
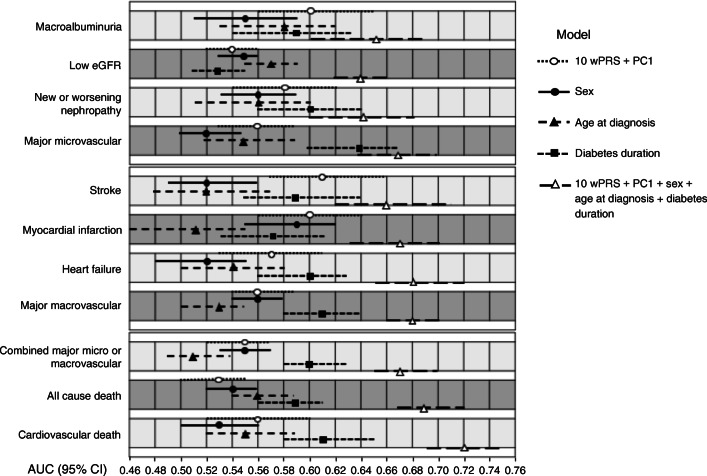
Contribution of genomic and non-genomic factors to the risk prediction model. AUCs (95% CI) are shown for incident complications (free of outcome at study entry). White circles indicate AUCs with genomic component 10 wPRS + PC1 only; black circles indicate AUCs with sex alone; black triangles represent AUCs with age at diagnosis alone; black squares are AUCs with diabetes duration alone; white triangles indicate AUCs of the full model. Upper section: microvascular and renal outcomes; middle section: macrovascular and cardiac outcomes; lower section: combined micro- and macrovascular outcomes and death

We conducted several complementary analyses to assess the contribution of the different factors to the prediction performance. For the outcomes for which we had a single wPRS developed specifically for the trait, we could determine that: (1) each one of the 10 wPRS contributed significantly to at least one of these traits (ESM Table 5); (2) the contribution of a trait-specific PRS was generally more important than the contribution of the other PRSs; (3) the outcomes were best predicted by more than one PRS; and finally, (4) the combination of 10 wPRS improved the AUCs (ESM Table 5). We previously reported significant differences in several of the diabetes outcomes between Europeans of Slavic and Celtic origins that can be stratified by PC1 of genetic diversity [25]. Here, we are showing that PC1 by itself had a variable impact on the AUC values, but its addition generally improved the prediction performance of the model. (ESM Table 5). Negligible changes were noted here by adding more PCs of ethnicity. The genomic component (10 wPRS and PC1) was generally more predictive than sex or age at onset of diabetes (Fig. 3). Diabetes duration had a higher AUC than the 10 wPRS particularly for heart failure, micro- and macrovascular events and death. The combination of genomic and non-genomic determinants yielded the model with the highest predictive performance (Fig. 3).

**Fig. 4:**
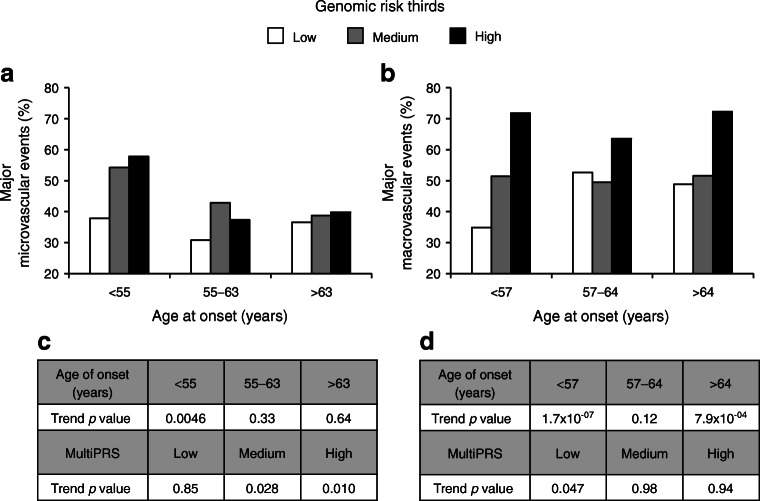
Frequency of major microvascular and macrovascular events by genomic (10wPRS + PC1) and age at onset of diabetes strata. ADVANCE participants were stratified into equal thirds of low, medium and high genomic risk strata and of <55, 55–63 and >63 years of age at diagnosis of diabetes. The control participants used are normotensive individuals with no major microvascular (**a**) and macrovascular (**b**) events at any time during the 4.5 year follow-up of the ADVANCE study. ORs were calculated between high and low genomic component of the multiPRS (OR 1.53 [95% CI 1.08, 2.17] *p* = 0.017) and between age at onset >63 years and <55 years (OR 0.61 [0.43, 0.87] *p* = 0.0057) for microvascular events. For macrovascular events, the ORs between high and low genomic component of the multiPRS were (OR 2.78 [2.02, 3.81] *p* = 2.6 × 10^−10^) and (OR 1.22 [0.91, 1.64] *p* = 0.19) between age at onset >64 years and <57 years. (**c**, **d**) The trend testing was done within formal regression analysis using parametric method separately for different age categories and genomic strata. Major macrovascular and major microvascular events are defined in the Methods

Figure 4a shows that the highest risk of microvascular events was seen in carriers of high genomic (10 wPRS and PC1) risk with OR _High vs Low_ = 1.53 (95% CI 1.08, 2.17), *p* = 0.017 and younger age at onset of diabetes (OR _Old vs Young_ = 0.61 [0.43, 0.87], *p* = 0.0057). This contrasts with macrovascular events, for which the highest risk was seen in the highest genomic risk group with OR _High vs Low_ = 2.78 (2.02, 3.81), *p* = 2.6 × 10^−10^ independently of the age at onset of diabetes (Fig. 4b). It is noteworthy that the stratification capacity of the genomic component of the model was best in people with earlier onset of diabetes for both major micro- and macrovascular events, as shown by the p_trend_ values of 4.6 × 10^−3^ for major microvascular and 1.7 × 10^−7^ for major macrovascular events (Fig. 4c, d).

### Clinical utility of the multiPRS model

The multiPRS model did not perform better than the ADVANCE clinical score, knowing that the latter must be considered ‘optimistic’ as our subset of patients was part of the population from which it was developed and included such clinical outcomes as atrial fibrillation, albuminuria and low eGFR (ESM Table 6). However, its AUC values were generally higher than those of the FRS. Even though our primary aim was not to develop a model that outperforms existing clinical scores but one that can predict before symptoms appear, it should be noted that the multiPRS improved the prediction of diabetes outcomes of the two clinical scores. For instance, the continuous net reclassification index (NRI) was 45% for myocardial infarction and 62% for cardiovascular death of people initially classified by the FRS, and was 41% for myocardial infarction and 26% for cardiovascular death of those initially classified with the ADVANCE clinical score (ESM Table 7).

**Fig. 5:**
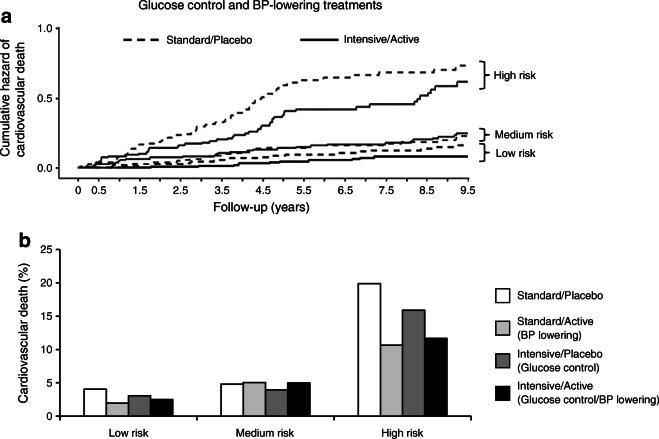
Cumulative hazard plots of cardiovascular death stratified by multiPRS strata and glucose and BP-lowering treatments. (**a**) Adjusted cumulative hazard curves for 9.5 year cardiovascular death by combined active BP and intensive glucose-lowering treatment arms in the high, medium and low multiPRS thirds. The control participants used are normotensive individuals. The effect of BP and glucose-lowering treatment was assessed using HRs and was significant for individuals included in the high-risk group (HR 0.61 [95% CI 0.40, 0.93], *p* = 0.021 at year 4.5 follow-up of ADVANCE trial, and HR 0.67 [0.47, 0.95], *p* = 0.023 at year 9.5 follow-up of ADVANCE-ON follow-up). (**b**) Frequency (%) of cardiovascular death in low, medium and high multiPRS risk strata and risk reduction by treatment arms in ADVANCE. The four treatment arms are Standard/Placebo, Standard/Active for BP lowering, Intensive/Placebo for glucose control and Intensive/Active for glucose control and BP lowering, respectively. The NNT is 64, *p* = 0.28 in the low multiPRS third compared with NNT 12, *p* = 0.0062 in the high-risk group for the combined therapy in ADVANCE

The cumulative incidence of death was significantly different (*p* < 0.0001) between individuals with low, medium and high predicted risk (ESM Fig. 6). We also noted that the intensive BP control achieved during ADVANCE led to a significant reduction of total death (HR 0.797, *p* = 0.046) and cardiovascular death (HR 0.677, *p* = 0.009) in individuals within the highest third of predicted risk, and these reductions remained significant during ADVANCE-ON (ESM Fig. 6, left panel). No such benefit was observed with intensive glycaemic control (ESM Fig. 6, right panel), while glucose control reduced ESRD in individuals carrying the highest predicted risk values (HR 0.345, *p* = 0.043 in ADVANCE), remaining significant at the end of ADVANCE-ON (HR 0.455, *p* = 0.026) (ESM Fig. 7). Fifty-nine per cent of ESRD cases occurred in the highest multiPRS third (ESM Fig. 7). The reduction of cardiovascular death by ADVANCE therapy occurred mainly in the high-risk group (HR 0.61 [95% CI 0.40, 0.93], *p* = 0.021) and remained significant during ADVANCE-ON (Fig. 5a) and the number needed to treat (NNT) to prevent one cardiovascular death could be reduced by as much as fivefold. For instance, NNT = 12 (*p* = 0.0062) in the high-risk third compared with NNT = 64 (not significant) in the low-risk third (Fig. 5b).

## Discussion

Novel evidence suggests that for many adult-onset common diseases, a significant degree of the heritability could be captured with a large number of common SNPs identified through GWAS [35]. Diabetes is associated with microvascular and macrovascular outcomes and the line of demarcation between their pathogenetic mechanisms is blurred [19–21]. For instance, in ADVANCE, we previously reported that increases in UACR or decreases in eGFR in individuals with type 2 diabetes were independent predictors of cardiovascular events and death [22]. More recently, we showed that the combination of changes in both eGFR and UACR is a better predictor of major macrovascular events than when the two are assessed separately [23]. Other groups showed that a clinical score that captures different types of complications is more powerful in predicting mortality risk than a simple count of complications [36]. Furthermore, we reported a polygenic overlap between ischaemic stroke and kidney function [37], and a shared genetic architecture has been revealed between type 2 diabetes and BP regulation [10]. We included SNPs associated with micro- and macrovascular outcomes in addition to their common risk factors in our multiPRS and provide evidence that combining 10 wPRS of related traits with sex, PC1, age at onset and diabetes duration into a joint prediction model (named here multiPRS model) allows the prediction of both microvascular and macrovascular endpoints of type 2 diabetes. Our study provides the first evidence that combining 10 wPRS of related traits in a joint model could optimise the prediction of microvascular and macrovascular endpoints of type 2 diabetes. The accuracy and generalisation of prediction of cardiovascular and renal outcomes of diabetes with the multiPRS was shown in individuals with type 2 diabetes of five independent cohorts (Table 1).

Our multiPRS model yielded an AUC of 0.65 for incident stroke in UK Biobank. An AUC of 0.64 was recently reported with a metaGRS (defined as multiple GRSs combined into one meta-score) used to predict ischaemic stroke in the general white British population of the UK Biobank [18, 27]. Our prediction model was slightly superior to the genetic risk score derived from 204 variants associated with coronary risk published by ACCORD, a clinical trial with a design similar to that of ADVANCE, [38] suggesting that integration of 10 wPRS of related traits into a multiPRS model could improve the performance of the model.

The prediction model developed here outperformed the FRS, and improved the individual 5 year risk reclassification when added to the ADVANCE and FRS clinical scores but, more importantly, it can be used well before the apparition of outcomes. In ADVANCE, we observed that the penetrance of outcomes differs between macro- and microvascular complications [39]. Here, we are showing that, for macrovascular events, the greatest risk was in the oldest age groups with the longest duration of diabetes. By contrast, for microvascular events, the highest risk was also seen with the longest duration of diabetes, but in people with the youngest onset of diabetes (Fig. 4). Individuals in the high-risk group had a diagnosis of diabetes 3 years younger (57 years) than those of the low-risk group (60 years) for microvascular events, while for macrovascular events, the age at onset of diabetes was 62 years in the high-risk and 58 years in the low-risk groups. Finally, the efficacy of stratifying participants along a gradient of multiPRS was better seen in younger participants or in those with early onset of type 2 diabetes for both micro- and macrovascular complications, suggesting that the usefulness of a multiPRS is in primary prevention before target organ damage occurs.

Clinical utility of genetic risk scores emerged over the past few years with the demonstration that individuals in the highest genetic risk category had the largest clinical benefit from therapy [40]. The capacity to detect individuals with the best response to medication is one of the most important results of our study. Figure 5 illustrates three components of this study: (1) individuals classified into the low multiPRS category did not benefit from intensive therapies compared with participants of the higher multiPRS thirds; (2) the combination of intensive glucose and BP control showed the best reduction in risk, as reported in ADVANCE [29]; and finally, (3) the highest thirds of multiPRS had the lowest NNT with combined therapies of ADVANCE, i.e. PRS risk classification is clinically effective in reducing the burden of the disease. A limitation of our study is that ADVANCE participants were older than 65 years at entry or 55 years if they had a substantial elevated risk of cardiovascular disease. The possibility that all individuals with newly diagnosed type 2 diabetes could benefit from our multiPRS screening should be explored further. The UK Prospective Diabetes Study showed that at diagnosis, 20% of individuals already had diabetes-related complications. We are showing here that our prediction model could identify individuals with albuminuria in the prediabetes phase of the post-MONICA study suggesting that individuals at high risk for diabetes should also be screened using the multiPRS model.

We recently completed a cost–utility analysis to evaluate the practical implications of the prediction of pre-ESRD and death with the multiPRS vs usual albuminuria screening from a Canadian healthcare system and societal perspective. The analysis showed that, for a lifetime horizon, the polygenic risk scoring was less expensive and more efficacious in terms of quality adjusted life years than usual screening [41].

Our multiPRS scoring is generalisable to individuals of European descent, and our multi-ethnic study in preparation suggests a potential of applying our model to diverse populations. It is possible that, in the future, more SNPs will be added and that this, in addition to more complex machine learning models that would capture non-linear effects between the wPRS and existing clinical predictors, will further improve the predictive power [42]. The possibility that our multiPRS model could predict multimorbidity in non-diabetic individuals should also be explored, as recent evidence demonstrated that novel classes of glucose-lowering medication such as SGLT2 class, which improves heart failure in individuals with diabetes, are also effective in individuals without diabetes [43], suggesting common determinants. The polygenic prediction model developed here is based on common genomic variants that are present at birth, and a few reliable demographic variables that are routinely collected during clinical practice without requiring the presence of any clinical manifestations or initial outcomes, i.e. in the pre-symptomatic phase, suggesting its usefulness in primary prevention. It could be applied in a trial to help identify participants who could benefit from novel glucose-lowering treatments or high-risk individuals for cardiovascular outcomes trials for type 2 diabetes medications by reducing cost or time to obtain sufficient endpoints to allow a better estimate of risk.

## Supplementary Information

ESM (PDF 1997 kb)

## Data Availability

The list of SNPs used to generate the multiPRS model is included in ESM Table 3. The list of GWAS data is referenced in ESM Table 3. Other data are available on request from JT or PH. Parts of this work were presented in Late Breaking abstract form at the 78th Scientific Sessions of the American Diabetes Association, Orlando, FL, USA, 22–26 June 2018. A preliminary report of the work was made publicly available in MedRxiv on 19 November 2019.

## Abbreviations

ESRD: End-stage renal disease
FRS: Framingham risk score
GWAS: Genome-wide association studies
MultiPRS: Multi-polygenic risk score
NNT: Number needed to treat
NPV: Negative predictive value
PC: Principal component
PPV: Positive predictive value
PRS: Polygenic risk score
ROC: Receiver operating characteristic
UACR: Urinary albumin/creatinine ratio
wPRS: Weighted PRS
