# Supplementary material for: Polygenic risk scores predict diabetes complications and their response to intensive blood pressure and glucose control
Source: Diabetologia. 2021 Jul 6;64(9):2012–25. doi: 10.1007/s00125-021-05491-7 (PMC8382653; doi:10.1007/s00125-021-05491-7)
Supplement: Supplementary file 1 — (PDF 1997 kb) [file 125_2021_5491_MOESM1_ESM.pdf]

Electronic Supplementary Material

Polygenic risk score predicts diabetic complications and their response to intensive blood pressure and glucose control

J. Tremblay, M. Haloui, R. Attaoua, R. Tahir, C. Hishmih, F. Harvey, F.-C. Marois-Blanchet, C. Long, P. Simon, L. Santucci, C. Hizel, J. Chalmers, M. Marre, S. Harrap, R. Cífková, A. Krajčoviechová, D. Matthews, B. Williams, N. Poulter, S. Zoungas, S. Colagiuri, G. Mancia, D.E. Grobbee, A. Rodgers, L. Liu, M. Agbessi, V. Bruat, M-J. Favé, M. Harwood, P. Awadalla, M. Woodward, J. Hussin, and P. Hamet

Table of Contents

Supplementary Methods 3

Supplementary Tables 10

ESM Table 1: Characteristics of ADVANCE and UK Biobank (UKBB) diabetic participants of European descent at baseline. 11

ESM Table 2: SNPs are selected from literature and divided into 10 risk/outcome groups of complications of T2D. 12

ESM Table 3: List of 598 SNPs included in our model. 13

ESM Table 4: Effect of adding treatment assignment as a covariate on the performance of the multiPRS model. 31

ESM Table 5: Effect of individual wPRS, combined 10wPRS and 10wPRS + PC1 on prevalent and incident cases determined by single wPRS. 32

ESM Table 6: AUC with confidence intervals for multiPRS model compared to ADVANCE and Framingham clinical scores. 33

ESM Table 7: Continuous net reclassification index adding the multiPRS to Framingham & ADVANCE clinical risk scores. 34

Supplementary Figures 35

ESM Fig. 1: Flow of selection of individuals with T2D of UK Biobank (UKBB) and number of cases and controls for stroke, myocardial infarction, low eGFR and macroalbuminuria analyses. 36

ESM Fig. 2: Stepwise approach for selection of SNPs included in the 10 wPRS. 37

ESM Fig. 3: Prediction model construction and testing in ADVANCE and UKBB and validation in three independent cohorts of patients with T2D. 39

ESM Fig. 4: Population structure of the study cohorts using principal component analysis projection. 40

ESM Fig. 5: Hosmer-Lemeshow test. 42

ESM Fig. 6: Cumulative hazard plots of all cause and cardiovascular death stratified by multiPRS thirds in standard and intensive blood pressure (left) and glucose (right) treatment arms. 43

ESM Fig. 7: Cumulative hazard plots of ESRD stratified by multiPRS thirds in standard and intensive glucose treatment arm. 44

Supplementary References 45

Supplementary Methods

Combination of hundreds of polymorphisms, whether or not combined with clinical features constitute a novel approach to develop polygenic risk scores.(1)

We applied this technology and tested it on samples from ADVANCE trial and of the UK Biobank of which detailed study methods have been published elsewhere.(2,3)

Patient cohorts

ADVANCE was a 2x2 factorial design, randomized controlled trial of blood pressure (BP) lowering (perindopril-indapamide *vs* placebo) and intensive glucose control (gliclazide MR-based intensive intervention with a target of 6.5 (48 mmol/mol) HbA1c *vs* standard care) in patients with T2D (NCT 00145925). A total of 11,140 participants were recruited from 215 centers in 20 countries. They were older than 55 years and diagnosed with T2D after the age of 30 years. The trial was successful in decreasing total and cardiovascular mortality by attenuation of combined microvascular and macrovascular primary outcome with blood pressure control (4) and renal events and all-cause death with combined control of blood glucose and blood pressure.(5) ADVANCE-ON was a 5-year post-trial observational extension of ADVANCE conducted in 80% of subjects.(6) Here, we studied a subset of 4,098 genotyped T2D patients of European descent from ADVANCE.

UK Biobank: This study is part of projects 49731 and 59642.(3) The UK Biobank study started in 2006 and, until 2010, recruited more than 500,000 participants from the general UK population, aged between 40-69 at recruitment (identified as white British ancestry subset by self-declaration (Field ID 21000)). We selected 18,853 individuals with diabetes diagnosed by a doctor (Field ID 2443). To avoid potential individuals with type 1 diabetes, we excluded individuals whose age at diagnosis of diabetes was less than 30 years old (Field ID 2976) and obtained 17,604 individuals with T2D of white British origin confirmed genetically by principal component analysis (ESM Fig. 1). Phenotype data collected included year of birth (Field ID 34), age at recruitment (Field ID 21022), sex (Field ID 31), genetic sex (Field ID 22001), genetic ethnic grouping (Field ID 22006), diabetes diagnosed by doctor (Field ID 2443), age diabetes diagnosed (Field ID 2976), age when attended assessment centre (Field ID 21003). These last 2 phenotypes allowed us to calculate the duration of diabetes (derived phenotype). We also, collected data related to stroke (age stroke diagnosed in Field ID 4056), date of ischaemic stroke (Field ID 42008), date of stroke (Field ID 42006), source of ischaemic stroke report (Field ID 42009) and, source of stroke report (Field ID 42007)) and, myocardial infarction (age heart attack diagnosed (Field ID 3894), date of nstemi (Field ID 42004), date of stemi (Field ID 42002), date of myocardial infarction (Field ID 42000),

source of nstemi report (Field ID 42005), source of stemi report (Field ID 42003), source of myocardial infarction report (Field ID 42001) and, vascular heart problems diagnosed by doctor (Field ID 6150)). To calculate UACR and eGFR (using CKD-Epi formula), it was necessary to obtain creatinine plasma and urinary levels (Field ID 30510), microalbuminuria (Field ID 30500) and creatininuria (Field ID 30700). For systolic blood pressure, we used the automated reading (Field ID 4080), same for diastolic blood pressure (Field ID 4079), medications blood pressure or diabetes or exogenous hormones (Field ID 6153), and medications for cholesterol, blood pressure or diabetes (Field ID 6177) were used to define hypertensive participants.

The *Czech post-MONICA* study was a cross-sectional survey investigating the determinants of cardiovascular risk factors in a 1% random sample, stratified by age and gender, of the general population in nine districts of the Czech Republic. A total of 3,612 individuals aged 25–64 years were examined in 2007–2009 as previously reported.(7) 502 individuals were genotyped and among those, 106 had albuminuria.

Clinpradia study (NCT 01907958) was a multicenter study to evaluate the management of microalbuminuria in hypertensive patients with T2D in Canada. Its primary objective was to assess the impact of a point-of-care testing for urine albumin excretion on treatment of hypertensive T2D patients as measured by blood pressure levels and albuminuria in the context of primary care setting. The study was performed in 2013-2014. 230 patients with T2D were recruited (mean age 67 years old) in general practice clinics of Ontario and Quebec in Canada and followed for a period of 18 months. Forty percent of these 230 participants had albuminuria at study entry.

The *Canadian Partnership for Tomorrow's Project* (CPTP, partnershipfortomorrow.ca) of CANPATH brings together five Canadian regional cohorts: British Columbia Generations Project, Alberta's Tomorrow Project, Ontario Health Study, CARTaGENE (Quebec)(8) and the Atlantic Partnership for Tomorrow's Health. 10,802 subjects have been genotyped up to now and 601 had T2D. 488 (mean age 58 years old) were used to calculate the AUCs for myocardial infarction (28 cases) and stroke (8 cases) after filtering for European origin, no missing data, no mismatch. Controls were defined as T2D individuals with no complications.

Statistical analyses

Descriptive summary statistics were computed, using frequencies (%) for categorical variables and mean (±SD) for continuous variables. A binomial test was used to compare the two proportions of categorical

variables. AUCs with 95% confidence interval were calculated by fitting the multivariable logistic regression over the PRS, including genetically determined ethnicity (PC1). ADVANCE and Framingham risk scores were computed on ADVANCE trial subjects as was done in other studies.(9) Three groups of risk (low, medium, and high) were determined using unsupervised complete hierarchical clustering algorithm on a Euclidian distance matrix of our score predicted risk values in R. Heatmaps were constructed using R heatmap.2 from gplots library.(10,11)

We also divided the study participants into equal deciles along increasing multiPRS values and then thirds representing equal number of individuals carrying *low*, *medium*, or *high* multiPRS values, thirds of age strata and thirds of age of onset of diabetes and analysed the predictive performance of these strata on total and cardiovascular death, micro-and macrovascular events, myocardial infarction and stroke events. A proportion trend test(12) was used to calculate the trend p-values of the stratified data. We used the pROC package in R for the analysis of ROC curves.(13) The areas under the receiver operating characteristics (ROC) curves (AUCs; 95% confidence intervals), calculated from the predicted risks derived from the regression models, were used to assess the predictive performance of the PRS and the two clinical risk scores. The DeLong method was used to calculate the p-value for the comparison of two AUCs of ROC curves.(14) MultiPRS thirds and treatment effects were examined through cumulative hazards curves with the use of Cox proportional hazard models. For participants with more than one outcome event during follow-up, survival time to the first relevant end point was used in each analysis. Participants were censored at the date of their death or for those still alive at the end of the follow-up.(4) The log-rank test was used to compare the cumulative hazards and the plots are shown over the period of 9.5 years (ADVANCE-ON) to examine post-trial effects of the intensive blood pressure-lowering and the intensive glucose therapies on cardiovascular death, all cause death, and end-stage renal disease in the three genetic risk groups. The Hosmer–Lemeshow test is used to test for goodness of fit of multiPRS in logistic regression models. The test assesses whether the observed event rates match expected event rates in subgroups of the model population. The subgroups analyzed are sex and ethnicity.

Genotyping and imputation

Genotyping of the ADVANCE samples was performed using the Affymetrix Genome-Wide Human SNP Arrays 5.0 or 6.0 or the Affymetrix UK BioBank Axiom arrays (Affymetrix, Santa Clara, California, USA) following standard protocols recommended by the manufacturer. A quality control filtering step was applied to the genotype calls as described in our previous work.(15) Additional quality control steps

included coarse-grain stratification to ensure an European ancestry ratio of more than 0.8 (STRUCTURE software)(16), a genetic relatedness check to ensure independent samples (PLINK) and a sex check to ensure genetic accuracy and database integrity.(17)

Quality control was also performed on the final genotypes to remove any SNPs with more than 4% of missing values across the entire cohort and any samples with more than 2% of missing SNP genotypes. A more stringent threshold was used for any SNPs with between 1 and 5% minor allele frequencies (MAF). Low MAF SNPs with more than 1% of missing values were removed prior to the imputation. 4098 samples passed these quality filters.

Three sets of imputation were performed separately for the individuals of ADVANCE who were genotyped either on Affymetrix arrays 5.0, 6.0 or UK Biobank using SHAPEIT(18), IMPUTE2 software,(19) and the 1000 Genomes project(20,21) phase 3 data set as reference. Only SNPs with an imputation quality score greater than or equal to 0.80 were kept as proposed in other studies.(22) Samples of Clinpradia and Post-Monica were imputed with the IMPUTE2 software using the 1000 Genomes project phase 3 data as reference. The samples from the UKBB were imputed with a combination of 1000 Genomes project phase 3, UK10K and Haplotype reference Consortium. Genotypes data within bgen files were converted to Plink2 files (.pgen, .psam, .pvar). Alleles doses of each SNP of interest in T2D patients were obtained from traw files. The traw files were obtained through the conversion of Plink 2 files using the command "--export A-transpose". Alleles doses were weighted by the corresponding regression coefficients (betas) issued from literature or from the ADVANCE model. A few of the 598 SNPs were not available in UKBB database, proxies SNPs were obtained using the LDproxy tool within LDlink webtool (https://ldlink.nci.nih.gov/?tab=ldproxy).

A subset of 34,570 independent SNPs common to all three microchips was selected to perform a principal component (PC)(4) analysis for the ADVANCE study participants of European origin using the EIGENSOFT 3.0 package.(23) The first principal component (PC1) separated the 4098 individuals along a geographical gradient from East (Balto-Slavic) to West (Germano-Celtic) Europe as described previously.(15) Individual PC1 values were added as co-variates in the logistic regression model.

Stepwise approach for selection of SNPs associated to risk factors of complications of T2D

We selected 26 factors of complications of T2D that we divided into 10 wPRS as initially suggested by Ibrahim-Verbaas(9) and modified as described in ESM Table 2. The 10 wPRS include SNPs associated to diabetes, obesity, blood pressure, albuminuria, GFR, biomarker levels, lipids, stroke, cardiovascular,

and birth weight. We identified most of the GWAS that were reported in the NHGRI-EBI GWAS Catalog and using HuGE navigator we extracted all SNPs together with their p values and effect size (β) for the 26 factors listed in ESM Table 3. We also performed manual literature curation and included additional SNPs by relaxing p-values threshold of associations if they had evidence of independent replication and/or were reported in meta-analyses published by major GWAS Consortia for blood pressure, renal function, stroke and lipids. We then defined LD blocks and identified genotyped and imputed tag SNPs (SNPs that are in LD with the lead SNP contained in the block at $R^2 \geq 0{\cdot}8$) using the HapMap CEU samples from 1000 Genomes phase 3. We matched these tag SNPs with SNPs in our genotype database. When a tag SNPs could not be found in ADVANCE, the LD threshold was relaxed to $R^2 \geq 0{\cdot}7$ and closest LD proxy was selected. If no tag SNP could be found, the LD block was removed from analysis. When several SNPs within a class of risk factors were in the same loci, we selected the top SNP with the best *P* value. In some cases, a SNP was associated with more than one trait and it was thus included in more than one risk groups. We identified a total of 598 SNPs (ESM Tables 2 and 3). Their rs number, genomic position, risk group to which they belong and the published reference which describe them are included in ESM Table 3. Our stepwise strategy for selection of SNPs to be included in the multiPRS is illustrated in ESM Fig. 2 and 3.

Creation of the multiPRS model

To create a multiPRS, we constructed, at first, a weighted polygenic risk score (wPRS) for each of the 10 risk groups over all study participants to evaluate the effect of the 598 SNPs (ESM Table 2). We used the additive model, assuming that each SNP is independently associated with risk, to construct wPRS (different SNPs contribute with different weights to the PRS value) according to the effect size (β) attributed to the tested SNPs in the original association study (ESM Fig. 3).

We calculated wPRS for these predictors, as previously described, by summing the product of the number of risk alleles for each patient by the effect size of those SNPs i.e. $\boldsymbol{wPRS_i^k} = \sum_{\boldsymbol{j=1}}^{\boldsymbol{m}} \boldsymbol{X_{ij}^k} \times \boldsymbol{\beta_j^k}$, where $\boldsymbol{X_{ij}^k}$ is the allele frequency of ith subject in j^{th} SNP for k^{th} phenotype and β is the effect size of the phenotype. The effect size attributed to each of the 598 SNPs was obtained from the same group of complications or risk factors. As an example, the effect size of a SNP associated to diabetes was used in the generation of the PRS-Diabetes only. If the same SNP was also shown to be associated to albuminuria in meta-analyses, the effect size used in the other PRS was the one from the original meta-analysis of albuminuria and not the effect size derived from the meta-analysis of diabetes. Variants in five genes

(TCF7L2, ADCY5, FTO, GCKR and HNF1A) were used in two PRS. The number of SNP is not the same for the 26 predictors as well as the unit used, so the wPRS had to be scaled by the sum of its effect coefficients and multiplied by the number of loci of that specific trait.(24) With this scaling, each risk predictor will have an equivalent weight at an equivalent number of loci (ESM Fig. 3). 10 wPRS for ten risk groups of these 26 predictors were combined with sex, age at diagnosis, diabetes duration, and the first PC (PC1) of geo-ethnicity and used as variables in the logistic regression to predict the risk of T2D complications (ESM Fig. 3).

Supplementary Tables

ESM Table 1: Characteristics of ADVANCE and UK Biobank (UKBB) diabetic participants of European descent at baseline.

Characteristic	UKBB (n=17,604)	ADVANCE (n=4,098)
Age — yr; Mean (SD)	61 (7)	67 (7)
Female sex — n (%)	6283 (35.7)	1524 (37.2)
Diagnosis age — yr; Mean (SD)	54 (8)	60 (9)
Diabetes duration — yr; Median (SD)	5 (2-9)	5 (2-10)
BMI — kg/m^2; Mean (SD)	32 (6)	30 (5)
HbA_{1c}— %; Mean (SD)	6.89 (1.23)	7.31 (1.36)
HbA_{1c}— mmol/mol; Mean	51.8	56.4
SBP — mmHg; Mean (SD)	142 (17)	147 (21)
DBP — mmHg; Mean (SD)	82 (10)	82 (11)
History of currently treated hypertension — n (%)	10566 (60)	2635 (64)
eGFR — ml/min/1.73 m^2; Median (IQR)	91.5 (79.3-98.4)	72.9 (61.0-85.7)
eGFR ≥ 90; no (%)	9223 (55)	653 (16)
eGFR 60 to 89; no (%)	6489 (39)	2465 (61)
eGFR < 60; no (%)	1090 (6)	956 (23)
UACR — mg/mmol; Median (IQR)	1.55 (0.86-3.63)	1.55 (0.70-4.49)
UACR < 3.39; no (%)	6714 (73)	2702 (70)
UACR 3.39 to 33.9; no (%)	2136 (23)	996 (26)
UACR > 33.9; no (%)	306 (3)	178 (4)

Abbreviation: **n**: Number of patients, **Yr**: Years, **SD**: Standard deviation, **IQR**: Interquartile range. **BMI**: Body mass index, **HbA_{1c}**: Glycated hemoglobin, **SBP**: Systolic blood pressure, **DBP**: Diastolic blood pressure, **eGFR**: Estimated glomerular filtration rate based on CKD-EPI formula, **UACR**: Urinary albumin creatinine ratio.

ESM Table 2: SNPs are selected from literature and divided into 10 risk/outcome groups of complications of T2D.

Risk/Outcome group	26 factors	Included SNPs	Reference number
**Diabetes**	Type 2 Diabetes, Glycated hemoglobin	**45**	25-31
**Obesity**	Body mass index, Waist circumference, Waist hip ratio	**80**	32-35
**Albuminuria**	Microalbuminuria, Urinary albumin creatinine ratio	**33**	36
**GFR**	Estimated glomerular filtration rate, Plasma creatinine	**50**	37-41
**BP**	Hypertension, Pulse pressure, Systolic blood pressure	**82**	42-45
**Biomarkers levels**	C-reactive protein, Fibrinogen	**19**	46,47
**Lipids**	High density lipoprotein, Low density lipoprotein, Total Cholesterol, Triglycerides	**77**	48
**Stroke**	Stroke	**43**	49-57
**Cardiovascular**	Atrial fibrillation, Coronary artery calcification, Coronary artery disease, Coronary Heart disease, Intracranial aneurysm, Myocardial infarction	**115**	49,56, 58-70
**Birth weight**	Birth weight	**54**	71
**Total SNPs**		**598**	

SNPs are selected from the listed references as adapted from the model described by Ibrahim-Verbaas CA.(9)

Abbreviation: GFR: Glomerular filtration rate, **BP**: Blood pressure.

ESM Table 3: List of 598 SNPs included in our model.

Risk Group	Original SNP	Chr	Position of original SNP (GRCh37)	Locus (Gene)	Functional Consequence	Alleles		EAF	European ancestry values as published		Present in ADVANCE?	Best ADVANCE Proxy	ADVANCE Effect Allele	Risk Factor	Ref	Nb. of Ind
						Effect	Other		β	p-value						
Diabetes	rs10923931	1	120455869	NOTCH2	Intron	T	G	0.10	0.086	6.52E-05	No	rs835576	C	T2D	(25)	47 117
	rs2779116	1	158585415	SPTA1	Intron	T	C	0.27	0.020	2.75E-09	Yes		T	HbA1c	(26)	34 663
	rs340874	1	214159256	PROX1, PROX1-AS1	Intron	C	T	0.50	0.068	7.20E-10	Yes		C	T2D	(27)	127 677
	rs7578597	2	43732823	THADA	Exon non-syn.	T	C	0.91	0.140	4.47E-05	Yes		T	T2D	(25)	47 117
	rs243021	2	60584819	LOC105374754-MIR4432HG		A	G	0.46	0.086	2.90E-15	Yes		A	T2D	(25)	126 456
	rs7593730	2	161171454	RBMS1	Intron	C	T	0.80	0.104	3.70E-08	Yes		C	T2D	(28)	5 643
	rs552976	2	169791438	ABCB11	Intron	G	A	0.64	0.050	8.16E-18	Yes		G	HbA1c	(26)	40 420
	rs7578326	2	227020653	LOC646736	Intron	A	G	0.64	0.113	1.30E-19	Yes		A	T2D	(25)	141 454
	rs1801282	3	12393125	PPARG	Exon non-syn.	C	G	0.86	0.131	8.01E-06	Yes		C	T2D	(25)	47 117
	rs4607103	3	64711904	ADAMTS9-AS2	Intron	C	T	0.76	0.086	2.34E-04	Yes		C	T2D	(25)	47 117
	rs11708067	3	123350211	ADCY5	Intron	A	G	0.80	0.113	9.90E-21	No	rs11720108	C	T2D	(27)	127 677
	rs1470579	3	185529080	IGF2BP2	Intron	C	A	0.30	0.131	2.17E-09	Yes		C	T2D	(25)	47 117
	rs10010131	4	6303022	WFS1	Intron	G	C	0.60	0.104	4.59E-07	No	rs1801214	T	T2D	(25)	47 117
	rs7754840	6	20661250	CDKAL1	Intron	C	G	0.33	0.113	3.11E-15	Yes		C	T2D	(25)	47 117
	rs1800562	6	26123502	HFE	Exon non-syn.	G	A	0.94	0.060	2.59E-20	No	rs115740542	T	HbA1c	(26)	43 778
	rs9472138	6	43809802	LOC107986598	Intron	T	C	0.28	0.058	4.00E-06	No	rs9472135	T	T2D	(29)	63 537
	rs2191349	7	15064309	DGKB-AGMO		T	G	0.50	0.058	1.10E-08	Yes		T	T2D	(27)	127 677
	rs864745	7	28180556	JAZF1	Intron	T	C	0.51	0.095	2.06E-08	Yes		T	T2D	(25)	47 117
	rs4607517	7	44235668	GCK-YKT6		A	G	0.20	0.040		Yes		A	HbA1c	(27)	127 667
	rs4607517	7	44235668	GCK-YKT6		A	G	0.20	0.068	5.00E-08	Yes		A	T2D	(27)	127 667
	rs972283	7	130466854	LOC105375508	Intron	A	G	0.55	0.095	4.40E-10	Yes		G	T2D	(25)	141 454
	rs4737009	8	41630405	ANK1	Intron	A	G	0.24	0.030	6.11E-12	Yes		A	HbA1c	(26)	36 862
	rs896854	8	95960511	NDUFAF6, TP531INP1	Intron, 5'UTR	C	T	0.48	0.095	2.00E-09	Yes		T	T2D	(25)	141 454
	rs13266634	8	118184783	SLC30A8	Exon non-syn.	A	G	0.30	0.019	4.90E-08	Yes		A	HbA1c	(30)	14 618
	rs10811661	9	22133284	CDKN2B-AS1-DMRTA1		T	C	0.83	0.182	1.45E-10	No	rs10965250	G	T2D	(25)	47 117
	rs13292136	9	81952128	PSAT1-TLE4		C	T	0.93	0.182	2.80E-08	Yes		C	T2D	(25)	141 454
	rs12779790	10	12328010	CDC123-CAMK1D		G	A	0.20	0.104	6.75E-04	Yes		G	T2D	(25)	47 117
	rs1111875	10	94465559	HHEX-EXOC6		C	T	0.57	0.122	9.10E-15	No	rs5015480	C	T2D	(25)	47 117
	rs2334499	11	1696849	FAM99A-FAM99B		T	C	0.41	0.104	1.70E-02	Yes		T	T2D	(31)	2 251
	rs231362	11	2691471	KCNQ1	Intron	G	A	0.52	0.104	4.70E-13	Yes		G	T2D	(25)	141 454
	rs2237892	11	2849530	KCNQ1	Intron	C	T	0.94	0.131	2.70E-03	No	rs2283228	A	T2D	(25)	47 117
	rs5215	11	17405617	KCNJ11	Exon non-syn.	C	T	0.41	0.104	1.60E-05	No	rs1002226	C	T2D	(25)	47 117
Diabetes	rs1552224	11	72428172	ARAP1	Exon non-coding	A	C	0.88	0.122	2.90E-22	No	rs76550717	A	T2D	(25)	141 454

Risk Group	Original SNP	Chr	Position of original SNP (GRCh37)	Locus (Gene)	Functional Consequence	Alleles		EAF	European ancestry values as published		Present in ADVANCE?	Best ADVANCE Proxy	ADVANCE Effect Allele	Risk Factor	Ref	Nb. of Ind
						Effect	Other		β	p-value						
	rs1387153	11	92690661	FAT3-MTNR1B		T	C	0.28	0.030	3.96E-11	No	rs11020124	C	HbA1c	(26)	32 293
	rs10830963	11	92708710	MTNR1B	Intron	G	C	0.30	0.122	1.01E-06	Yes		G	T2D	(25)	47 117
	rs1153188	12	55098996	DCD-MUCL1		A	T	0.73	0.077	1.80E-07	Yes		A	T2D	(29)	62 301
	rs1531343	12	66171053	RPSAP52	Intron	C	G	0.10	0.182	7.20E-09	No	rs2884591	C	T2D	(25)	141 454
	rs7961581	12	71663102	TSPAN8-LGR5		C	T	0.25	0.086	1.82E-05	Yes		C	T2D	(25)	47 117
	rs7957197	12	121459344	OASL	Intron	T	A	0.85	0.131	4.90E-08	No	rs7131696	T	T2D	(25)	141 454
	rs7998202	13	113367449	ATP11AUN	Intron	G	A	0.14	0.030	5.24E-09	No	rs166864	A	HbA1c	(26)	34 724
	rs11634397	15	80432222	ZFAND-FAH		G	A	0.60	0.104	2.40E-09	Yes		G	T2D	(25)	141 454
	rs8042680	15	91521337	PRC1, PRC1-AS1	Intron, Intron	A	C	0.22	0.095	4.10E-10	Yes		A	T2D	(25)	141 454
	rs9939609	16	53820527	FTO	Intron	A	T	0.40	0.113	8.65E-08	Yes		A	T2D	(25)	47 117
	rs1046896	17	80685533	FN3KRP	3'UTR	T	C	0.31	0.040	1.57E-26	Yes		T	HbA1c	(26)	45 953
	rs855791	22	37462936	TMPRSS6	Exon non-syn.	A	G	0.42	0.030	2.74E-14	Yes		A	HbA1c	(26)	34 562
**Obesity**	rs3127553	1	49438005	AGBL4	Intron	A	G	0.63	0.023	1.60E-10	Yes		A	WC	(32)	231 815
	rs2815752	1	72812440	LOC105378797	Intron	A	G	0.61	0.130	1.17E-14	Yes		A	BMI	(33)	198 380
	rs7531118	1	72837239	LOC105378797	Intron	T	C	0.44	0.027	1.47E-14	Yes		T	WC	(32)	231 912
	rs1514175	1	74991644	FPGT-TNNI3K, TNNI3K	Intron, Intron	A	G	0.43	0.070	1.36E-09	Yes		A	BMI	(33)	227 900
	rs4130548	1	78463868	DNAJB4	Intron	T	C	0.65	0.022	3.40E-10	Yes		T	WC	(32)	232 042
	rs11165623	1	96893000	RWDD3-PTBP2		A	G	0.49	0.020	5.23E-09	Yes		A	WC	(32)	232 057
	rs1555543	1	96944797	RWDD3-PTBP2		C	A	0.59	0.060	7.65E-07	Yes		C	BMI	(33)	243 013
	rs984222	1	119503843	TBX15	Intron	G	C	0.37	0.034	8.69E-25	Yes		G	WHR	(34)	186 790
	rs1011731	1	172346548	DNM3	Intron	G	A	0.57	0.028	9.51E-18	Yes		G	WHR	(34)	169 112
	rs633715	1	177852580	BRINP2-SEC16B		T	C	0.80	0.043	3.30E-23	No	rs543874	C	WC	(32)	218 883
	rs543874	1	177889480	BRINP2-SEC16B		G	A	0.19	0.220	1.66E-13	Yes		G	BMI	(33)	179 414
	rs2820292	1	201784287	NAV1	Intron	A	G	0.45	0.019	2.37E-08	Yes		A	WC	(32)	231 899
	rs4846567	1	219750717	LYPLAL1-ZC3H11B		G	T	0.28	0.034	6.89E-21	Yes		G	WHR	(34)	168 987
	rs6429082	1	235600129	TBCE (near B3GALNT2)	Intron	C	T	0.50	0.026	2.59E-08	No	rs704710	A	WC	(35)	89 647
	rs2867125	2	622827	FAM150B-TMEM18		C	T	0.83	0.310	2.42E-22	Yes		C	BMI	(33)	197 806
	rs6755502	2	635721	TMEM18-FAM150B		C	T	0.17	0.051	2.03E-30	Yes	rs2867125	C	WC	(32)	231 849
	rs713586	2	25158008	ADCY3-DNAJC27		C	T	0.47	0.140	1.80E-07	Yes		C	BMI	(33)	230 748
	rs887912	2	59302877	FANCL-BCL11A		T	C	0.29	0.100	2.69E-06	Yes		T	BMI	(33)	242 807
	rs6545714	2	59307725	FANCL-BCL11A		A	G	0.61	0.022	1.94E-10	Yes		A	WC	(32)	232 046
	rs2890652	2	142959931	LRP1B-KYNU		C	T	0.18	0.090	2.38E-07	Yes		C	BMI	(33)	209 068
**Obesity**	rs10195252	2	165513091	GRB14-COBLL1		T	C	0.60	0.033	2.09E-24	Yes		T	WHR	(34)	179 568
	rs2176040	2	227092802	NYAP2-IRS1		A	G	0.37	0.034	1.41E-12	Yes		A	WC	(32)	231 926
	rs6784615	3	52506426	NISCH	Intron	T	C	0.94	0.043	3.84E-10	Yes		T	WHR	(34)	185 887
	rs6795735	3	64705365	ADAMTS9-AS2	Intron	C	T	0.41	0.025	9.79E-14	Yes		C	WHR	(34)	161 642
	rs3849570	3	81819040	GBE1	Intron	A	C	0.36	0.021	2.23E-08	No	rs11711331	A	WC	(32)	196 103

Risk Group	Original SNP	Chr	Position of original SNP (GRCh37)	Locus (Gene)	Functional Consequence	Alleles		EAF	European ancestry values as published		Present in ADVANCE?	Best ADVANCE Proxy	ADVANCE Effect Allele	Risk Factor	Ref	Nb. of Ind
						Effect	Other		β	p-value						
	rs2325036	3	85819412	CADM2	Intron	A	C	0.60	0.023	2.06E-11	Yes		A	WC	(32)	232 048
	rs13078807	3	85842341	CADM2	Intron	G	A	0.20	0.100	9.81E-08	No	rs9852859	C	BMI	(33)	237 404
	rs6440003	3	141094209	ZBTB38	Intron	A	G	0.44	0.022	2.88E-10	Yes		A	WC	(32)	231 985
	rs1516725	3	185824004	ETV5	Intron	T	C	0.13	0.031	1.73E-09	Yes		T	WC	(32)	230 610
	rs9816226	3	185834499	ETV5-DGKG		T	A	0.82	0.140	7.61E-14	Yes		T	BMI	(33)	196 221
	rs10938397	4	45182527	GNPDA2-GABRG1		A	G	0.56	0.032	6.10E-20	Yes		A	WC	(32)	231 679
	rs2112347	5	75015242	LOC441087, POC5	Intron, Upstr.2KB	T	G	0.63	0.033	2.40E-13	Yes		T	WC	(32)	232 028
	rs4836133	5	124330522	ZNF608-GRAMD3		A	C	0.48	0.070	7.04E-07	No	rs6864049	C	BMI	(33)	241 999
	rs6861681	5	173362458	CPEB4	Intron	A	G	0.34	0.022	1.91E-09	Yes		A	WHR	(34)	162 886
	rs1294421	6	6743149	LOC101928004	Intron	G	T	0.39	0.028	1.75E-17	Yes		G	WHR	(34)	179 343
	rs806794	6	26200677	HIST1H2BF	Downstream 500B	A	G	0.69	0.022	2.12E-09	Yes		A	WC	(32)	225 694
	rs206936	6	34302869	RPS10-NUDT3, NUDT3	Intron, Intron	G	A	0.21	0.060	2.81E-06	Yes		G	BMI	(33)	249 777
	rs16894959	6	34769765	UHRF1BP1	Exon syn.	T	C	0.84	0.026	3.44E-08	No	rs4472337	T	WC	(32)	230 687
	rs6905288	6	43758873	VEGFA-LOC105375070		A	G	0.56	0.036	5.88E-25	Yes		A	WHR	(34)	172 559
	rs987237	6	50796905	TFAP2B	Intron	G	A	0.18	0.130	5.97E-16	No	rs62405422	C	BMI	(33)	195 776
	rs943005	6	50829192	TFAP2B-PKHD1		C	T	0.18	0.039	7.22E-19	No	rs62405422	C	WC	(32)	232 080
	rs9400239	6	108977663	FOXO3	Intron	T	C	0.31	0.024	1.88E-11	Yes		T	WC	(32)	232 015
	rs9491696	6	127452639	RSPO3	Intron	G	C	0.52	0.042	1.84E-40	No	rs2489623	G	WHR	(34)	190 746
	rs2489623	6	127455821	RSPO3	Intron	A	C	0.48	0.019	3.41E-08	Yes		A	WC	(32)	231 857
	rs1055144	7	25871109	LOC100506236	Non coding	T	C	0.21	0.040	9.97E-25	Yes		T	WHR	(34)	190 781
	rs10968576	9	28414339	LINGO2	Intron	A	G	0.68	0.025	1.17E-11	Yes		A	WC	(32)	232 050
	rs6163	10	104596924	CYP17A1	Exon syn.	A	C	0.39	0.019	3.68E-08	Yes		A	WC	(32)	225 708
	rs7903146	10	114758349	TCF7L2	Intron	T	C	0.29	0.022	3.92E-09	Yes		T	WC	(32)	232 078
	rs4929949	11	8524973	STK33	Intron	C	T	0.52	0.060	7.57E-08	No	rs7949988	T	BMI	(33)	249 791
	rs10840100	11	8669437	TRIM66	Intron	A	G	0.36	0.020	5.42E-09	Yes		A	WC	(32)	232 029
	rs10767658	11	27672252	BDNF-AS (near BDNF)	Intron	C	G	0.31	0.031	3.29E-17	Yes		C	WC	(32)	225 754
	rs10767664	11	27725986	BDNF	Intron	A	T	0.78	0.190	5.53E-13	Yes		A	BMI	(33)	204 158
	rs2293576	11	47434986	SLC39A13	Exon syn.	A	G	0.33	0.022	9.36E-10	Yes		A	WC	(32)	226 024
**Obesity**	rs3817334	11	47566352	MTCH2	Intron	T	C	0.41	0.060	4.79E-11	No	rs7928842	C	BMI	(33)	191 943
	rs718314	12	26453283	LOC105369705	Intron	G	A	0.74	0.030	1.14E-17	Yes		G	WHR	(34)	184 670
	rs7138803	12	50247468	BCDIN3D-FAIM2		A	G	0.38	0.028	1.55E-15	Yes		A	WC	(32)	232 059
	rs1443512	12	54342684	HOXC13-HOXC12		A	C	0.24	0.031	6.38E-17	Yes		A	WHR	(34)	189 518
	rs4771122	13	28029896	MTIF3	Intron	G	A	0.24	0.090	1.20E-07	No	rs9512699	G	BMI	(33)	198 577
	rs12429545	13	54102206	LOC105370210-OLFM4		A	G	0.13	0.031	2.49E-09	Yes		A	WC	(32)	222 998
	rs10132280	14	25928179	STXBP6-NOVA1		A	C	0.32	0.022	2.25E-09	Yes		A	WC	(32)	231 985
	rs12885454	14	29736838	LOC102724934	Non coding	A	C	0.36	0.020	2.59E-08	Yes		A	WC	(32)	231 357
	rs10150332	14	79936964	NRXN3	Intron	C	T	0.21	0.130	2.03E-07	Yes		C	BMI	(33)	183 022

Risk Group	Original SNP	Chr	Position of original SNP (GRCh37)	Locus (Gene)	Functional Consequence	Alleles		EAF	European ancestry values as published		Present in ADVANCE?	Best ADVANCE Proxy	ADVANCE Effect Allele	Risk Factor	Ref	Nb. of Ind
						Effect	Other		β	p-value						
	rs7144011	14	79940383	NRXN3	Intron	C	T	0.22	0.033	9.40E-16	No	rs10150332	C	WC	(32)	232 007
	rs2241423	15	68043057	MAP2K5	Intron	G	A	0.78	0.130	1.15E-10	No	rs8028313	C	BMI	(33)	227 950
	rs4776970	15	68080886	MAP2K5	Intron	A	T	0.64	0.028	5.25E-09	Yes		A	WC	(32)	230 989
	rs2531992	16	4021734	ADCY9	Intron	A	G	0.15	0.028	3.03E-09	Yes		A	WC	(32)	232 034
	rs12444979	16	19933600	GPRC5B-GPR139		C	T	0.87	0.170	4.20E-11	Yes		C	BMI	(33)	239 715
	rs12446632	16	19935389	LOC105371116	Intron	C	T	0.14	0.036	5.21E-13	No	rs12444979	C	WC	(32)	226 701
	rs2650492	16	28347140	SBK1	3'UTR	C	A	0.30	0.026	2.46E-11	No	rs2726036	C	WC	(32)	231 921
	rs7498665	16	28883241	SH2B1	Exon non-syn.	T	C	0.60	0.034	1.43E-22	No	rs7359397	T	WC	(32)	230 193
	rs7359397	16	28885659	SH2B1	Downstream 500B	T	C	0.40	0.150	1.75E-10	Yes		T	BMI	(33)	204 309
	rs1549293	16	31141993	KAT8 (near PRSS8)	Intron	T	C	0.38	0.020	7.29E-09	Yes		T	WC	(32)	232 022
	rs1558902	16	53786615	FTO	Intron	A	T	0.42	0.074	3.70E-101	No	rs9939609	A	WC	(32)	230 183
	rs7239883	18	40147671	LINC00907	Intron	A	G	0.60	0.021	2.30E-09	Yes		A	WC	(32)	231 731
	rs6567160	18	57829135	PMAIP1-MC4R		T	C	0.77	0.048	2.61E-33	No	rs571312	A	WC	(32)	231 894
	rs571312	18	57839769	CCBE1-CDH20		A	C	0.24	0.230	1.82E-22	Yes		A	BMI	(33)	203 600
	rs29941	19	34309532	KCTD15-LSM14A		G	A	0.67	0.060	1.31E-09	Yes		G	BMI	(33)	192 872
	rs3810291	19	47569003	ZC3H4	3'UTR	A	G	0.67	0.026	1.67E-10	Yes		A	WC	(32)	210 501
	rs16996700	20	50981945	LOC105372666	Intron	T	C	0.73	0.023	1.52E-09	Yes		T	WC	(32)	231 903
	rs4823006	22	29451671	ZNRF3	3'UTR	A	G	0.57	0.023	1.10E-11	Yes		A	WHR	(34)	170 997
**Albuminuria**	rs11579312	1	30656572	LOC105378617	Intron	T	C	0.69	0.110	9.70E-06	Yes		T	MA	(36)	54 116
	rs914615	1	155175892	THBS3	Intron	A	G	0.47	0.030	7.40E-06	Yes		G	UACR	(36)	44 877
	rs13427836	2	129027961	HS6ST1	Intron	T	C	0.14	0.199	6.10E-06	Yes		T	UACR	(36)	5 509
	rs17346504	2	137923761	THSD7B	Intron	T	C	0.12	0.050	7.20E-06	Yes		T	UACR	(36)	53 401
	rs16827742	2	150901005	MMADHC-RND3		A	G	0.06	0.300	3.10E-06	No	rs114375510	C	MA	(36)	35 962
	rs11678190	2	187560308	FAM171B	Intron	A	C	0.70	0.100	5.10E-06	Yes		C	MA	(36)	54 107
**Albuminuria**	rs11678190	2	187560308	FAM171B	Intron	A	C	0.70	0.035	5.10E-06	Yes		C	UACR	(36)	54 107
	rs13079877	3	2130400	CNTN4-CNTN6		A	G	0.45	0.148	5.60E-06	No	rs7633224	T	UACR	(36)	5 825
	rs1077216	3	46892161	MYL3-PRSS42		T	C	0.07	0.200	5.20E-06	Yes		T	MA	(36)	45 096
	rs7634770	3	66931027	LOC105377144	Intron	A	C	0.70	0.142	2.70E-06	No	rs34401437	T	UACR	(36)	5 825
	rs13160548	5	38778850	OSMR-AS1	Intron	T	C	0.69	0.100	8.20E-06	Yes		C	MA	(36)	53 130
	rs12719264	5	119183940	LOC105379144	Intron	A	G	0.30	0.110	6.20E-06	Yes		G	MA	(36)	54 115
	rs17738155	6	51156076	TFAP2B-PKHD1		T	C	0.92	0.241	5.90E-06	Yes		C	UACR	(36)	5 825
	rs2110904	6	107594771	PDSS2	Intron	T	C	0.65	0.100	8.90E-06	Yes		T	MA	(36)	54 116
	rs4722909	7	29514931	CHN2	Intron	A	G	0.60	0.134	3.20E-06	Yes		G	UACR	(36)	5 823
	rs17301329	8	54858981	RGS20	Intron	A	T	0.29	0.042	5.60E-07	Yes		A	UACR	(36)	54 450
	rs7851726	9	97503985	C9orf3	Intron	T	C	0.42	0.027	5.20E-06	Yes		T	UACR	(36)	54 450
	rs1109861	10	11246269	CELF2	Intron	A	C	0.55	0.030	1.90E-06	Yes		C	UACR	(36)	54 442
	rs17343073	10	16937659	CUBN	Intron	A	T	0.90	0.071	4.00E-09	No	rs79801018	C	UACR	(36)	54 449

Risk Group	Original SNP	Chr	Position of original SNP (GRCh37)	Locus (Gene)	Functional Consequence	Alleles		EAF	European ancestry values as published		Present in ADVANCE?	Best ADVANCE Proxy	ADVANCE Effect Allele	Risk Factor	Ref	Nb. of Ind
						Effect	Other		β	p-value						
	rs6602163	10	16966766	CUBN	Intron	A	G	0.84	0.056	1.20E-09	Yes		G	UACR	(36)	54 450
	rs12764441	10	72691651	PCBD1-UNC5B		T	C	0.48	0.100	3.50E-06	Yes		C	MA	(36)	54 116
	rs3740393	10	104636655	BORCS7-ASMT, AS3MT	Intron, Intron	C	G	0.21	0.130	6.10E-06	Yes		C	MA	(36)	54 048
	rs7922045	10	123001732	WDR11-FGFR2		T	C	0.26	0.165	5.70E-07	Yes		T	UACR	(36)	5 824
	rs729014	10	123002806	WDR11-FGFR2		T	C	0.15	0.202	2.40E-06	Yes		T	UACR	(36)	5 825
	rs10899033	11	74393342	POLD3-CHRDL2		C	G	0.72	0.110	9.30E-06	No	rs7111585	T	MA	(36)	54 116
	rs649529	11	88006821	CTSC-RAB38		T	G	0.43	0.147	9.30E-06	No	rs605042	A	UACR	(36)	5 825
	rs2303658	12	20278430	LOC105369684	Intron	A	G	0.34	0.030	9.50E-06	Yes		A	UACR	(36)	54 442
	rs7145202	14	23092105	ABHD4-OXA1L		T	C	0.62	0.100	3.70E-06	Yes		T	MA	(36)	54 106
	rs1728897	15	55301370	UNC13C-RSL24D1		T	C	0.54	0.028	4.10E-06	Yes		C	UACR	(36)	54 433
	rs1528472	15	55321128	UNC13C-RSL24D1		A	C	0.48	0.032	5.40E-07	Yes		C	UACR	(36)	54 445
	rs231227	19	36268067	ARHGAP33	5'UTR	A	G	0.38	0.033	4.90E-06	Yes		A	UACR	(36)	44 877
	rs6513791	20	41058122	PTPRT	Intron	T	C	0.18	0.120	4.40E-06	Yes		T	MA	(36)	54 115
	rs2828785	21	25437505	No gene within 1 Mb		T	C	0.27	0.038	7.90E-06	Yes		C	UACR	(36)	54 450
**Glomerular Filtration Rate**	rs1800615	1	15832281	CASP9	Intron	T	C	0.30	0.006	1.90E-09	Yes		T	eGFR	(37)	133 413
	rs12136063	1	110014170	SYPL2	Intron	A	G	0.70	0.005	4.71E-08	Yes		G	eGFR	(37)	175 426
	rs267734	1	150951447	CERS2-ANXA9		T	C	0.79	0.008	4.01E-13	Yes		T	eGFR	(37)	133 413
	rs3850625	1	201016296	CACNA1S	Exon non-syn.	A	G	0.12	0.008	6.82E-11	Yes		G	eGFR	(37)	153 107
	rs2802729	1	243501763	SDCCAG8	Intron	A	C	0.43	0.005	2.20E-08	Yes		A	eGFR	(37)	174 808
	rs807601	2	15793014	DDX1-MYCN		T	G	0.34	0.006	6.60E-12	Yes		G	eGFR	(37)	133 413
**Glomerular Filtration Rate**	rs1260326	2	27741237	GCKR	Exon non-syn.	T	C	0.42	0.007	3.38E-14	No	rs780094	C	eGFR	(37)	133 413
	rs6546838	2	73679280	ALMS1	Exon non-syn.	A	G	0.76	0.009	7.72E-20	Yes		A	eGFR	(37)	133 413
	rs4667594	2	170008506	LRP2	Intron	A	T	0.53	0.004	3.52E-08	Yes		A	eGFR	(37)	175 337
	rs2712184	2	217682779	LOC101928278	Intron	A	C	0.58	0.005	1.33E-10	Yes		A	eGFR	(37)	153 854
	rs6795744	3	13906850	WNT7A	Intron	A	G	0.15	0.006	3.33E-08	Yes		G	eGFR	(37)	175 490
	rs347685	3	141807137	TFDP2	Intron	A	C	0.72	0.009	3.00E-11	Yes		A	eGFR	(38)	67 093
	rs9682041	3	170120054	SKIL	Intron	T	C	0.87	0.007	2.58E-08	No	rs6767869	C	eGFR	(37)	150 911
	rs10513801	3	185824004	ETV5	Intron	T	G	0.87	0.007	1.03E-09	No	rs1516725	T	eGFR	(37)	154 774
	rs17319721	4	77368847	SHROOM3	Intron	A	G	0.43	0.011	1.32E-37	Yes		A	eGFR	(37)	133 413
	rs228611	4	103561709	MANBA (near NFKB1)	Intron	A	G	0.47	0.006	3.58E-12	Yes		A	eGFR	(37)	175 445
	rs11959928	5	39393733	DAB2	Intron	A	T	0.44	0.009	1.40E-17	No	rs10062079	A	eGFR	(38)	67 093
	rs7759001	6	27341409	ZNF204P, ZNF391	Intron, Upstr.2KB	A	G	0.76	0.005	1.75E-08	Yes		A	eGFR	(37)	175 483
	rs9472135	6	43809802	LOC107986598	Intron	T	C	0.71	0.008	3.34E-15	Yes		T	eGFR	(37)	133 413
	rs316009	6	160675764	SLC22A2	Intron	T	C	0.10	0.013	4.38E-19	Yes		C	eGFR	(37)	133 413
	rs3127573	6	160681393	SLC22A2	Upstr. 2KB	G	A	0.13	1.100	6.50E-10	Yes		G	CREAT	(39)	23 812
	rs3750082	7	32927727	KBTBD2	Intron	A	T	0.33	0.005	3.22E-08	No	rs970673	A	eGFR	(37)	168 494
	rs848490	7	77555005	PHTF2	Intron	C	G	0.73	0.007	7.80E-13	Yes		G	eGFR	(37)	133 413

Risk Group	Original SNP	Chr	Position of original SNP (GRCh37)	Locus (Gene)	Functional Consequence	Alleles		EAF	European ancestry values as published		Present in ADVANCE?	Best ADVANCE Proxy	ADVANCE Effect Allele	Risk Factor	Ref	Nb. of Ind
						Effect	Other		β	p-value						
	rs7805747	7	151406220	PRKAG2	Intron	A	G	0.25	0.013	7.96E-29	No	rs17173238	G	eGFR	(37)	133 413
	rs1731274	8	23766319	STC1-ADAM28		G	A	0.45	0.017	4.60E-08	Yes		G	eGFR	(40)	90 075
	rs4744712	9	71434707	PIP5K1B	Intron	A	C	0.40	0.007	4.29E-15	Yes		A	eGFR	(37)	133 413
	rs1044261	10	997740	IDI2	Stop-Gain	T	C	0.08	0.011	1.21E-11	No	rs149288300	G	eGFR	(37)	133 413
	rs10994860	10	52645424	A1CF	5'UTR	T	C	0.19	0.008	1.07E-12	Yes		C	eGFR	(37)	154 644
	rs163160	11	2789955	KCNQ1	Intron	A	G	0.82	0.007	2.26E-09	Yes		G	eGFR	(37)	154 684
	rs963837	11	30749090	LOC101928316	Intron	T	C	0.54	0.008	5.69E-18	Yes		T	eGFR	(37)	133 413
	rs4014195	11	65506822	RNASEH2C-AP5B1		C	G	0.64	0.006	1.10E-11	Yes		G	eGFR	(37)	175 400
	rs10774021	12	349298	SLC6A13	Intron	T	C	0.65	0.006	4.77E-12	Yes		T	eGFR	(37)	133 413
	rs10491967	12	3368093	TSPAN9	Intron	A	G	0.10	0.010	5.18E-14	Yes		A	eGFR	(37)	175 670
	rs7956634	12	15321194	RERG	Intron	T	C	0.81	0.007	7.17E-12	Yes		T	eGFR	(37)	175 448
	rs1106766	12	57738600	R3HDM2 (near INHBC)	Intron	T	C	0.22	0.006	2.41E-09	No	rs79395356	T	eGFR	(37)	154 665
	rs653178	12	111865049	ATXN2	Intron	C	T	0.51	0.003	3.50E-11	No	rs7310615	C	eGFR	(38)	67 093
	rs626277	13	72347696	DACH1	Intron	A	C	0.60	0.009	2.50E-11	Yes		A	eGFR	(38)	67 093
	rs476633	15	41392134	INO80	Intron	C	G	0.57	0.005	8.90E-09	Yes		G	eGFR	(37)	133 413
	rs2453533	15	45641225	SLC28A2-GATM		A	C	0.62	0.013	4.60E-22	Yes		A	eGFR	(38)	67 093
**Glomerular Filtration Rate**	rs491567	15	53946593	WDR72	Intron	A	C	0.78	0.008	2.86E-15	Yes		A	eGFR	(37)	133 413
	rs1394125	15	76158983	UBE2Q2	Intron	A	G	0.35	0.007	5.47E-14	Yes		A	eGFR	(37)	133 413
	rs4293393	16	20364588	UMOD	Intron	T	C	0.80	1.930	1.30E-23	Yes		T	CREAT	(41)	24 635
	rs13329952	16	20366507	UMOD	Intron	T	C	0.81	0.016	9.47E-43	No	rs4293393	T	eGFR	(37)	133 413
	rs164748	16	89708292	DPEP1	Downstream 500B	C	G	0.53	0.005	1.95E-08	Yes		G	eGFR	(37)	154 497
	rs11657044	17	59450105	BCAS3	Intron	T	C	0.19	0.012	7.89E-22	Yes		T	eGFR	(37)	133 413
	rs8068318	17	59483766	TBX2	Intron	G	A	0.27	0.800	3.40E-10	Yes		G	CREAT	(39)	23 812
	rs12460876	19	33356891	SLC7A9	Intron	T	C	0.60	0.007	1.86E-13	Yes		T	eGFR	(37)	133 413
	rs11666497	19	38464262	SIPA1L3	Intron	T	C	0.18	0.006	4.25E-08	Yes		T	eGFR	(37)	168 911
	rs6088580	20	33285053	PIGU-TP53INP2		C	G	0.47	0.005	1.79E-09	Yes		C	eGFR	(37)	167 365
	rs17216707	20	52742306	BCAS1-CYP24A1		T	C	0.79	0.008	8.83E-15	No	rs8121940	C	eGFR	(37)	173 627
**Blood Pressure**	rs17367504	1	11887303	MTHFR	Intron	G	A	0.15	0.725	8.70E-22	No	rs7537765	A	HTN	(42)	125 372
	rs17367504	1	11887303	MTHFR	Intron	G	A	0.15	0.903	8.70E-22	No	rs7537765	A	SBP	(42)	125 372
	rs848309	1	16308447	ZBTB17-C1orf64		T	C	0.57	0.347	7.07E-12	Yes		T	SBP	(43)	287 217
	rs4360494	1	38455891	SF3A3	Upstr. 2KB	C	G	0.55	0.278	3.70E-16	Yes		C	PP	(44)	282 851
	rs112557609	1	56576924	LOC105378741	Intron	A	G	0.35	0.227	6.80E-12	Yes		A	PP	(44)	325 952
	rs3889199	1	59653742	JUN-FGGY		A	G	0.71	0.351	1.80E-24	Yes		A	PP	(44)	329 486
	rs2932538	1	113216543	MOV10	Upstream 2KB	G	A	0.75	0.388	1.20E-09	Yes		G	SBP	(42)	194 667
	rs2289081	2	20881840	LDAH	Downstream 500B	C	G	0.36	0.223	5.50E-12	Yes		C	PP	(44)	329 140
	rs11690961	2	46363336	PRKCE	Intron	A	C	0.88	0.340	3.90E-12	Yes		A	PP	(44)	327 847
	rs74181299	2	65283972	CEP68	Intron	T	C	0.62	0.230	9.60E-13	Yes		T	PP	(44)	324 224

Risk Group	Original SNP	Chr	Position of original SNP (GRCh37)	Locus (Gene)	Functional Consequence	Alleles		EAF	European ancestry values as published		Present in ADVANCE?	Best ADVANCE Proxy	ADVANCE Effect Allele	Risk Factor	Ref	Nb. of Ind
						Effect	Other		β	p-value						
	rs11689667	2	85491365	TCF7L1	Intron	T	C	0.54	0.176	1.70E-08	Yes		T	PP	(44)	330 634
	rs1250259	2	216300185	FN1	Exon non-syn.	A	T	0.74	0.314	8.70E-19	No	rs1250258	T	PP	(44)	325 485
	rs13082711	3	27537909	SLC4A7-EOMES		T	C	0.78	0.315	1.50E-06	Yes		C	SBP	(42)	198 112
	rs3774372	3	41877414	ULK4	Exon non-syn.	T	C	0.83	0.067	3.90E-01	Yes		C	SBP	(42)	162 083
	rs419076	3	169113110	MECOM	Intron	T	C	0.47	0.325	3.10E-04	No	rs2421647	C	HTN	(42)	193 725
	rs419076	3	169113110	MECOM	Intron	T	C	0.47	0.409	1.80E-13	No	rs2421647	C	SBP	(42)	193 725
	rs871606	4	54799245	LNX1-CHIC2		T	C	0.85	0.429	1.32E-08	Yes		T	PP	(45)	115 525
	rs1458038	4	81164723	PRDM8-FGF5		T	C	0.29	0.706	1.50E-23	Yes		T	SBP	(42)	139 686
	rs13107325	4	103112470	SLC39A8	Exon non-syn.	T	C	0.05	0.981	3.30E-14	No	rs6855246	A	SBP	(42)	150 915
	rs78049276	4	148427503	EDNRA	Intron	C	A	0.13	0.267	1.00E-08	Yes		C	PP	(44)	321 315
	rs146853253	4	156391307	MAP9-GUCY1A3		Del	A	0.16	0.316	7.00E-14	No	rs17033041	G	PP	(44)	322 302
	rs13139571	4	156645513	GUCY1A3	Intron	C	A	0.76	0.321	1.20E-06	Yes		C	SBP	(42)	185 421
**Blood Pressure**	rs1566497	4	169717148	PALLD	Intron	A	C	0.42	0.236	1.90E-13	Yes		A	PP	(44)	320 948
	rs17059668	4	174584663	HAND2-AS1-FBXO8		C	G	0.92	0.332	2.80E-08	Yes		C	PP	(44)	313 277
	rs1173771	5	32819073	NPR3-TARS		G	A	0.60	0.383	3.20E-10	No	rs9292468	C	HTN	(42)	131 276
	rs10057188	5	77837789	LHFPL2	Intron	A	G	0.46	0.205	6.70E-11	Yes		A	PP	(44)	325 985
	rs31864	5	158227898	EBF1	Intron	A	G	0.55	0.206	6.00E-11	No	rs12332693	A	PP	(44)	326 557
	rs1799945	6	26091179	HFE	Exon non-syn.	G	C	0.14	0.627	7.70E-12	Yes		G	SBP	(42)	148 597
	rs805303	6	31724345	BAG6	Intron	G	A	0.61	0.376	1.50E-11	Yes		G	SBP	(42)	201 745
	rs185819	6	32050067	TNXB	Exon non-syn.	T	C	0.51	0.365	1.04E-17	Yes		T	SBP	(43)	364 144
	rs11154027	6	121781390	GJA1-HSF2		T	C	0.47	0.207	1.10E-10	Yes		T	PP	(44)	316 708
	rs36083386	6	152397913	ESR1	Intron	C	Del	0.11	0.439	1.50E-18	Yes		AC	PP	(44)	323 303
	rs449789	6	159699125	FNDC1-LOC105378084		C	G	0.14	0.359	2.40E-15	Yes		C	PP	(44)	325 584
	rs1322639	6	169587103	SMOC2-THBS2		A	G	0.78	0.316	4.80E-17	Yes		A	PP	(44)	319 866
	rs76206723	7	40410924	SUGCT	Intron	A	G	0.10	0.346	7.40E-12	No	rs77410344	C	PP	(44)	328 162
	rs17477177	7	106414069	CCDC71L-PIK3CG		C	T	0.28	0.418	2.27E-13	No	rs2392929	G	PP	(45)	112 996
	rs6557876	8	25904388	EBF2	Intron	C	T	0.25	0.371	2.85E-14	No	rs9314318	A	SBP	(43)	369 457
	rs35783704	8	105978368	LRP12-ZFPM2		G	A	0.11	0.414	7.08E-09	No	rs11774829	A	SBP	(43)	349 452
	rs2071518	8	120435812	NOV	3'UTR	T	C	0.17	0.312	3.66E-09	Yes		T	PP	(45)	119 056
	rs4454254	8	141060027	TRAPPC9	Intron	A	G	0.63	0.261	5.10E-16	Yes		A	PP	(44)	330 022
	rs72765298	9	127961103	SCAI	Intron	T	C	0.87	0.374	2.70E-14	No	rs869248	A	PP	(44)	316 271
	rs4373814	10	18419972	SLC39A12-CACNB2		G	C	0.55	0.373	4.80E-11	Yes		C	SBP	(42)	188 387
	rs1813353	10	18707448	CACNB2	Intron	T	C	0.68	0.569	2.60E-12	Yes		T	SBP	(42)	101 841
	rs9337951	10	30317073	KIAA1462	Exon syn.	A	G	0.34	0.280	2.50E-15	Yes		A	PP	(44)	299 646
	rs10826995	10	32082658	ARHGAP12-ZEB1		T	C	0.71	0.212	1.10E-09	Yes		T	PP	(44)	327 373
	rs4590817	10	63467553	C10orf107	Intron	G	C	0.84	0.646	4.00E-12	Yes		G	SBP	(42)	111 048
	rs932764	10	95895940	PLCE1	Intron	G	A	0.44	0.484	7.10E-16	Yes		G	SBP	(42)	160 885

Risk Group	Original SNP	Chr	Position of original SNP (GRCh37)	Locus (Gene)	Functional Consequence	Alleles		EAF	European ancestry values as published		Present in ADVANCE?	Best ADVANCE Proxy	ADVANCE Effect Allele	Risk Factor	Ref	Nb. of Ind
						Effect	Other		β	p-value						
	rs11191548	10	104719096	CNNM2 (near NT5C2)	Non coding	T	C	0.91	1.095	6.90E-26	No	rs12413409	G	SBP	(42)	161 709
	rs7129220	11	10350538	ADM-AMPD3		G	A	0.89	0.619	3.00E-12	Yes		A	SBP	(42)	182 871
	rs381815	11	16902268	PLEKHA7	Intron	T	C	0.26	0.575	5.30E-11	Yes		T	SBP	(42)	97 159
	rs11442819	11	45213509	PRDM11	Intron	T	Del	0.11	0.279	7.10E-09	No	rs4755950	C	PP	(44)	326 483
	rs2289125	11	89224477	NOX4	5'UTR	A	C	0.21	0.377	9.10E-22	No	rs2289124	A	PP	(44)	307 682
	rs633185	11	100593538	ARHGAP42	Intron	G	C	0.28	0.565	1.20E-17	Yes		C	SBP	(42)	160 461
	rs8258	11	117283676	CEP164	3'UTR	T	C	0.38	0.236	2.90E-13	Yes		T	PP	(44)	327 038
**Blood pressure**	rs11222084	11	130273230	ZBTB44-DT (near ADAMTS8)	Non coding	T	A	0.38	0.337	1.90E-11	Yes		T	PP	(45)	108 095
	rs10770612	12	20230639	LOC100506393	Intron	A	G	0.80	0.313	7.00E-15	Yes		A	PP	(44)	311 586
	rs73099903	12	53440779	TNS2, LOC283335	5'UTR, Intron	C	T	0.07	0.515	1.95E-10	Yes		C	SBP	(43)	343 318
	rs7312464	12	66374247	HMGA2-LLPH		G	A	0.52	0.210	5.00E-10	Yes		G	PP	(44)	289 978
	rs17249754	12	90008959	ATP2B1	Intron	G	A	0.84	0.928	1.80E-18	No	rs2681472	G	SBP	(42)	96 425
	rs139236208	12	94872851	LOC102724960	Intron	A	G	0.10	0.363	1.60E-10	No	rs140802080	A	PP	(44)	291 244
	rs3184504	12	111865049	SH2B3	Exon non-syn		C	0.47	0.523	2.60E-06	No	rs7310615	C	HTN	(42)	120 574
	rs3184504	12	111865049	SH2B3	Exon non-syn.	T	C	0.47	0.598	3.80E-18	No	rs7310615	C	SBP	(42)	120 574
	rs10850411	12	115387796	TBX3-MED13L		T	C	0.7	0.354	5.40E-08	Yes		T	SBP	(42)	161 142
	rs12434998	14	94455554	LOC107984663	Intron	C	T	0.37	0.189	2.00E-08	Yes		C	PP	(44)	315 683
	rs9323988	14	98594361	LOC105370655	Intron	T	C	0.63	0.212	4.10E-11	No	rs8018617	A	PP	(44)	327 551
	rs1378942	15	75077367	CSK	Intron	C	A	0.35	0.613	5.70E-23	Yes		C	SBP	(42)	163 124
	rs56249585	16	65286187	CDH11-LINC00922		T	C	0.53	0.183	9.00E-09	No	rs9931601	C	PP	(44)	318 082
	rs7500448	16	83045790	CDH13	Intron	A	G	0.75	0.329	1.10E-19	Yes		A	PP	(44)	321 958
	rs7226020	17	6473353	PITPNM3-KIAA0753		T	C	0.56	0.256	2.30E-14	No	rs930526	C	PP	(44)	303 389
	rs62080325	17	42060631	PYY	Intron	A	G	0.66	0.186	4.00E-08	Yes		A	PP	(44)	315 689
	rs17608766	17	45013271	GOSR2	Intron	T	C	0.86	0.556	1.10E-10	Yes		C	SBP	(42)	151 810
	rs12940887	17	47402807	ZNF652	Intron	T	C	0.38	0.362	1.80E-10	Yes		T	SBP	(42)	188 184
	rs57927100	17	75317932	SEPT9	Intron	C	G	0.26	0.310	4.04E-10	No	rs77946446	T	SBP	(43)	347 188
	rs7236548	18	43095231	SLC14A2	Intron	A	C	0.18	0.352	2.00E-18	No	rs7245140	C	PP	(44)	330 075
	rs2116941	19	10372360	S1PR2	3'UTR	C	A	0.81	0.220	3.00E-08	No	rs200688233	G	PP	(44)	321 960
	rs2206815	20	10658882	JAG1-C20orf187		A	C	0.50	0.326	5.00E-25	No	rs6040076	G	PP	(44)	324 088
	rs1327235	20	10969030	C20orf187	Intron	G	A	0.46	0.340	1.90E-08	Yes		G	SBP	(42)	158 478
	rs6081613	20	19461866	SLC24A3	Intron	A	G	0.28	0.263	1.60E-13	No	rs3838376	C	PP	(44)	315 546
	rs6015450	20	57713314	ZNF831	Intron	A	G	0.28	0.727	3.90E-23	No	rs8116295	A	HTN	(42)	159 190
	rs6015450	20	57713314	ZNF831	Intron	G	A	0.12	0.896	3.90E-23	No	rs8116295	A	SBP	(42)	159 190
	rs12628032	22	19967980	ARVCF	Intron	T	C	0.30	0.240	5.50E-12	Yes		T	PP	(44)	267 722
	rs73161324	22	42038786	XRCC6	Intron	T	C	0.05	0.496	2.80E-11	Yes		T	PP	(44)	310 292
	rs12037222	1	40060025	PABPC4-HEYL		A	G	0.24	0.045	6.40E-11	No	rs76841360	A	CRP	(46)	82 725

Risk Group	Original SNP	Chr	Position of original SNP (GRCh37)	Locus (Gene)	Functional Consequence	Alleles		EAF	European ancestry values as published		Present in ADVANCE?	Best ADVANCE Proxy	ADVANCE Effect Allele	Risk Factor	Ref	Nb. of Ind
						Effect	Other		β	p-value						
Biomarkers Levels	rs4420065	1	66161461	LEPR-PDE4B		C	T	0.61	0.090	3.50E-62	Yes		C	CRP	(46)	82 725
	rs4129267	1	154426264	IL6R	Intron	C	T	0.60	0.079	2.10E-48	Yes		C	CRP	(46)	82 725
	rs2794520	1	159678816	APCS-CRP		C	T	0.66	0.160	2.00E-186	Yes		C	CRP	(46)	82 725
Biomarkers Levels	rs12239046	1	247601595	NLRP3	Intron	C	T	0.61	0.047	1.20E-15	Yes		C	CRP	(46)	82 725
	rs1260326	2	27741237	GCKR	Exon non-syn.	T	C	0.41	0.072	4.60E-40	No	rs780094	T	CRP	(46)	82 725
	rs6734238	2	113841030	IL1F10-IL1RN		G	A	0.42	0.050	1.80E-17	Yes		G	CRP	(46)	82 725
	rs511154	3	138104635	MSL2-PCCB		A	G	0.23	0.045	5.94E-10	No	rs34905952	A	Fibri	(47)	21 901
	rs1800789	4	155482743	FGB	Upstream 2KB	A	G	0.20	0.087	1.75E-30	Yes		A	Fibri	(47)	22 096
	rs2522056	5	131801726	C5orf56 (near IRF1)	Intron	G	A	0.20	0.063	1.31E-15	Yes		G	Fibri	(47)	22 096
	rs4705952	5	131839618	IRF1-IL5		G	A	0.20	0.042	1.30E-08	Yes		G	CRP	(46)	82 725
	rs6901250	6	117114025	GPRC6A	Exon syn.	G	A	0.30	0.035	4.80E-08	Yes		A	CRP	(46)	82 725
	rs13233571	7	72982874	BCL7B	Intron	C	T	0.86	0.054	3.60E-09	No	rs17145738	C	CRP	(46)	82 725
	rs9987289	8	9181395	LOC157273	Intron	A	G	0.10	0.069	3.40E-13	No	rs2169387	A	CRP	(46)	82 725
	rs10745954	12	103483094	C12orf42	Intron	A	G	0.50	0.039	1.60E-11	Yes		A	CRP	(46)	82 725
	rs1183910	12	121416988	HNF1A	Intron	G	A	0.67	0.149	2.10E-124	No	rs2244608	G	CRP	(46)	82 725
	rs340029	15	60894965	RORA, RORA-AS1	Intron, Intron	T	C	0.62	0.032	4.10E-09	Yes		T	CRP	(46)	82 725
	rs2847281	18	12821593	PTPN2	Intron	A	G	0.80	0.031	2.20E-08	Yes		A	CRP	(46)	82 725
	rs4420638	19	45422946	APOC1	Downstream 500B	A	G	0.80	0.236	8.80E-139	Yes		A	CRP	(46)	82 725
Lipids	rs12027135	1	25775733	TMEM57	Intron	A	T	0.45	1.220	4.00E-11	Yes		A	TC	(48)	100 184
	rs4660293	1	40028180	PABPC4	Intron	G	A	0.23	0.480	4.00E-10	Yes		G	HDL	(48)	99 855
	rs2479409	1	55504650	PCSK9	Upstream 2KB	G	A	0.30	2.010	2.00E-28	Yes		G	LDL	(48)	95 435
	rs2131925	1	63025942	DOCK7	Intron	T	G	0.68	4.940	9.00E-43	Yes		T	TG	(48)	96 598
	rs7515577	1	93014343	EVI5	Intron	A	C	0.79	1.180	3.00E-08	No	rs145341911	A	TC	(48)	100 165
	rs629301	1	109818530	CELSR2	3'UTR	T	G	0.78	5.650	1.00E-170	No	rs646776	T	LDL	(48)	95 454
	rs1689800	1	182168885	ZNF648	Intron	G	A	0.35	0.470	3.00E-10	Yes		G	HDL	(48)	99 900
	rs2642442	1	220973563	MARC1	Intron	T	C	0.68	1.390	6.00E-13	Yes		T	TC	(48)	100 098
	rs4846914	1	230294715	GALNT2	Intron	G	A	0.40	0.610	4.00E-21	No	rs4846913	C	HDL	(48)	99 881
	rs514230	1	234858597	IRF2BP2-TOMM20		T	A	0.48	1.360	5.00E-14	Yes		T	TC	(48)	100 184
	rs1367117	2	21263900	APOB	Exon non-syn.	A	G	0.30	4.050	4.00E-114	Yes		A	LDL	(48)	95 446
	rs4299376	2	44072576	ABCG8	Intron	G	T	0.30	2.750	2.00E-47	Yes		G	LDL	(48)	91 285
	rs7570971	2	135837906	RAB3GAP1	Intron	A	C	0.34	1.250	2.00E-08	Yes		A	TC	(48)	95 242
	rs2972146	2	227092802	NYAP2-IRS1		T	G	0.63	0.460	3.00E-09	No	rs2176040	T	HDL	(48)	96 875
	rs2290159	3	12628920	RAF1	Intron	G	C	7.80	1.420	4.00E-09	Yes		G	TC	(48)	99 434
	rs645040	3	138104635	MSL2-PCCB		T	G	0.78	2.220	3.00E-08	No	rs34905952	G	TG	(48)	96 597
	rs442177	4	88030261	AFF1	Intron	T	G	0.59	2.250	9.00E-12	Yes		T	TG	(48)	96 598
	rs6450176	5	53298025	ARL15	Intron	A	G	0.26	0.490	5.00E-08	Yes		A	HDL	(48)	99 900
Lipids	rs9686661	5	55861786	C5orf67	Intron	T	C	0.20	2.570	1.00E-10	Yes		T	TG	(48)	95 848

Risk Group	Original SNP	Chr	Position of original SNP (GRCh37)	Locus (Gene)	Functional Consequence	Alleles		EAF	European ancestry values as published		Present in ADVANCE?	Best ADVANCE Proxy	ADVANCE Effect Allele	Risk Factor	Ref	Nb. of Ind
						Effect	Other		β	p-value						
	rs12916	5	74656539	HMGCR	3'UTR	C	T	0.39	2.840	9.00E-47	Yes		C	TC	(48)	100 184
	rs6882076	5	156394441	TIMD4	Upstream 2KB	C	T	0.65	1.980	7.00E-28	No	rs9715911	A	TC	(48)	100 184
	rs3757354	6	16127407	MYLIP	Upstream 2KB	C	T	0.78	1.430	1.00E-11	Yes		C	LDL	(48)	91 293
	rs3177928	6	32412435	HLA-DRA	3'UTR	A	G	0.16	2.310	4.00E-19	Yes		A	TC	(48)	100 151
	rs2814944	6	34769765	SPDEF-C6orf106		A	G	0.16	0.490	4.00E-09	No	rs4472337	T	HDL	(48)	99 811
	rs9488822	6	116312893	FRK	Intron	A	T	0.65	1.180	2.00E-10	Yes		A	TC	(48)	100 184
	rs605066	6	139835423	CITED2-LOC105378023		C	T	0.42	0.390	3.00E-08	No	rs71562509	G	HDL	(48)	99 900
	rs1564348	6	160578860	SLC22A1	Intron	T	C	0.83	0.560	2.00E-17	Yes		T	LDL	(48)	95 439
	rs12670798	7	21607352	DNAH11	Intron	C	T	0.23	1.430	9.00E-10	Yes		C	TC	(48)	100 184
	rs2072183	7	44582331	NPC1L1	Exon syn.	C	G	0.25	2.010	3.00E-11	No	rs2073547	G	TC	(48)	97 063
	rs17145738	7	72982874	TBL2	Downstr.500B	C	T	0.88	9.320	6.00E-58	Yes		C	TG	(48)	96 598
	rs4731702	7	130466854	LOC105375508 (near KLF14)	Intron	C	T	0.48	0.590	1.00E-15	No	rs972283	A	HDL	(48)	99 900
	rs11776767	8	10683929	PINX1	Intron	C	G	0.40	2.010	1.00E-08	Yes		C	TG	(48)	96 598
	rs1495741	8	18272881	NAT2-PSD3		G	A	0.22	2.850	5.00E-14	Yes		G	TG	(48)	96 580
	rs12678919	8	19844222	LPL-SLC18A1		A	G	0.88	13.640	2.00E-115	Yes		A	TG	(48)	96 598
	rs2081687	8	59388565	UBXN2B-CYP7A1		T	C	0.35	1.230	2.00E-12	Yes		T	TC	(48)	100 184
	rs2293889	8	116599199	TRPS1	Intron	T	G	0.41	0.440	6.00E-11	Yes		T	HDL	(48)	99 900
	rs2954029	8	126481747	TRIB1-LOC105375746		A	T	0.47	5.640	3.00E-55	No	rs2980875	A	TG	(48)	96 598
	rs11136341	8	145043543	PLEC1	Intron	G	A	0.40	1.400	4.00E-13	Yes		G	LDL	(48)	88 376
	rs581080	9	15305378	TTC39B	Intron	G	C	0.18	0.650	3.00E-12	Yes		G	HDL	(48)	99 889
	rs1883025	9	107664301	ABCA1	Intron	T	C	0.25	0.940	2.00E-33	Yes		T	HDL	(48)	99 179
	rs9411489	9	133274414	ABO-SURF6		T	C	0.20	2.240	6.00E-13	No	rs532436	A	LDL	(48)	95 454
	rs10761731	10	65027610	JMJD1C	Intron	A	T	0.57	2.380	3.00E-12	Yes		A	TG	(48)	96 598
	rs2255141	10	113933886	GPAM	Intron	A	G	0.30	1.140	2.00E-10	Yes		A	TC	(48)	100 184
	rs2923084	11	10388782	LOC100130460	Intron	G	A	0.17	0.410	5.00E-08	Yes		G	HDL	(48)	99 898
	rs10128711	11	18632984	SPTY2D1	Intron	C	T	0.72	1.040	3.00E-08	Yes		C	TC	(48)	100 184
	rs3136441	11	46724717	F2	Intron	T	C	0.85	0.780	3.00E-18	No	rs148055528	Ins	HDL	(48)	99 900
	rs174546	11	61596633	FADS1	3'UTR	T	C	0.34	3.820	5.00E-24	No	rs99780	T	TG	(48)	96 598
	rs964184	11	116648917	ZPR1	3'UTR	G	C	0.13	16.950	7.00E-240	Yes		G	TG	(48)	96 576
	rs7941030	11	122522375	BLID-UBASH3B		C	T	0.38	0.970	2.00E-10	Yes		C	TC	(48)	100 184
	rs11220462	11	126243952	ST3GAL4	Intron	A	G	0.14	1.950	1.00E-15	Yes		A	LDL	(48)	95 454
**Lipids**	rs7134375	12	20473758	LOC105369688	Non coding	C	A	0.58	0.400	4.00E-08	Yes		C	HDL	(48)	99 900
	rs11613352	12	57738600	R3HDM2	Intron	C	T	0.77	2.700	4.00E-10	No	rs79395356	T	TG	(48)	96 598
	rs7134594	12	110000193	MMAB	Intron	C	T	0.47	0.440	7.00E-15	Yes		C	HDL	(48)	99 900
	rs1169288	12	121416988	HNF1A	Exon non-syn.	C	A	0.33	1.420	1.00E-14	No	rs2244608	C	TC	(48)	100 184
	rs4759375	12	123796238	SBNO1	Intron	C	T	0.94	0.860	7.00E-09	Yes		C	HDL	(48)	99 900

Risk Group	Original SNP	Chr	Position of original SNP (GRCh37)	Locus (Gene)	Functional Consequence	Alleles		EAF	European ancestry values as published		Present in ADVANCE?	Best ADVANCE Proxy	ADVANCE Effect Allele	Risk Factor	Ref	Nb. of Ind
						Effect	Other		β	p-value						
	rs4765127	12	124460167	ZNF664-FAM101A, ZNF664	Intron, Intron	G	T	0.66	0.440	3.00E-10	Yes		G	HDL	(48)	99 787
	rs2929282	15	44259429	FRMD5	Intron	T	A	0.05	5.130	2.00E-11	No	rs34586661	C	TG	(48)	95 070
	rs1532085	15	58683366	LOC101928635 (near LIPC)	Intron	G	A	0.39	1.450	3.00E-96	Yes		G	HDL	(48)	98 409
	rs11649653	16	30918487	CTF1-FBXL19		C	G	0.60	2.130	3.00E-08	Yes		C	TG	(48)	95 034
	rs3764261	16	56989590	HERPUD1-CETP		C	A	0.68	3.390	7,0E-380	No	rs247616	C	HDL	(48)	94 225
	rs16942887	16	67931826	PSKH1	Intron	G	A	0.88	1.270	8.00E-33	No	rs8054091	A	HDL	(48)	98 409
	rs2000999	16	72108093	HPR, TXNL4B	Intron, Intron	A	G	0.20	2.340	3.00E-24	Yes		A	TC	(48)	98 656
	rs2925979	16	81534790	CMIP	Intron	T	C	0.30	0.450	2.00E-11	Yes		T	HDL	(48)	98 409
	rs11869286	17	37813856	STARD3	Intron	G	C	0.34	0.480	1.00E-13	Yes		G	HDL	(48)	98 409
	rs7206971	17	45425115	EFCAB13	Intron	A	G	0.49	0.780	2.00E-08	Yes		A	LDL	(48)	93 999
	rs4148008	17	66893172	ABCA8	Intron	G	C	0.32	0.420	2.00E-10	No	rs8065105	G	HDL	(48)	98 409
	rs4129767	17	76403984	PGS1	Intron	G	A	0.51	0.390	8.00E-09	Yes		G	HDL	(48)	98 409
	rs7241918	18	47160953	LOC105372112	Intron	G	T	0.17	1.310	3.00E-49	Yes		G	HDL	(48)	98 409
	rs12967135	18	57839769	CCBE1-CDH20		A	G	0.23	0.420	7.00E-09	No	rs571312	A	HDL	(48)	98 409
	rs737337	19	11347493	DOCK6	Exon syn.	C	T	0.08	0.640	3.00E-09	Yes		C	HDL	(48)	98 409
	rs10401969	19	19407718	SUGP1	Intron	T	C	0.93	4.740	3.00E-38	Yes		T	TC	(48)	98 640
	rs2277862	20	34152782	FER1L4	Non coding	C	T	0.85	1.190	4.00E-10	Yes		C	TC	(48)	98 656
	rs2902940	20	39091487	LOC105372618	Intron	A	G	0.71	1.380	6.00E-11	Yes		A	TC	(48)	98 656
	rs6029526	20	39672618	TOP1	Intron	A	T	0.47	1.390	4.00E-19	Yes		A	LDL	(48)	93 999
	rs6065906	20	44590298	PLTP-PCIF1		C	T	0.18	0.930	2.00E-22	No	rs6073972	G	HDL	(48)	98 409
	rs181362	22	21982249	UBE2L3	Intron	T	C	0.20	0.460	1.00E-08	No	rs878825	C	HDL	(48)	96 905
	rs5756931	22	38546033	PLA2G6	Intron	T	C	0.60	1.540	4.00E-08	Yes		T	TG	(48)	95 067
**Stroke**	rs225132	1	8095500	ERRFI1-SLC45A1		T	G	NR	0.117	6.40E-08	Yes		T	Stroke	(49)	74 393
	rs17114036	1	56962821	PPAP2B	intron	A	G	NR	0.139	9.78E-09	Yes		A	Stroke	(49)	109 124
	rs602633	1	109821511	CELSR2-PSRC1		G	T	NR	0.105	1.42E-08	Yes		G	Stroke	(49)	109 124
	rs12122341	1	115655690	TSPAN2-NGF		G	C	0.26	0.174	1.00E-09	Yes		G	Stroke	(50)	435 001
	rs1800594	1	169510524	F5	Exon syn.	A	G	0.69	0.166	2.26E-06	Yes		A	Stroke	(51)	19 730
**Stroke**	rs13407662	2	53779789	LOC105369165-ASB3		T	C	0.04	0.668	5.18E-08	No	rs67499383	T	Stroke	(52)	74 393
	rs6725887	2	203745885	WDR12	Intron	C	T	NR	0.131	2.37E-08	Yes		C	Stroke	(49)	109 124
	rs9818870	3	138104635	MRAS	3'UTR	T	C	NR	0.104	1.24E-07	No	rs34905952	A	Stroke	(49)	109 124
	rs16851055	3	140799213	SPSB4	3'UTR	G	A	0.81	0.113	6.34E-07	Yes		G	Stroke	(52)	74 393
	rs2200733	4	111710169	PITX2-MIR297		T	C	0.12	0.315	2.79E-32	Yes		T	Stroke	(50)	435 001
	rs1906599	4	111712686	PITX2-MIR297		T	C	0.19	0.372	3.45E-08	Yes		T	Stroke	(53)	50 978
	rs9369640	6	12927544	PHACTR1	intron	A	C	NR	0.094	2.69E-11	No	rs12526453	C	Stroke	(49)	109 124
	rs12205331	6	34898455	ANKS1A	intron	C	T	NR	0.073	6.39E-06	Yes		C	Stroke	(49)	109 124
	rs556621	6	44594159	LOC105375075-LOC101929770		A	C	0.30	0.191	4.70E-08	Yes		A	Stroke	(54)	56 075

Risk Group	Original SNP	Chr	Position of original SNP (GRCh37)	Locus (Gene)	Functional Consequence	Alleles		EAF	European ancestry values as published		Present in ADVANCE?	Best ADVANCE Proxy	ADVANCE Effect Allele	Risk Factor	Ref	Nb. of Ind
						Effect	Other		β	p-value						
	rs783396	6	106987370	AIM1	Exon non-syn.	C	A	0.90	0.775	9.24E-06	Yes		C	Stroke	(55)	517
	rs12190287	6	134214525	TCF21	3'UTR	C	T	NR	0.105	2.32E-09	Yes		C	Stroke	(49)	109 124
	rs2048327	6	160867159	SLC22A3	Intron	C	T	NR	0.068	1.34E-06	No	rs9355288	A	Stroke	(49)	109 124
	rs10486776	7	15742786	LOC101927558	Intron	A	G	0.10	1.726	6.00E-06	Yes		A	Stroke	(55)	517
	rs2107595	7	19049388	HDAC9-TWIST1		A	G	0.16	0.329	2.03E-16	Yes		A	Stroke	(52)	74 393
	rs11556924	7	129663496	ZC3HC1	Exon non-syn.	C	T	NR	0.094	2.66E-10	Yes		C	Stroke	(49)	74 393
	rs264	8	19813180	LPL	intron	G	A	NR	0.105	2.92E-07	Yes		G	Stroke	(49)	109 124
	rs1333047	9	22124504	CDKN2B-AS1-DMRTA1		T	A	NR	0.182	1.64E-06	Yes		T	Stroke	(49)	74 393
	rs1746048	10	44775824	LINC00841-CXCL12		C	T	0.84	0.131	8.14E-11	Yes		C	Stroke	(56)	24 864
	rs12413409	10	104719096	CNNM2	intron	G	A	NR	0.117	1.24E-06	Yes		G	Stroke	(49)	109 124
	rs11196288	10	115056097	LOC105378489-LOC105378490		G	A	0.05	0.344	1.50E-08	No	rs7906302	G	Stroke	(51)	19 730
	rs9326246	11	116611733	LOC101929011-BUD13		C	G	NR	0.140	3.40E-07	Yes		C	Stroke	(49)	109 124
	rs12425791	12	775199	LOC105369597 (near NINJ2)	Non coding	A	G	0.23	0.239	1.00E-09	No	rs11833579	A	Stroke	(57)	2 196
	rs3184504	12	111865049	SH2B3	Exon non-syn.	T	C	NR	0.068	9.33E-07	No	rs7310615	C	Stroke	(49)	109 124
	rs2238151	12	112211833	ALDH2	Intron	T	C	0.66	0.122	1.03E-06	Yes		T	Stroke	(52)	74 393
	rs17696736	12	112553032	NAA25	Intron	G	A	NR	0.095	5.96E-08	No	rs10850001	A	Stroke	(49)	74 393
	rs4304924	13	79238925	RNF219-LINC00331		A	G	NR	0.163	4.50E-06	Yes		A	Stroke	(49)	74 393
	rs12435908	14	66121468	FUT8	Intron	A	C	0.93	0.255	7.19E-06	Yes		A	Stroke	(51)	19 730
	rs1005224	14	76173860	TTLL5	intron	A	T	NR	0.186	1.47E-06	Yes		A	Stroke	(49)	74 393
	rs1465330	14	77600584	ZDHHC22	intron	A	T	NR	0.174	3.78E-06	No	rs963474	G	Stroke	(49)	74 393
	rs7173743	15	79141784	ADAMTS7-MORF4L1		T	C	NR	0.073	6.70E-08	Yes		T	Stroke	(49)	109 124
	rs7193343	16	73029160	ZFHX3	Intron	T	C	0.17	0.157	2.29E-10	Yes		T	Stroke	(50)	435 001
**Stroke**	rs879324	16	73068678	ZFHX3	Intron	A	G	0.19	0.223	2.28E-08	Yes		A	Stroke	(52)	74 393
	rs2281727	17	2117945	SMG6	Intron	G	A	NR	0.068	1.07E-07	Yes		G	Stroke	(49)	109 124
	rs4792143	17	11385890	SHISA6	intron	C	T	NR	0.094	5.07E-06	Yes		C	Stroke	(49)	74 393
	rs12936587	17	17543722	PEMT-RAI1		G	A	NR	0.073	1.98E-07	Yes		G	Stroke	(49)	109 124
	rs6565653	17	78201783	SLC26A11	Intron	T	G	0.46	0.182	5.51E-07	Yes		T	Stroke	(51)	19 730
	rs1122608	19	11179709	SMARCA4	Intron	G	T	0.75	0.128	3.32E-11	No	rs73013202	C	Stroke	(49)	109 124
	rs9982601	21	35599128	LINC00310-KCNE2		T	C	NR	0.166	2.52E-10	Yes		T	Stroke	(49)	109 124
**Cardiovascular**	rs11206510	1	55496039	BSND-PCSK9		T	C	0.81	0.140	9.60E-09	Yes		T	MI	(56)	25 538
	rs9970807	1	56965664	PLPP3	Intron	C	T	0.92	0.113	1.79E-09	No	rs17114036	A	MI	(58)	171 873
	rs56170783	1	57016731	PLPP3	Intron	A	C	0.92	0.104	2.10E-12	No	rs17114036	A	CAD	(59)	336 000
	rs646776	1	109818530	CELSR2	Downstr. 500B	T	C	0.81	0.174	7.90E-12	Yes		T	MI	(56)	25 538
	rs646776	1	109818530	CELSR2	Downstr. 500B	T	C	0.81	0.157	9.36E-11	Yes		T	CAD	(56)	24 864
	rs11810571	1	151770138	TDRKH	Intron	G	C	0.79	0.055	4.24E-08	No	rs4845579	C	CAD	(59)	345 035
	rs6689306	1	154395946	IL6R	Intron	A	G	0.44	0.049	1.50E-09	No	rs12118721	T	CAD	(59)	336 000

Risk Group	Original SNP	Chr	Position of original SNP (GRCh37)	Locus (Gene)	Functional Consequence	Alleles		EAF	European ancestry values as published		Present in ADVANCE?	Best ADVANCE Proxy	ADVANCE Effect Allele	Risk Factor	Ref	Nb. of Ind
						Effect	Other		β	p-value						
	rs12118721	1	154397416	IL6R	Intron	T	C	0.48	0.058	1.17E-07	Yes		T	MI	(58)	171 874
	rs13376333	1	154814353	KCNN3	Intron	T	C	0.30	0.445	6.30E-12	Yes		T	AF	(60)	14 179
	rs10911021	1	182073820	ZNF648, LOC105371642	Intron, Intron	C	T	0.68	0.307	2.04E-08	No	rs61063076	C	CHD	(61)	6 562
	rs17465637	1	222823529	MIA3	Intron	C	A	0.72	0.131	1.40E-09	Yes		C	MI	(56)	25 538
	rs17465637	1	222823529	MIA3	Intron	C	A	0.72	0.122	1.33E-08	Yes		C	CAD	(56)	24 864
	rs67180937	1	222823743	MIA3	Intron	G	T	0.68	0.068	8.50E-14	Yes		G	CAD	(59)	336 000
	rs585967	2	21270554	APOB-TDRD15		C	A	0.84	0.068	2.80E-08	Yes		C	CAD	(59)	336 000
	rs4299376	2	44072576	ABCG8 (near ABCG5)	Intron	G	T	0.32	0.058	5.70E-10	Yes		G	CAD	(59)	336 000
	rs10176176	2	85762048	SH2D6-MAT2A		T	A	0.47	0.068	2.88E-10	Yes		T	MI	(58)	171 874
	rs7568458	2	85788175	GGCX	Intron	A	T	0.45	0.058	2.40E-13	Yes		A	CAD	(59)	336 000
	rs17678683	2	145286559	LINC01412 (near ZEB2)	Intron	G	T	0.09	0.077	1.20E-07	Yes		G	CAD	(59)	336 000
	rs6725887	2	203745885	WDR12	Intron	C	T	0.14	0.157	1.30E-08	Yes		C	MI	(56)	25 538
	rs114123510	2	203831212	CARF	Intron	A	T	0.12	0.122	2.90E-19	No	rs6725887	C	CAD	(59)	336 000
	rs1250229	2	216300185	FN1-LOC102724849		T	C	0.26	0.069	2.77E-13	No	rs1250258	T	CAD	(59)	270 189
	rs13003675	2	233584109	GIGYF2	Intron	T	C	0.36	0.039	1.70E-06	Yes		T	CAD	(59)	336 000
	rs7623687	3	49448566	RHOA, TCTA	Intron, Upstr. 2KB	A	C	0.86	0.073	3.44E-10	Yes		A	CAD	(59)	333 836
	rs142695226	3	124475201	UMPS-ITGB5		G	T	0.14	0.069	1.53E-09	Yes		G	CAD	(59)	345 188
	rs12493885	3	153863505	ARHGEF26	Exon non-syn.	C	G	0.87	0.070	3.16E-08	No	rs1713822	G	CAD	(59)	251 805
	rs72627509	4	57839051	NOA1	Intron	G	C	0.20	0.058	8.10E-08	Yes		G	CAD	(59)	336 000
Cardiovascular	rs10857147	4	81164723	PRDM8-FGF5		T	A	0.27	0.053	5.66E-09	No	rs1458038	T	CAD	(59)	345 133
	rs17042171	4	111710169	PITX2-LOC729065		A	C	0.15	0.501	6.00E-27	No	rs2200733	T	AF	(62)	40 518
	rs7678555	4	120900282	LOC100996694-MAD2L1		C	A	0.28	0.051	1.32E-08	No	rs13134800	T	CAD	(59)	345 126
	rs4593108	4	148276400	MIR548G-EDNRA		C	G	0.80	0.068	3.75E-07	No	rs2059904	A	MI	(58)	166 765
	rs6841581	4	148427503	EDNRA	Upstream 2KB	A	G	0.15	0.068	4.60E-10	No	rs78049276	C	CAD	(59)	336 000
	rs2306556	4	156638573	GUCY1A3	Intron	A	G	0.82	0.068	1.20E-09	No	rs72689147	G	CAD	(59)	336 000
	rs72689147	4	156639888	GUCY1A3	Intron	G	T	0.82	0.077	1.63E-08	Yes		G	MI	(58)	171 874
	rs7692395	4	183624937	TENM3	Intron	G	T	NR	0.371	2.50E-07	No	rs9784414	G	MI	(63)	64 297
	rs9349379	6	12903957	PHACTR1	Intron	G	A	0.41	0.104	1.00E-35	Yes		G	CAD	(59)	336 000
	rs12526453	6	12927544	PHACTR1	Intron	C	G	0.65	0.113	1.30E-09	Yes		C	MI	(56)	25 538
	rs6909752	6	22612629	LOC105374971-LOC105374972		A	G	0.33	0.050	2.19E-09	Yes		A	CAD	(59)	343 171
	rs3130683	6	31888367	C2	Intron	T	C	0.86	0.077	2.80E-08	Yes		T	CAD	(59)	336 000
	rs4472337	6	34769765	UHRF1BP1	Intron	T	C	0.16	0.058	2.40E-06	Yes		T	CAD	(59)	336 000
	rs1544935	6	39140125	SAYSD1-KCNK5		T	G	0.81	0.077	2.89E-08	No	rs755852	A	MI	(58)	169 427
	rs56015508	6	39152041	SAYSD1-KCNK5		C	A	0.79	0.058	1.10E-07	Yes		C	CAD	(59)	336 000
	rs2916260	6	40382711	LRFN2	Intron	T	C	NR	0.140	1.80E-06	Yes		T	CHD	(63)	64 297
	rs12202017	6	134196381	LINC031312, TARID	Non coding, Intron	A	G	0.70	0.068	6.00E-14	No	rs7769954	A	CAD	(59)	336 000
	rs6922269	6	151252985	MTHFD1L	Intron	A	G	0.30	0.207	6.33E-06	Yes		A	CAD	(64)	7 383

Risk Group	Original SNP	Chr	Position of original SNP (GRCh37)	Locus (Gene)	Functional Consequence	Alleles		EAF	European ancestry values as published		Present in ADVANCE?	Best ADVANCE Proxy	ADVANCE Effect Allele	Risk Factor	Ref	Nb. of Ind
						Effect	Other		β	p-value						
	rs10455872	6	161103805	LPA	Intron	G	A	0.07	0.531	3.40E-15	No	rs374071816	A	CAD	(65)	15 937
	rs10455872	6	161103805	LPA	Intron	G	A	0.05	0.285	8.88E-27	No	rs374071816	A	MI	(58)	161 728
	rs2315065	6	161123451	PLG-LPA		A	C	0.06	0.262	2.34E-24	No	rs4252185	C	MI	(58)	161 728
	rs6941513	6	164030800	QKI-LOC102724152		G	A	NR	0.182	6.20E-09	Yes		G	MI	(63)	64 297
	rs4721377	7	14855062	DGKB	Intron	T	C	NR	0.231	1.90E-06	Yes		T	MI	(63)	64 297
	rs12669789	7	18734065	HDAC9	Intron	C	T	NR	0.262	1.73E-06	Yes		C	ICA	(66)	12 002
	rs7798197	7	19037661	HDAC9	3'UTR	A	G	NR	0.166	4.67E-06	Yes		A	ICA	(66)	12 002
	rs2107595	7	19049388	HDAC9-TWIST1		A	G	0.18	0.077	3.40E-13	Yes		A	CAD	(59)	336 000
	rs10230207	7	19611307	LOC105375179-LOC105375180		T	G	NR	0.191	9.91E-10	Yes		T	ICA	(66)	12 002
	rs112370447	7	107100794	COG5	Intron	T	C	0.28	0.049	9.60E-07	No	rs987391	G	CAD	(59)	336 000
	rs11556924	7	129663496	ZC3HC1	Exon non-syn.	C	T	0.66	0.068	6.30E-13	Yes		C	CAD	(59)	336 000
	rs264	8	19813180	LPL	intron	G	A	NR	0.105	2.92E-07	Yes		G	CAD	(49)	109 124
	rs2083636	8	19865263	LPL-LOC105379311		T	G	0.74	0.049	6.40E-08	Yes		T	CAD	(59)	336 000
	rs2001846	8	126478450	TRIB1-LOC105375746		T	C	0.49	0.049	8.97E-07	Yes		T	MI	(58)	170 153
Cardiovascular	rs2954029	8	126481747	TRIB1-LOC105375746		A	T	0.54	0.058	5.20E-13	No	rs2980875	A	CAD	(59)	336 000
	rs6475606	9	22081850	CDKN2B-AS1	Intron	T	C	0.48	0.300	3.59E-08	No	rs1537370	T	ICA	(67)	3 166
	rs1537370	9	22084310	CDKN2B-AS1	Intron	T	C	0.46	0.532	2.35E-11	Yes		C	CAC	(68)	2 620
	rs4977574	9	22124504	CDKN2B-AS1	Intron	T	A	0.56	0.247	1.08E-41	No	rs1333047	T	CAD	(56)	24 864
	rs4977574	9	22124504	CDKN2B-AS1	Intron	T	A	0.56	0.255	2.70E-44	No	rs1333047	T	MI	(56)	25 538
	rs1333049	9	22125503	CDKN2B-AS1-DMRTA1		C	G	0.46	0.199	3.33E-24	Yes		G	CAC	(69)	15 993
	rs514659	9	136144308	ABO	Intron	C	A	0.40	0.191	7.62E-09	No	rs576123	C	CAD	(70)	9 427
	rs514659	9	136144308	ABO	Intron	C	A	0.37	0.191	7.62E-09	No	rs576123	C	MI	(70)	9 427
	rs532436	9	136149830	ABO	Intron	A	G	0.19	0.113	2.31E-17	Yes		A	MI	(58)	169 997
	rs1887318	10	30321598	KIAA1462	Intron	T	C	0.43	0.058	4.10E-12	No	rs2505083	C	CAD	(59)	336 000
	rs2505083	10	30335122	KIAA1462	Intron	C	T	0.39	0.058	6.85E-09	Yes		C	MI	(58)	169 421
	rs1870634	10	44480811	LINC00841-CXCL12		G	T	0.65	0.058	5.50E-13	Yes		G	CAD	(59)	336 000
	rs1746048	10	44775824	LINC00841-CXCL12		C	T	0.84	0.131	8.14E-11	Yes		C	CAD	(56)	24 864
	rs1746048	10	44775824	LINC00841-CXCL12		C	T	0.84	0.157	7.40E-09	Yes		C	MI	(56)	25 538
	rs1004467	10	104594507	CYP17A1	Intron	A	G	0.87	0.077	7.71E-08	No	rs12413409	G	MI	(58)	171 875
	rs12413409	10	104719096	CNNM2	intron	G	A	NR	0.077	1.24E-06	Yes		G	CAD	(49)	109 124
	rs10840293	11	9751196	SWAP70	Intron	A	G	0.55	0.049	6.90E-09	Yes		A	CAD	(59)	336 000
	rs3993105	11	13300252	ARNTL	Intron	T	C	0.68	0.047	4.77E-08	No	rs7928655	G	CAD	(59)	340 699
	rs2019090	11	103668962	LOC105369463-LOC102723862		A	T	0.36	0.068	3.60E-09	Yes		A	MI	(58)	166 655
	rs2839812	11	103673294	LOC105369463-LOC102723862		T	A	0.31	0.058	2.00E-11	No	rs2019090	A	CAD	(59)	336 000
	rs12280753	11	116613660	LOC101929011-BUD13		C	T	0.95	0.371		Yes		C	CAD	(59)	336 000

Risk Group	Original SNP	Chr	Position of original SNP (GRCh37)	Locus (Gene)	Functional Consequence	Alleles		EAF	European ancestry values as published		Present in ADVANCE?	Best ADVANCE Proxy	ADVANCE Effect Allele	Risk Factor	Ref	Nb. of Ind
						Effect	Other		β	p-value						
	rs964184	11	116648917	ZPR1 (near APOA5, BUD13)	3'UTR	G	C	0.16	0.049	4.70E-06	Yes		G	CAD	(59)	336 000
	rs7121446	11	121954657	MIR100HG	Intron	A	G	0.24	1.897		Yes		A	CAD	(59)	336 000
	rs2229357	12	57738600	INHBC	Exon non-syn.	G	A	0.76	0.049	3.40E-06	No	rs79395356	T	CAD	(59)	336 000
	rs2681472	12	90008959	ATP2B1	Intron	G	A	0.19	0.068	7.60E-11	Yes		G	CAD	(59)	336 000
	rs3184504	12	111865049	SH2B3	Exon non-syn.	T	C	NR	0.068	9.33E-07	No	rs7310615	C	CAD	(49)	109 124
	rs3184504	12	111865049	SH2B3	Exon non-syn.	T	C	NR	0.068	9.33E-07	No	rs7310615	C	MI	(49)	109 124
	rs653178	12	112007756	ATXN2	Intron	C	T	0.44	0.077	2.80E-11	No	rs7310615	C	MI	(58)	167 530
	rs2244608	12	121416988	HFN1A	Intron	G	A	0.35	0.052	7.74E-10	Yes		G	CAD	(59)	345 106
	rs11057830	12	125307053	SCARB1	Intron	A	G	0.15	0.068	4.20E-09	Yes		A	CAD	(59)	336 000
	rs16945184	13	91755199	LINC00380-LOC105370312		C	T	NR	0.186	5.80E-06	Yes		C	CHD	(63)	64 297
Cardiovascular	rs11617955	13	110818102	COL4A1	Intron	T	A	0.89	0.086	4.10E-10	Yes		T	CAD	(59)	336 000
	rs55940034	13	111043309	COL4A2	Intron	G	A	0.27	0.068	7.62E-08	Yes		G	MI	(58)	166 228
	rs1924981	13	290226415	FLT1	Intron	T	C	0.33	0.049	1.90E-07	Yes		T	CAD	(59)	336 000
	rs10139550	14	100145710	HHIPL1	3'UTR	G	C	0.42	0.049	1.80E-09	Yes		G	CAD	(59)	336 000
	rs72743461	15	67441750	SMAD3	Intron	C	A	0.78	0.068	4.80E-12	Yes		C	CAD	(59)	336 000
	rs1994016	15	79080234	ADAMTS7	Intron	C	T	0.61	0.199	2.41E-12	Yes		C	CAD	(70)	19 776
	rs3825807	15	79089111	ADAMTS7	Exon non-syn.	T	C	0.57	1.013	6.50E-06	No	rs1994016	C	CAC	(68)	2 620
	rs7164479	15	79123054	ADAMTS7-MORF4L1		C	T	0.58	0.068	6.40E-18	No	rs7165042	C	CAD	(59)	336 000
	rs7165042	15	79123338	ADAMTS7-MORF4L1		C	T	0.56	0.058	3.02E-09	Yes		C	MI	(58)	169 997
	rs2083460	15	89574484	MFGE8-ABHD2		T	C	0.89	0.068	1.40E-07	Yes		T	CAD	(59)	336 000
	rs247616	16	56989590	HERPUD1-CETP		C	T	0.68	0.039	1.00E-06	Yes		C	CAD	(59)	336 000
	rs2106261	16	73068678	ZFHX3	Intron	T	C	0.22	0.223	2.30E-07	No	rs879324	A	AF	(62)	40 518
	rs7500448	16	83045790	CDH13	Intron	A	G	0.77	0.061	4.76E-10	Yes		A	CAD	(59)	337 399
	rs4843416	16	86676764	FOXL1-LOC102724344		G	A	NR	0.357	6.50E-06	No	rs16941789	C	MI	(63)	64 297
	rs113348108	17	2088849	SMG6	Intron	Del	CAGA	0.31	0.049	5.80E-08	No	rs4790883	C	CAD	(59)	336 000
	rs9914266	17	2117945	SMG6	Intron	G	A	0.35	0.049	2.55E-06	No	rs2281727	G	MI	(58)	171 874
	rs9897596	17	17593453	RAI1	Intron	T	C	0.52	0.039	3.10E-06	Yes		T	CAD	(59)	336 000
	rs35895680	17	47060322	GIP-IGF2BP1		C	A	0.72	0.058	7.77E-06	Yes		C	MI	(58)	170 347
	rs4643373	17	47123423	IGF2BP1	Intron	T	C	0.72	0.049	1.20E-06	Yes		T	CAD	(59)	336 000
	rs7212798	17	59013488	BCAS3	Intron	C	T	0.15	0.068	8.06E-06	Yes		C	MI	(58)	171 873
	rs8068952	17	59286644	BCAS3	Intron	G	C	0.23	0.068	1.40E-09	Yes		G	CAD	(59)	336 000
	rs1122608	19	11179709	SMARCA4	Intron	G	T	0.75	0.140	1.90E-09	No	rs73013202	C	MI	(56)	25 538
	rs8108632	19	41854534	TGFB1	Intron	T	A	0.48	0.047	4.04E-08	Yes		T	CAD	(59)	345 058
	rs56131196	19	45422946	APOC1	Downstr.500B	A	G	0.16	0.086	3.87E-08	No	rs4420638	G	MI	(58)	163 760
	rs28451064	21	35593827	LINC00310-LOC105372791		A	G	0.12	0.131	2.60E-23	No	rs9982601	T	CAD	(59)	336 000
	rs9982601	21	35599128	MRPS6-KCNE2		T	C	0.13	0.182	6.40E-11	Yes		T	MI	(56)	25 538

Risk Group	Original SNP	Chr	Position of original SNP (GRCh37)	Locus (Gene)	Functional Consequence	Alleles		EAF	European ancestry values as published		Present in ADVANCE?	Best ADVANCE Proxy	ADVANCE Effect Allele	Risk Factor	Ref	Nb. of Ind
						Effect	Other		β	p-value						
Low Birth Weight	rs2473248	1	22546110	WNT4-ZBTB40		C	T	0.87	0.033	1.10E-08	No	rs2744729	T	Birth Weight	(71)	139 428
	rs4330912	1	155964698	LOC107985208, LOC107985210	Intron	G	C	0.64	0.021	1.80E-07	No	rs12043212	A	Birth Weight	(71)	143 677
	rs72480273	1	161646824	FCGR2B	Intron	C	A	0.17	0.031	8.00E-10	No	rs12118043	C	Birth Weight	(71)	138 380
	rs61830764	1	212289976	DTL-PPP2R5A		A	G	0.38	0.022	5.60E-08	Yes		G	Birth Weight	(71)	138 158
Low Birth Weight	rs7575873	2	23902360	ATAD2B	Intron	A	G	0.88	0.038	1.30E-11	No	rs7578606	C	Birth Weight	(71)	139 425
	rs1374204	2	46484205	LOC101926974	Intron	T	C	0.70	0.047	6.20E-29	Yes		C	Birth Weight	(71)	134 453
	rs2168443	3	46947087	PTH1R-CCDC12		T	A	0.38	0.023	3.50E-09	Yes		A	Birth Weight	(71)	143 670
	rs11719201	3	123069058	ADCY5	Intron	T	C	0.23	0.046	2.40E-26	No	rs11720108	C	Birth Weight	(71)	143 670
	rs10935733	3	148622968	CPA3-GYG1		T	C	0.41	0.022	9.20E-09	Yes		C	Birth Weight	(71)	139 426
	rs900399	3	156798732	LEKR1-LINC00880		A	G	0.61	0.052	2.20E-41	Yes		G	Birth Weight	(71)	139 426
	rs2724475	4	17946432	LCORL	Intron	T	C	0.27	0.034	5.30E-16	Yes		C	Birth Weight	(71)	139 426
	rs2131354	4	145599908	HHIP	Intron	A	G	0.53	0.026	4.10E-12	Yes		G	Birth Weight	(71)	139 431
	rs4432842	5	57172078	LOC101928505-LOC101928539		T	C	0.70	0.021	3.40E-07	Yes		C	Birth Weight	(71)	139 429
	rs2946179	5	157886627	LOC101927697-EBF1		C	T	0.73	0.024	1.30E-08	Yes		T	Birth Weight	(71)	143 669
	rs35261542	6	20675792	CDKAL1	Intron	C	A	0.73	0.044	4.40E-27	Yes		A	Birth Weight	(71)	143 667
	rs9379832	6	26186200	HIST1H2BE-HIST1H4D		A	G	0.70	0.023	6.60E-08	Yes		G	Birth Weight	(71)	138 161
	rs9368777	6	33788637	LOC105375025	Intron	C	G	0.58	0.022	2.20E-08	Yes		G	Birth Weight	(71)	143 677
	rs1187118	6	34169020	GRM4-HMGA1		A	T	0.17	0.030	3.60E-09	Yes		T	Birth Weight	(71)	137 045
	rs1415701	6	130345835	L3MBTL3	Intron	G	A	0.74	0.025	2.60E-09	Yes		A	Birth Weight	(71)	143 666
	rs10872678	6	152039964	ESR1	Intron	T	C	0.72	0.038	6.90E-20	Yes		C	Birth Weight	(71)	139 427
	rs798489	7	2801803	GNA12, AMZ1	Intron, Intron	C	T	0.73	0.023	2.00E-08	Yes		T	Birth Weight	(71)	143 670
	rs11765649	7	23479013	IGF2BP3	Intron	T	C	0.75	0.027	5.80E-10	Yes		C	Birth Weight	(71)	139 428
	rs6959887	7	35295365	TBX20	Upstream 2KB	A	G	0.61	0.023	1.50E-09	Yes		G	Birth Weight	(71)	143 659

Risk Group	Original SNP	Chr	Position of original SNP (GRCh37)	Locus (Gene)	Functional Consequence	Alleles		EAF	European ancestry values as published		Present in ADVANCE?	Best ADVANCE Proxy	ADVANCE Effect Allele	Risk Factor	Ref	Nb. of Ind
						Effect	Other		β	p-value						
	rs62466330	7	73056805	MLXIPL-VPS37D		C	T	0.07	0.049	1.20E-10	Yes		T	Birth Weight	(71)	142 200
**Low Birth Weight**	rs13266210	8	41533514	ANK1	Intron	A	G	0.79	0.031	1.30E-11	Yes		G	Birth Weight	(71)	139 429
	rs6989280	8	126504726	TRIB1-LOC105375746		G	A	0.73	0.022	2.20E-07	No	rs6987702	C	Birth Weight	(71)	143 671
	rs12543725	8	142247979	SLC45A4	5'UTR	G	A	0.59	0.023	1.20E-09	Yes		A	Birth Weight	(71)	139 431
	rs12551019	9	96949079	MIRLET7DHG	Intron	C	T	0.68	0.022	3.10E-08	Yes		T	Birth Weight	(71)	143 677
	rs3780573	9	98239503	PTCH1	Intron	A	G	0.10	0.055	7.00E-18	Yes		G	Birth Weight	(71)	134 746
	rs1411424	9	113892963	LOC105376219	Intron	A	G	0.52	0.021	2.20E-08	Yes		G	Birth Weight	(71)	139 424
	rs4836833	9	123633948	PHF19	Intron	C	G	0.67	0.023	8.60E-09	No	rs3933326	A	Birth Weight	(71)	139 424
	rs10818797	9	126020405	STRBP	Intron	C	T	0.14	0.035	1.20E-10	Yes		T	Birth Weight	(71)	139 427
	rs2497304	10	94492716	HHEX-EXOC6		C	T	0.52	0.028	2.60E-14	Yes		T	Birth Weight	(71)	143 670
	rs79237883	10	104719096	NT5C2	5'UTR	C	T	0.08	0.037	3.60E-08	No	rs12413409	G	Birth Weight	(71)	143 667
	rs740746	10	115798895	LOC105378493-ADRB1		A	G	0.73	0.036	3.80E-18	No	rs2773469	A	Birth Weight	(71)	143 671
	rs2421016	10	124167512	PLEKHA1	Intron	T	C	0.49	0.021	1.80E-08	Yes		C	Birth Weight	(71)	143 659
	rs10830963	11	92708710	MTNR1B	Intron	G	C	0.28	0.023	2.90E-08	Yes		C	Birth Weight	(71)	143 663
	rs11055034	12	12889125	APOLD1	Intron	C	A	0.73	0.022	1.80E-07	No	rs79375472	C	Birth Weight	(71)	143 653
	rs2306547	12	26877885	ITPR2	Intron	C	T	0.54	0.021	1.80E-08	Yes		T	Birth Weight	(71)	139 431
	rs1351394	12	65958046	HMGA2	Intron	T	C	0.49	0.044	1.90E-32	No	rs7312464	G	Birth Weight	(71)	143 671
	rs7964361	12	102994878	LOC105369944	Intron	A	G	0.09	0.039	4.70E-09	Yes		G	Birth Weight	(71)	139 428
	rs7998537	13	40666754	LOC107984569-LINC00332		G	A	0.68	0.022	3.90E-08	No	rs7328783	G	Birth Weight	(71)	139 425
	rs34217484	13	48854550	ITM2B-LINC00441		A	T	0.26	0.024	4.80E-08	Yes		T	Birth Weight	(71)	137 561
	rs1819436	13	78580283	LINC01069-LINC00446		C	T	0.87	0.033	6.30E-09	Yes		T	Birth Weight	(71)	138 979
**Low Birth Weight**	rs7402982	15	99193269	IGF1R	Intron	A	G	0.43	0.023	2.30E-09	Yes		G	Birth Weight	(71)	139 423
	rs1011939	16	19992996	LOC105371117	Intron	G	A	0.30	0.022	1.30E-07	Yes		A	Birth Weight	(71)	143 604

Risk Group	Original SNP	Chr	Position of original SNP (GRCh37)	Locus (Gene)	Functional Consequence	Alleles		EAF	European ancestry values as published		Present in ADVANCE?	Best ADVANCE Proxy	ADVANCE Effect Allele	Risk Factor	Ref	Nb. of Ind
						Effect	Other		β	p-value						
	rs113086489	17	7171356	CLDN7-SLC2A4		T	C	0.56	0.031	9.10E-16	Yes		C	Birth Weight	(71)	139 426
	rs72833480	17	45964861	SP6-LOC102724532		A	G	0.29	0.023	4.60E-08	Yes		G	Birth Weight	(71)	139 421
	rs10402712	19	33926013	PEPD	Intron	A	G	0.26	0.022	4.40E-07	Yes		G	Birth Weight	(71)	139 428
	rs6040076	20	10658882	JAG1-LOC101929395		C	G	0.49	0.023	2.00E-09	Yes		G	Birth Weight	(71)	139 424
	rs28530618	20	31248692	C20orf203-COMMD7		A	G	0.49	0.026	7.70E-12	No	rs11475789	A	Birth Weight	(71)	138 162
	rs6016377	20	39172728	LOC107985446-LOC102724968		T	C	0.43	0.024	9.50E-10	Yes		C	Birth Weight	(71)	139 425
	rs2229742	21	16353809	NRIP1	Exon non-syn.	G	C	0.87	0.036	2.20E-09	No	rs17274750	A	Birth Weight	(71)	143 672
	rs134594	22	29468456	C22orf31, KREMEN1	Intron, Upstr. 2KB	C	T	0.35	0.023	1.00E-08	Yes		T	Birth Weight	(71)	137 340

ESM Table 4: Effect of adding treatment assignment as a covariate on the performance of the multiPRS model.

Outcomes	AUCs of multiPRS model (95%CI)	
	**No Treatment Assignment**	**With Treatment Assignment**
**Macroalbuminuria**	**0.65** (0.60-0.69)	**0.66** (0.61-0.70)
**Low eGFR**	**0.64** (0.62-0.66)	**0.65** (0.63-0.67)
**New or worsening nephropathy**	**0.64** (0.60-0.68)	**0.64** (0.60-0.68)
**Major microvascular**	**0.67** (0.64-0.70)	**0.67** (0.64-0.70)
**Major macrovascular**	**0.68** (0.66-0.70)	**0.68** (0.66-0.71)
**Combined major micro or macrovascular**	**0.67** (0.65-0.70)	**0.67** (0.65-0.70)
**Stroke**	**0.66** (0.62-0.71)	**0.67** (0.62-0.71)
**Myocardial infarction**	**0.67** (0.63-0.70)	**0.67** (0.63-0.71)
**Heart failure**	**0.68** (0.65-0.72)	**0.69** (0.65-0.72)
**Cardiovascular death**	**0.72** (0.69-0.75)	**0.73** (0.69-0.76)
**All cause death**	**0.69** (0.67-0.72)	**0.69** (0.67-0.72)

ESM Table 5: Effect of individual wPRS, combined 10wPRS and 10wPRS + PC1 on prevalent and incident cases determined by single wPRS.

	AUC<br>95% CI											
	**PRS_1**	**PRS_2**	**PRS_3**	**PRS_4**	**PRS_5**	**PRS_6**	**PRS_7**	**PRS_8**	**PRS_9**	**PRS_10**	**10 PRS**	**10 PRS+ PC1**
**Prevalent**												
Low eGFR	0.51<br>0.49, 0.53	0.50<br>0.48, 0.52	0.51<br>0.49, 0.53	0.51<br>0.49, 0.53	0.50<br>0.48, 0.53	0.51<br>0.49, 0.53	0.54<br>0.52, 0.56	0.51<br>0.49, 0.53	0.51<br>0.49, 0.53	0.51<br>0.49, 0.53	0.55<br>0.53, 0.57	**0.56**<br>0.53, 0.58
Stroke	0.53<br>0.50, 0.56	0.53<br>0.50, 0.56	0.53<br>0.50, 0.56	0.50<br>0.47, 0.53	0.53<br>0.50, 0.56	0.51<br>0.48, 0.54	0.54<br>0.50, 0.57	0.52<br>0.49, 0.56	0.50<br>0.47, 0.53	0.52<br>0.49, 0.55	0.58<br>0.54, 0.61	**0.59**<br>0.56, 0.62
MI	0.51<br>0.49, 0.52	0.50<br>0.48, 0.52	0.52<br>0.50, 0.54	0.53<br>0.51, 0.55	0.51<br>0.49, 0.53	0.51<br>0.49, 0.53	0.49<br>0.48, 0.51	0.51<br>0.49, 0.53	0.51<br>0.49, 0.53	0.53<br>0.51, 0.55	**0.55**<br>0.53, 0.57	**0.55**<br>0.53, 0.57
HF	0.53<br>0.48, 0.57	0.54<br>0.50, 0.59	0.50<br>0.46, 0.54	0.52<br>0.48, 0.57	0.50<br>0.46, 0.55	0.48<br>0.44, 0.53	0.51<br>0.46, 0.55	0.55<br>0.51, 0.59	0.55<br>0.51, 0.59	0.55<br>0.50, 0.59	0.61<br>0.57, 0.65	**0.70**<br>0.66, 0.74
Macroalb	0.56<br>0.52, 0.60	0.52<br>0.47, 0.56	0.55<br>0.50, 0.59	0.52<br>0.48, 0.57	0.51<br>0.47, 0.56	0.52<br>0.48, 0.56	0.50<br>0.46, 0.55	0.53<br>0.48, 0.57	0.56<br>0.52, 0.60	0.52<br>0.48, 0.56	**0.60**<br>0.56, 0.64	**0.60**<br>0.56, 0.64
**Incident**												
Low eGFR	0.51<br>0.49, 0.53	0.50<br>0.48, 0.53	0.51<br>0.49, 0.53	0.50<br>0.48, 0.52	0.51<br>0.49, 0.54	0.50<br>0.48, 0.52	0.50<br>0.48, 0.52	0.50<br>0.48, 0.52	0.52<br>0.50, 0.54	0.52<br>0.50, 0.54	**0.54**<br>0.52, 0.56	**0.54**<br>0.52, 0.56
Stroke	0.52<br>0.47, 0.57	0.50<br>0.45, 0.55	0.50<br>0.45, 0.55	0.50<br>0.46, 0.55	0.56<br>0.51, 0.60	0.50<br>0.45, 0.55	0.52<br>0.47, 0.57	0.55<br>0.50, 0.60	0.53<br>0.48, 0.57	0.50<br>0.46, 0.55	0.57<br>0.52, 0.62	**0.61**<br>0.57, 0.66
MI	0.51<br>0.47, 0.56	0.54<br>0.50, 0.59	0.53<br>0.49, 0.57	0.52<br>0.48, 0.56	0.54<br>0.50, 0.58	0.51<br>0.47, 0.55	0.51<br>0.47, 0.55	0.52<br>0.48, 0.56	0.53<br>0.49, 0.58	0.51<br>0.47, 0.56	0.58<br>0.54, 0.63	**0.60**<br>0.56, 0.64
HF	0.52<br>0.49, 0.56	0.52<br>0.48, 0.56	0.51<br>0.47, 0.55	0.53<br>0.49, 0.57	0.55<br>0.51, 0.59	0.52<br>0.48, 0.55	0.51<br>0.47, 0.55	0.51<br>0.48, 0.55	0.50<br>0.47, 0.54	0.50<br>0.47, 0.54	**0.57**<br>0.53, 0.61	**0.57**<br>0.53, 0.61
Macroalb	0.53<br>0.48, 0.57	0.56<br>0.51, 0.61	0.53<br>0.48, 0.57	0.54<br>0.49, 0.60	0.51<br>0.46, 0.55	0.54<br>0.50, 0.59	0.50<br>0.45, 0.55	0.49<br>0.44, 0.53	0.51<br>0.46, 0.56	0.53<br>0.48, 0.57	**0.60**<br>0.56, 0.65	**0.60**<br>0.56, 0.65

PRS_1 : PRS_Albuminuria
PRS_2 : PRS_Biomarkers
PRS_3 : PRS_Blood pressure
PRS_4 : PRS_Cardiovascular
PRS_5 : PRS_Stroke
PRS_6 : PRS_Diabetes
PRS_7 : PRS_Glomerular filtration rate
PRS_8 : PRS_Lipids
PRS_9 : PRS_Obesity
PRS_10 : PRS_Birth weight
PC1: principal component 1; **MI**: Myocardial infarction; **HF**: Heart failure; **Macroalb**: Macroalbuminuria.
The highest AUCs are shown in bold.

ESM Table 6: AUC with confidence intervals for multiPRS model compared to ADVANCE and Framingham clinical scores.

	MultiPRS	Clinical scores			
		ADVANCE		Framingham	
Outcomes	AUC (95%CI)	AUC (95%CI)	p-values vs multiPRS	AUC (95%CI)	p-values vs multiPRS
**Major macrovascular**	**0.67** (0.64–0.69)	**0.72** (0.69–0.74)	0.026	**0.65** (0.62–0.67)	0.072
**Stroke**	**0.62** (0.58–0.67)	**0.68** (0.64–0.73)	0.509	**0.61** (0.57–0.66)	0.134
**Myocardial infarction**	**0.64** (0.61–0.68)	**0.64** (0.60–0.68)	0.373	**0.65** (0.61–0.69)	0.606
**Heart failure**	**0.66** (0.62–0.69)	**0.73** (0.70–0.77)	0.06	**0.61** (0.57–0.64)	0.005
**Cardiovascular death**	**0.71** (0.68–0.74)	**0.76** (0.72–0.79)	0.136	**0.65** (0.62–0.68)	0.002
**All cause death**	**0.68** (0.66–0.71)	**0.70** (0.67–0.73)	0.613	**0.64** (0.62–0.67)	0.005

C-statistics of different outcomes using multiPRS compared to clinical models (ADVANCE and Framingham clinical risk scores). 10-fold cross-validated AUCs and percentile-based confidence intervals (CI) were estimated from ROC curves and calculated from the predicted risks derived from the regression models. The multiPRS model is composed of the 10 wPRS, PC1, sex, age at diagnosis of T2D, and diabetes duration. The controls used did not have a specific outcome at any time during the study. The ADVANCE and Framingham clinical scores were calculated as described in the literature and tested for association with ADVANCE phenotypes using linear regression. **Abbreviation: CI:** Confidence intervals. P values are for differences between AUC of multiPRS and each of the clinical score.

ESM Table 7: Continuous net reclassification index adding the multiPRS to Framingham & ADVANCE clinical risk scores.

Outcomes	Reclassified predicted risk with multiPRS (%)			
	Framingham		ADVANCE	
	**Net reclassified**	Reclassified Decreased/Increased	**Net reclassified**	Reclassified Decreased/Increased
**Major macrovascular**	**36**	30/51	**24**	16/37
**Stroke**	**39**	29/63	**33**	24/61
**Myocardial infarction**	**45**	30/62	**41**	30/63
**Cardiovascular death**	**62**	48/73	**26**	19/48
**All cause death**	**49**	37/57	**24**	17/37

Reclassification of predicted risk (%) with the addition of multiPRS to Framingham and ADVANCE scores for macrovascular outcomes of T2D. Bootstrapping was performed with 1000 iterations.

Supplementary Figures

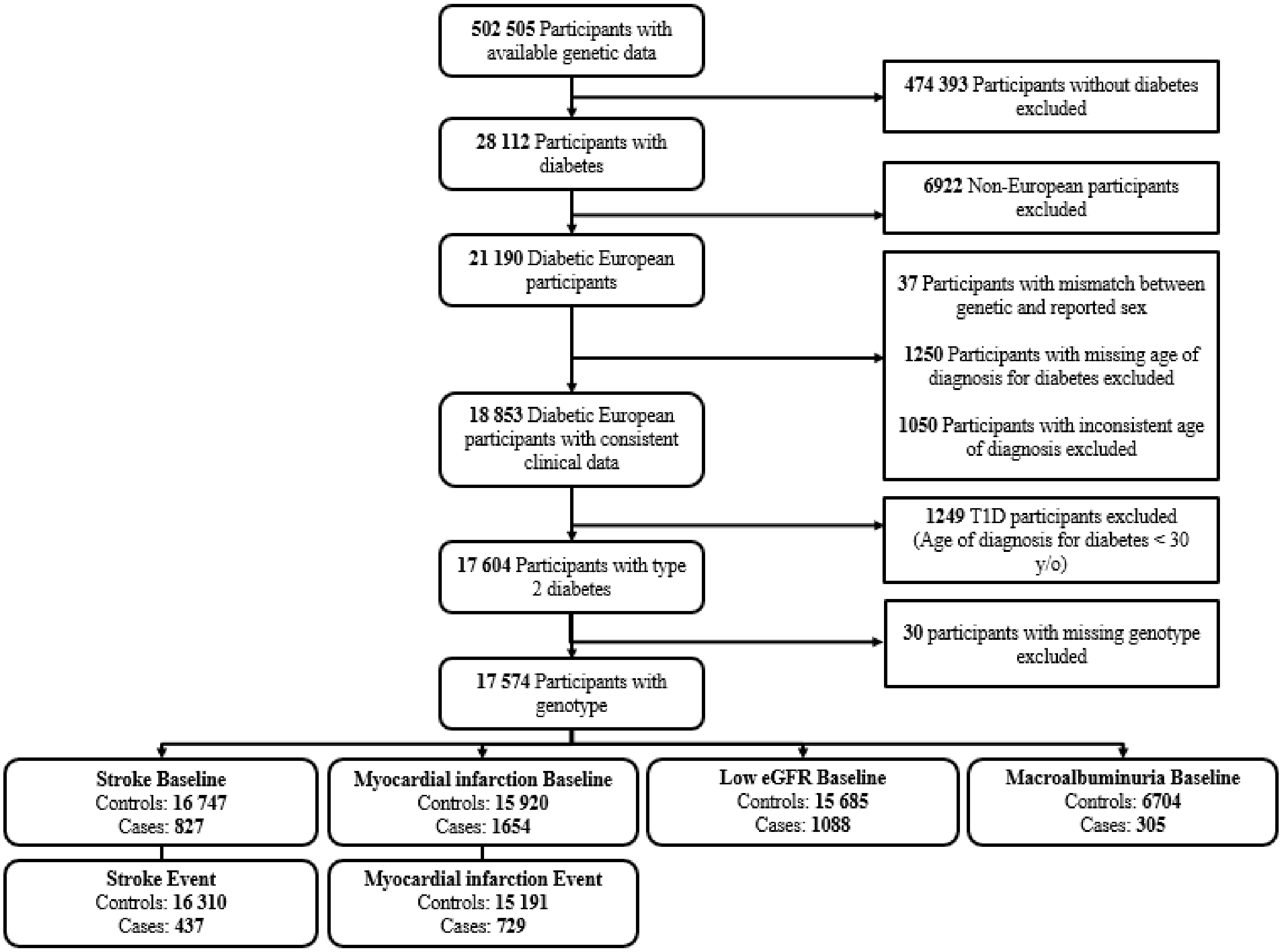

ESM Fig. 1: Flow of selection of individuals with T2D of UK Biobank (UKBB) and number of cases and controls for stroke, myocardial infarction, low eGFR and macroalbuminuria analyses.

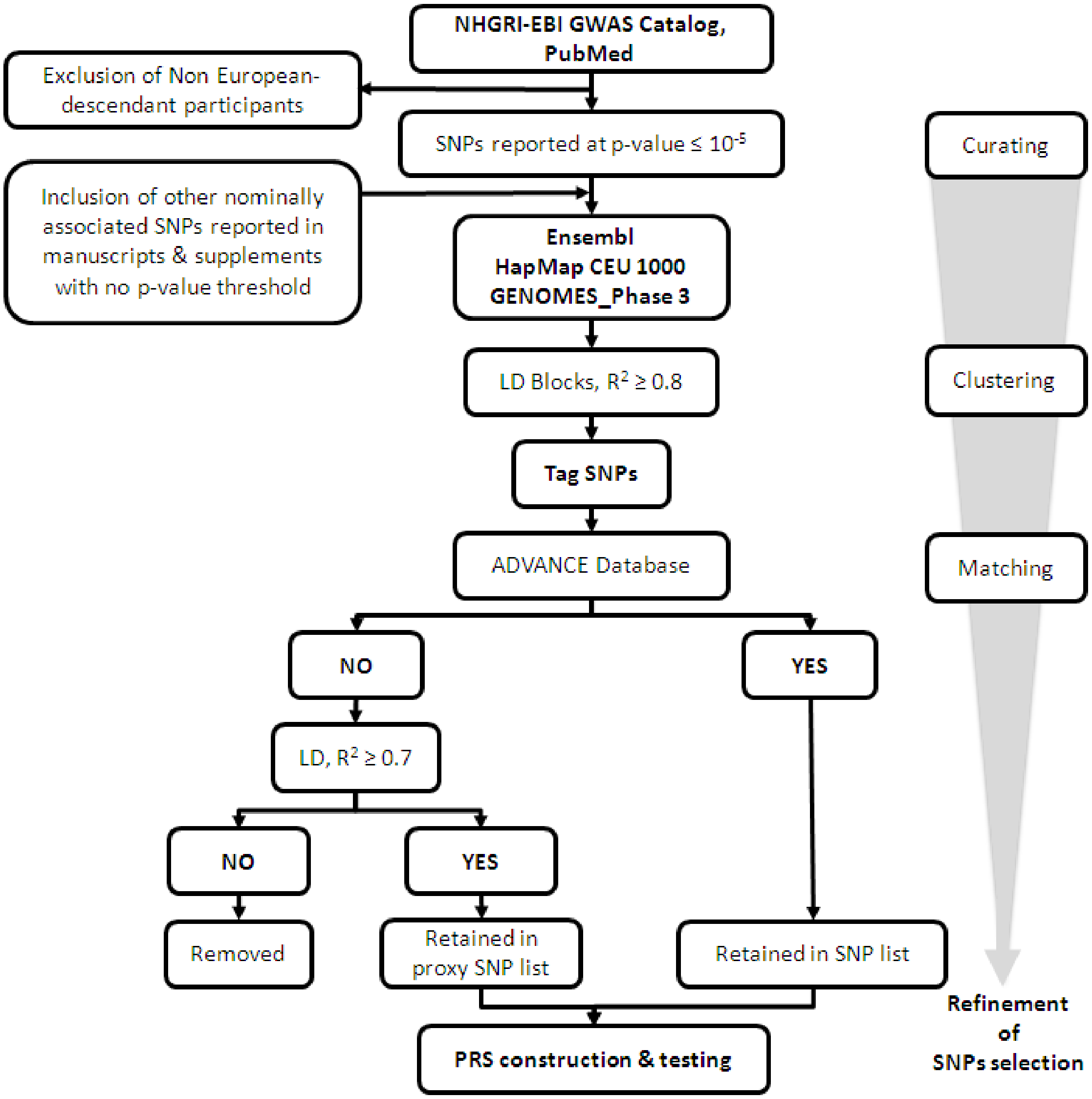

ESM Fig. 2: Stepwise approach for selection of SNPs included in the 10 wPRS.

SNP selection to construct the 10 wPRS integrates public-access resources including continuously updated database of GWAS results, NHGRI-EBI GWAS Catalog and PubMed. Curating consists of extraction of SNPs associated with one of the selected phenotypes at a given significance threshold from each GWAS and PubMed pertinent literature including only participants of European descent. Clustering: extracted SNPs are clustered according to patterns of linkage disequilibrium (LD) determined from HapMap CEU 1000 genomes reference population that matches the European population to yield a set of LD Blocks. Matching: Tag SNPs identified from LD blocks were used for matching with genotyped/ imputed SNPs. SNPs retained are usd for wPRS construction. **Abbreviation: NHGRI-EBI**

GWAS catalog: National human genome research institute–European bioinformatics institute catalog of published genome-wide association studies.

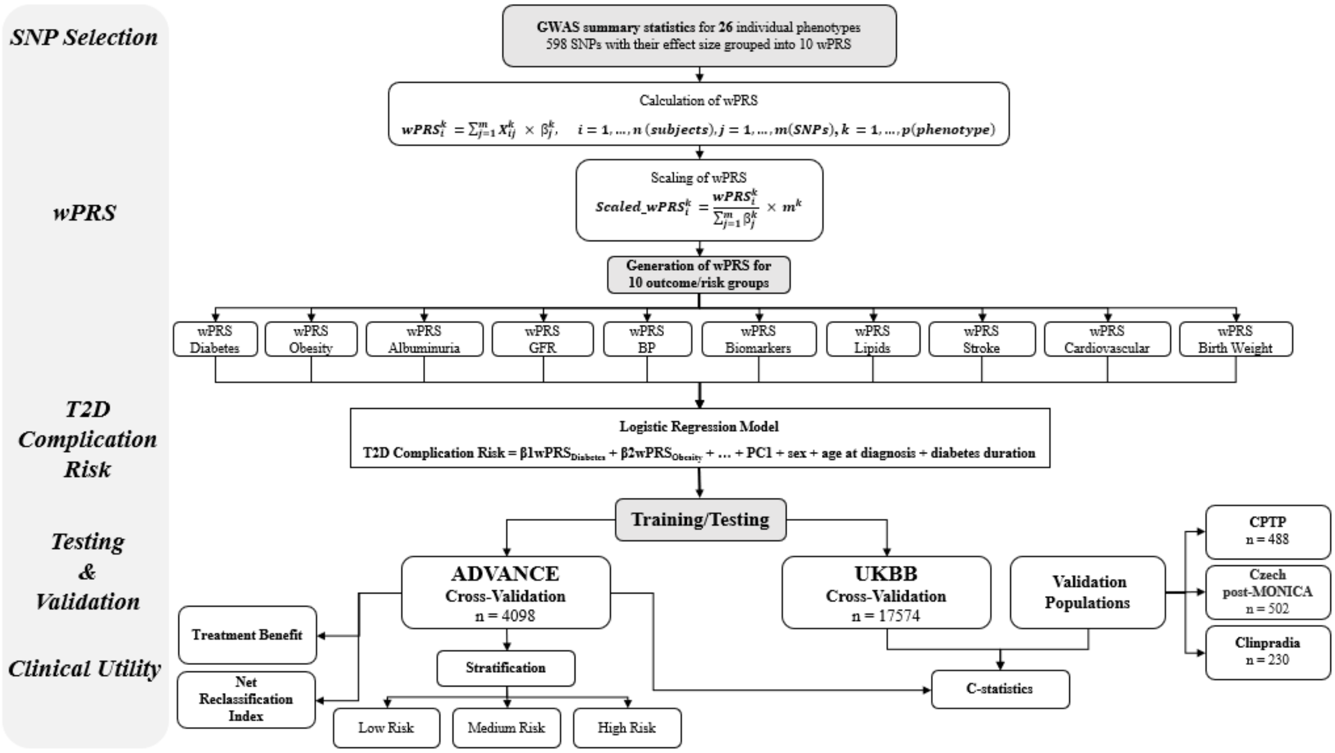

ESM Fig. 3: Prediction model construction and testing in ADVANCE and UKBB and validation in three independent cohorts of patients with T2D.

The prediction model was based on 10 wPRS. The logistic regression model that included the 10 wPRS, sex, PC1, age at onset of diabetes and its duration was tested in 4,098 participants of ADVANCE and 17,574 individuals with T2D of the UK Biobank after 10-fold cross-validation. The model was replicated in 488 individuals with T2D of CPTP, 230 individuals with T2D of Clinpradia and 502 individuals with pre-diabetes of Czech post-MONICA studies. Clinical utility was analyzed in ADVANCE trial.

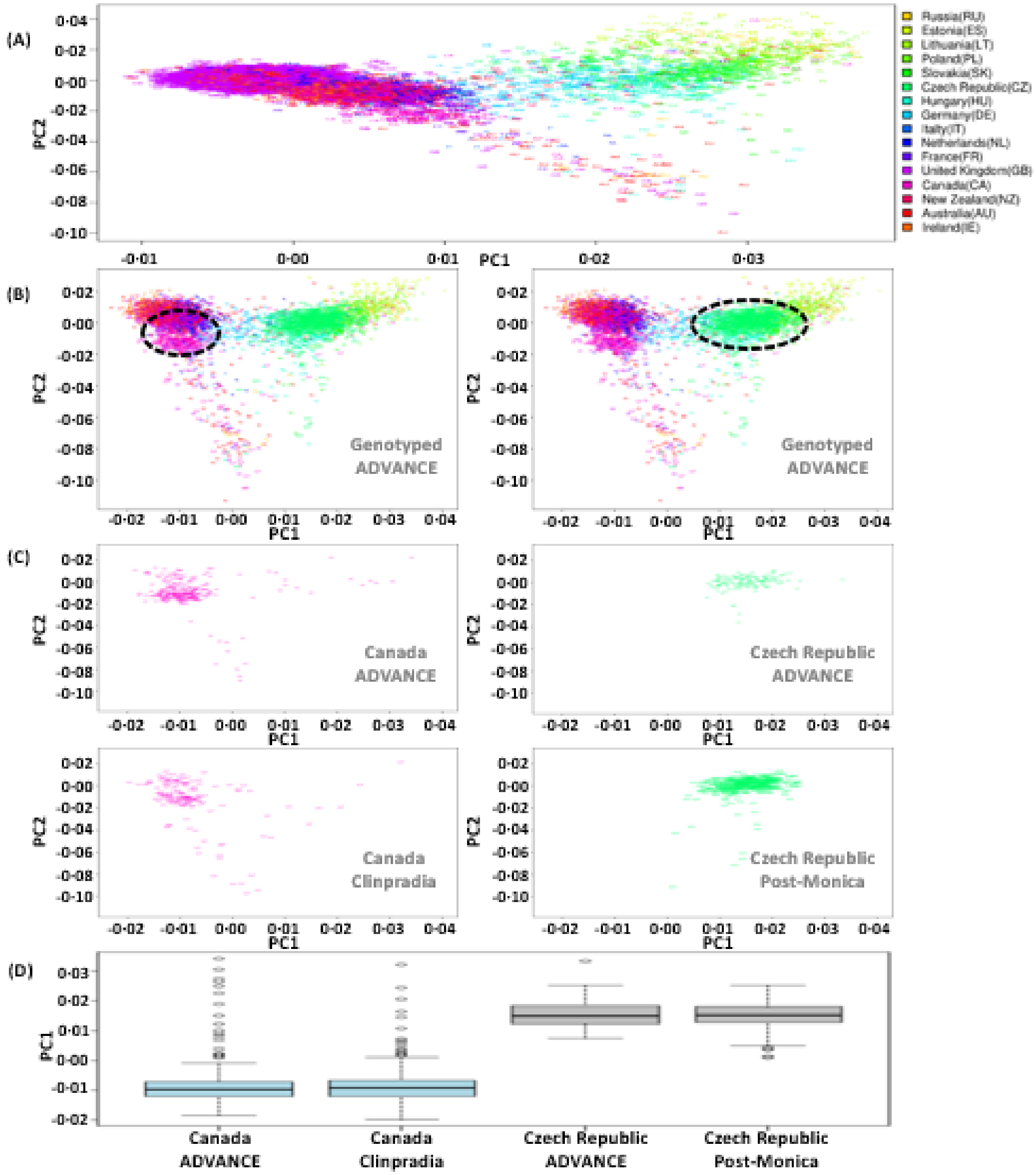

ESM Fig. 4: Population structure of the study cohorts using principal component analysis projection.

Principal components (PC) have been computed on ADVANCE individuals and all individuals from other cohorts were projected onto these principal components. **(A)** Projection of UKBB individuals on

ADVANCE PCs. **(B)** ADVANCE participants of Canadian (left) and Czech (right) origins are circled. **(C)** (Left) Distribution of participants from Clinpradia study performed in Canada and (Right) participants from Post-MONICA study performed in Czech Republic projected on ADVANCE PCs. **(D)** Box plots comparing mean PC1 values of participants in ADVANCE, Clinpradia and Post-Monica.

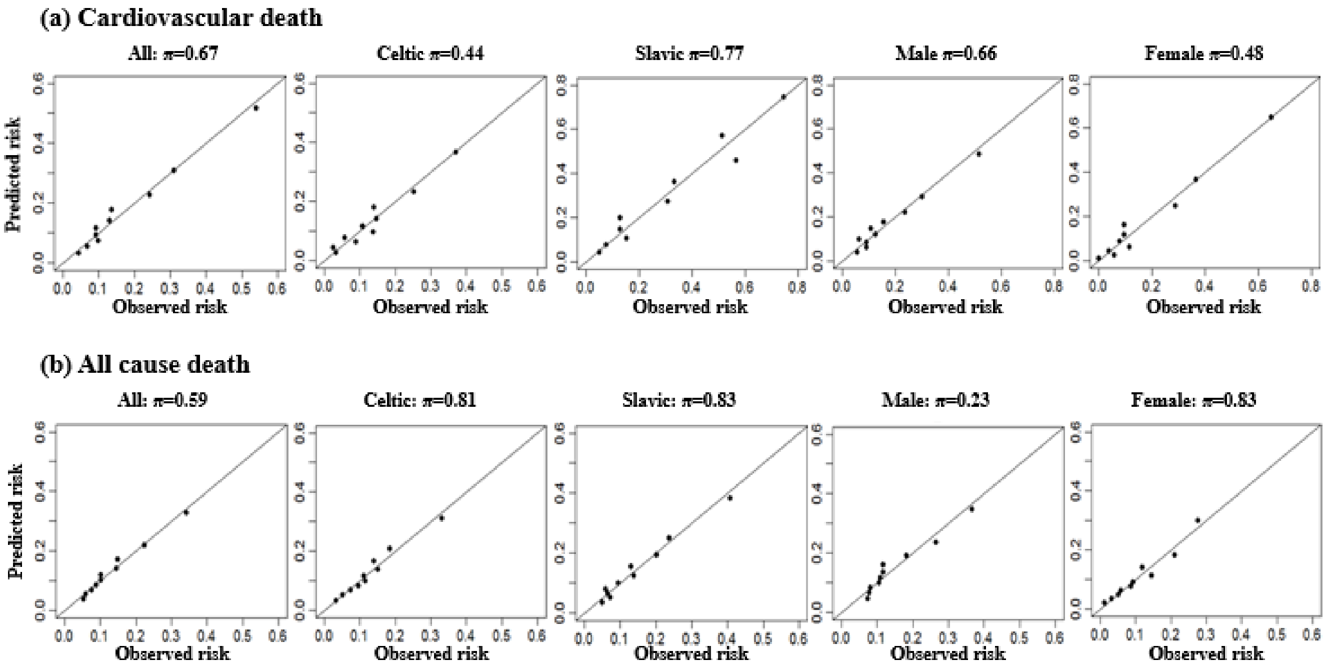

ESM Fig. 5: Hosmer-Lemeshow test.

Ethnic- and sex-specific calibration plots for the ADVANCE cardiovascular (a) and all cause death (b). The test is used to assess the goodness of fit for logistic regression models. The test assesses whether the observed event rates match expected event rates in subgroups of the model population. A value of π=1 means that the expected and observed event rates in subgroups are similar. The subgroups are defined by sex and ethnicity as defined by PC1. The null hypothesis is that the observed event rates match expected event rates. The points in the graphs represent the deciles of fitted risk values.

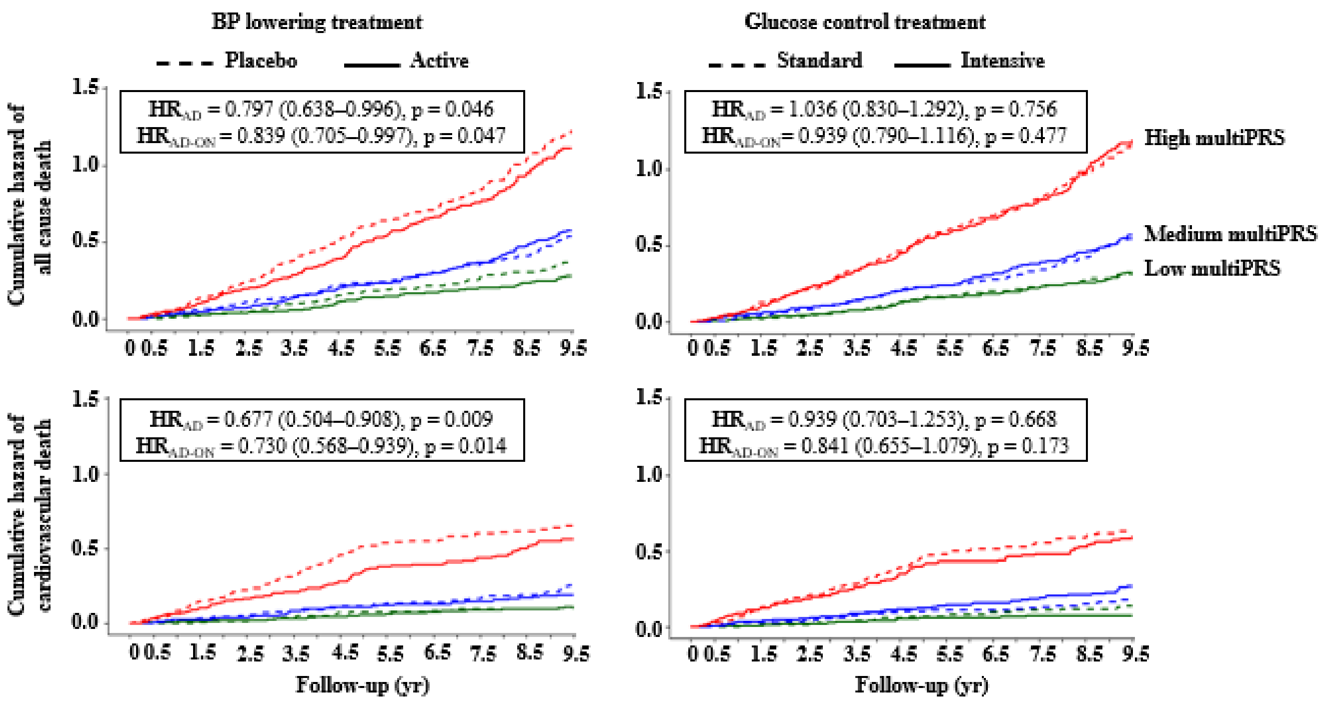

ESM Fig. 6: Cumulative hazard plots of all cause and cardiovascular death stratified by multiPRS thirds in standard and intensive blood pressure (left) and glucose (right) treatment arms.

Hazard ratio was analyzed by Cox proportional hazards regression analysis. Red: high multiPRS, blue: medium multiPRS, Green: low multiPRS. The control group includes normotensive subjects. HR indicated are for high multiPRS third. **Abbreviation: HR**: Hazard ratio, **AD**: ADVANCE trial, **AD-ON**: ADVANCE-ON follow-up.

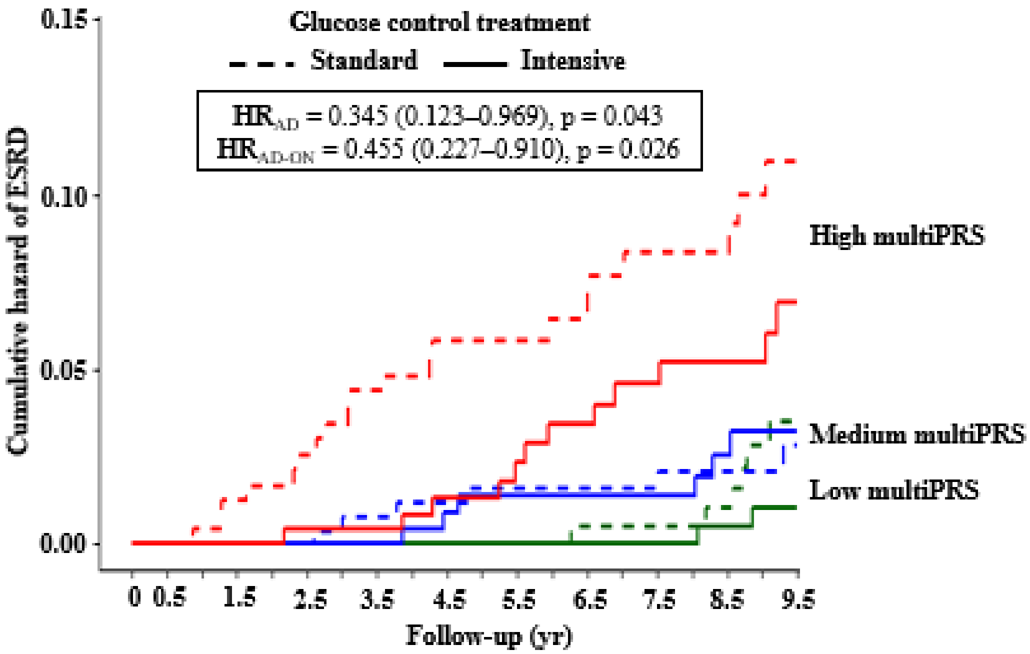

ESM Fig. 7: Cumulative hazard plots of ESRD stratified by multiPRS thirds in standard and intensive glucose treatment arm.

Hazard ratio was analyzed by Cox proportional hazards regression analysis. Red: high multiPRS, blue: medium multiPRS, Green: low multiPRS. The control group includes normotensive subjects. HR indicated are for high multiPRS third. **Abbreviation**: **HR**: Hazard ratio, **AD**: ADVANCE trial, **AD-ON**: ADVANCE-ON follow-up.

Supplementary References

1 Euesden J, Lewis CM, O'Reilly PF. PRSice: Polygenic Risk Score software. Bioinformatics 2015;31:1466–8.

2 ADVANCE Management committee. Study Rationale and Design of ADVANCE: Action in Diabetes and Vascular disease - preterax and diamicron MR controlled evaluation. Diabetologia 2001;44:1118–20.

3 Sudlow C, Gallacher J, Allen N, et al. UK Biobank: An Open Access Resource for Identifying the Causes of a Wide Range of Complex Diseases of Middle and Old Age. PLOS Med 2015;12:e1001779.

4 Patel A, MacMahon S, Chalmers J, Neal B, Woodward M, Billot. Effects of a fixed combination of perindopril and indapamide on macrovascular and microvascular outcomes in patients with type 2 diabetes mellitus (the ADVANCE trial): a randomised controlled trial. Lancet 2007;370:829–40.

5 Zoungas S, de Galan BE, Ninomiya T, et al. Combined Effects of Routine Blood Pressure Lowering and Intensive Glucose Control on Macrovascular and Microvascular Outcomes in Patients With Type 2 Diabetes: New results from the ADVANCE trial. Diabetes Care 2009;32:2068–74.

6 Zoungas S, Chalmers J, Neal B, et al. Follow-up of Blood-Pressure Lowering and Glucose Control in Type 2 Diabetes. N Engl J Med 2014;371:1392–406.

7 Krajcoviechova A, Tremblay J, Wohlfahrt P, et al. The Impact of Blood Pressure and Visceral Adiposity on the Association of Serum Uric Acid With Albuminuria in Adults Without Full Metabolic Syndrome. Am J Hypertens 2016;29:1335–42.

8 Awadalla P, Boileau C, Payette Y, et al. Cohort profile of the CARTaGENE study: Quebec's population-based biobank for public health and personalized genomics. Int J Epidemiol 2013;42:1285–99.

9 Ibrahim-Verbaas CA, Fornage M, Bis JC, et al. Predicting Stroke Through Genetic Risk Functions. Stroke 2014;45:403–12.

10 R Core team. R: A language and environment for statistical computing. R foundation for statistical computing 2018.

11 Gregory R. Warnes, Bolker B, Bonebakker L, et al. gplots: Various R programming tools for plotting data, R package version 3.0.1 2016.

12 Dalgaard P. Introductory statistics with R. 2nd ed. 2008.

13 Robin X, Turck N, Hainard A, et al. pROC: an open-source package for R and S+ to analyze and compare ROC curves. BMC Bioinformatics 2011;12:77.

14 DeLong ER, DeLong DM, Clarke-Pearson DL. Comparing the Areas under Two or More Correlated Receiver Operating Characteristic Curves: A Nonparametric Approach. Biometrics 1988;44:837.

15 Hamet P, Haloui M, Harvey F, et al. PROX1 gene CC genotype as a major determinant of early onset of type 2 diabetes in slavic study participants from Action in Diabetes and Vascular Disease. J Hypertens 2017;35:S24–32.

16 Raj A, Stephens M, Pritchard JK. fastSTRUCTURE: Variational Inference of Population Structure in

Large SNP Data Sets. Genetics 2014;197:573–89.

17 Purcell S, Neale B, Todd-Brown K, et al. PLINK: A Tool Set for Whole-Genome Association and Population-Based Linkage Analyses. Am J Hum Genet 2007;81:559–75.

18 Delaneau O, Marchini J, Zagury J-F. A linear complexity phasing method for thousands of genomes. Nat Methods 2012;9:179–81.

19 Howie BN, Donnelly P, Marchini J. A Flexible and Accurate Genotype Imputation Method for the Next Generation of Genome-Wide Association Studies. PLoS Genet 2009;5:e1000529.

20 https://mathgen.stats.ox.ac.uk/impute/1000GP_Phase3.html.

21 Cunningham F, Amode MR, Barrell D, et al. Ensembl 2015. Nucleic Acids Res 2015;43:D662–9.

22 Southam L, Panoutsopoulou K, Rayner NW, et al. The effect of genome-wide association scan quality control on imputation outcome for common variants. Eur J Hum Genet 2011;19:610–4.

23 Price AL, Patterson NJ, Plenge RM, Weinblatt ME, Shadick NA, Reich D. Principal components analysis corrects for stratification in genome-wide association studies. Nat Genet 2006;38:904–9.

24 O’Seaghdha CM, Yang Q, Wu H, Hwang S-J, Fox CS. Performance of a Genetic Risk Score for CKD Stage 3 in the General Population. Am J Kidney Dis 2012;59:19–24.

25 Voight BF, Scott LJ, Steinthorsdottir V, et al. Twelve type 2 diabetes susceptibility loci identified through large-scale association analysis. Nat Genet 2010;42:579–89.

26 Soranzo N, Sanna S, Wheeler E, et al. Common Variants at 10 Genomic Loci Influence Hemoglobin A1C Levels via Glycemic and Nonglycemic Pathways. Diabetes 2010;59:3229–39.

27 Dupuis J, Langenberg C, Prokopenko I, et al. New genetic loci implicated in fasting glucose homeostasis and their impact on type 2 diabetes risk. Nat Genet 2010;42:105–16.

28 Qi L, Cornelis MC, Kraft P, et al. Genetic variants at 2q24 are associated with susceptibility to type 2 diabetes. Hum Mol Genet 2010;19:2706–15.

29 Zeggini E, Scott LJ, Saxena R, et al. Meta-analysis of genome-wide association data and large-scale replication identifies additional susceptibility loci for type 2 diabetes. Nat Genet 2008;40:638–45.

30 Paré G, Chasman DI, Parker AN, et al. Novel Association of HK1 with Glycated Hemoglobin in a Non-Diabetic Population: A Genome-Wide Evaluation of 14,618 Participants in the Women’s Genome Health Study. PLoS Genet 2008;4:e1000312.

31 Kong A, Steinthorsdottir V, Masson G, et al. Parental origin of sequence variants associated with complex diseases. Nature 2009;462:868–74.

32 Shungin D, Winkler TW, Croteau-Chonka DC, et al. New genetic loci link adipose and insulin biology to body fat distribution. Nature 2015;518:187–96.

33 Speliotes EK, Willer CJ, Berndt SI, et al. Association analyses of 249,796 individuals reveal 18 new loci associated with body mass index. Nat Genet 2010;42:937–48.

34 Heid IM, Jackson AU, Randall JC, et al. Meta-analysis identifies 13 new loci associated with waist-hip ratio and reveals sexual dimorphism in the genetic basis of fat distribution. Nat Genet 2010;42:949–60.

35 Lindgren CM, Heid IM, Randall JC, et al. Genome-Wide Association Scan Meta-Analysis Identifies Three Loci Influencing Adiposity and Fat Distribution. PLoS Genet 2009;5:e1000508.

36 Teumer A, Tin A, Sorice R, et al. Genome-wide Association Studies Identify Genetic Loci Associated With Albuminuria in Diabetes. Diabetes 2016;65:803–17.

37 Pattaro C, Teumer A, Gorski M, et al. Genetic associations at 53 loci highlight cell types and biological pathways relevant for kidney function. Nat Commun 2016;7:10023.

38 Köttgen A, Pattaro C, Böger CA, et al. New loci associated with kidney function and chronic kidney disease. Nat Genet 2010;42:376–84.

39 Chambers JC, Zhang W, Lord GM, et al. Genetic loci influencing kidney function and chronic kidney disease. Nat Genet 2010;42:373–5.

40 Köttgen A, Glazer NL, Dehghan A, et al. Multiple loci associated with indices of renal function and chronic kidney disease. Nat Genet 2009;41:712–7.

41 Gudbjartsson DF, Holm H, Indridason OS, et al. Association of Variants at UMOD with Chronic Kidney Disease and Kidney Stones—Role of Age and Comorbid Diseases. PLoS Genet 2010;6:e1001039.

42 International Consortium for Blood Pressure Genome-Wide Association Studies, Georg B Ehret, Patricia B Munroe, Kenneth M Rice, Murielle Bochud, Andrew D Johnson, Daniel I Chasman, Albert V Smith, Martin D Tobin, Germaine C Verwoert, Shih-Jen Hwang, Vasyl Pihur, Peter Vollenweider, Paul F O'Reilly, Najaf Amin, Jennifer L Bragg-Gres TJ. Genetic variants in novel pathways influence blood pressure and cardiovascular disease risk. Nature 2011;478:103–9.

43 Wain L V., Vaez A, Jansen R, et al. Novel Blood Pressure Locus and Gene Discovery Using Genome-Wide Association Study and Expression Data Sets From Blood and the Kidney. Hypertension 2017;70:e4–19.

44 Warren HR, Evangelou E, Cabrera CP, et al. Genome-wide association analysis identifies novel blood pressure loci and offers biological insights into cardiovascular risk. Nat Genet 2017;49:403–15.

45 Wain L V, Verwoert GC, O'Reilly PF, et al. Genome-wide association study identifies six new loci influencing pulse pressure and mean arterial pressure. Nat Genet 2011;43:1005–11.

46 Dehghan A, Dupuis J, Barbalic M, et al. Meta-Analysis of Genome-Wide Association Studies in >80 000 Subjects Identifies Multiple Loci for C-Reactive Protein Levels. Circulation 2011;123:731–8.

47 Dehghan A, Yang Q, Peters A, et al. Association of Novel Genetic Loci With Circulating Fibrinogen Levels. Circ Cardiovasc Genet 2009;2:125–33.

48 Teslovich TM, Musunuru K, Smith A V., et al. Biological, clinical and population relevance of 95 loci for blood lipids. Nature 2010;466:707–13.

49 Dichgans M, Malik R, König IR, et al. Shared Genetic Susceptibility to Ischemic Stroke and Coronary Artery Disease. Stroke 2014;45:24–36.

50 Myocardial Infarction Genetics, Kathiresan S, Voight B, et al. Genome-wide association of early-onset myocardial infarction with single nucleotide polymorphisms and copy number variants. Nat Genet 2009;41:334–41.

51 Nikpay M, Goel A, Won H, et al. A comprehensive 1000 Genomes–based genome-wide association meta-analysis of coronary artery disease. Nat Genet 2015;47:1121–30.

52 Nelson CP, Goel A, Butterworth AS, et al. Association analyses based on false discovery rate implicate new loci for coronary artery disease. Nat Genet 2017;49:1385–91.

53 Pulit SL, McArdle PF, Wong Q, et al. Loci associated with ischaemic stroke and its subtypes (SiGN): a genome-wide association study. Lancet Neurol 2016;15:174–84.

54 Ellinor PT, Lunetta KL, Glazer NL, et al. Common variants in KCNN3 are associated with lone atrial fibrillation. Nat Genet 2010;42:240–4.

55 Cheng Y-C, Stanne TM, Giese A-K, et al. Genome-Wide Association Analysis of Young-Onset Stroke Identifies a Locus on Chromosome 10q25 Near HABP2. Stroke 2016;47:307–16.

56 Qi L, Qi Q, Prudente S, et al. Association Between a Genetic Variant Related to Glutamic Acid Metabolism and Coronary Heart Disease in Individuals With Type 2 Diabetes. J Am Med Assoc 2013;310:821–828.

57 Traylor M, Farrall M, Holliday EG, et al. Genetic risk factors for ischaemic stroke and its subtypes (the METASTROKE Collaboration): a meta-analysis of genome-wide association studies. Lancet Neurol 2012;11:951–62.

58 Benjamin EJ, Rice KM, Arking DE, et al. Variants in ZFHX3 are associated with atrial fibrillation in individuals of European ancestry. Nat Genet 2009;41:879–81.

59 Bellenguez C, Bevan S, Gschwendtner A, et al. Genome-wide association study identifies a variant in HDAC9 associated with large vessel ischemic stroke. Nat Genet 2012;44:328–33.

60 Dehghan A, Bis JC, White CC, et al. Genome-Wide Association Study for Incident Myocardial Infarction and Coronary Heart Disease in Prospective Cohort Studies: The CHARGE Consortium. PLoS One 2016;11:e0144997.

61 Holliday EG, Maguire JM, Evans T-J, et al. Common variants at 6p21.1 are associated with large artery atherosclerotic stroke. Nat Genet 2012;44:1147–51.

62 Matarín M, Brown WM, Scholz S, et al. A genome-wide genotyping study in patients with ischaemic stroke: initial analysis and data release. Lancet Neurol 2007;6:414–20.

63 Samani NJ, Erdmann J, Hall AS, et al. Genomewide Association Analysis of Coronary Artery Disease. N Engl J Med 2007;357:443–53.

64 Clarke R, Peden JF, Hopewell JC, et al. Genetic Variants Associated with Lp(a) Lipoprotein Level and Coronary Disease. N Engl J Med 2009;361:2518–28.

65 Foroud T, Lai D, Koller D, et al. Genome-Wide Association Study of Intracranial Aneurysm Identifies a New Association on Chromosome 7. Stroke 2014;45:3194–9.

66 Foroud T, Koller DL, Lai D, et al. Genome-Wide Association Study of Intracranial Aneurysms Confirms Role of Anril and SOX17 in Disease Risk. Stroke 2012;43:2846–52.

67 van Setten J, Isgum I, Smolonska J, et al. Genome-wide association study of coronary and aortic calcification implicates risk loci for coronary artery disease and myocardial infarction. Atherosclerosis 2013;228:400–5.

68 O'Donnell CJ, Kavousi M, Smith A V., et al. Genome-Wide Association Study for Coronary Artery Calcification With Follow-Up in Myocardial Infarction. Circulation 2011;124:2855–64.

69 Reilly MP, Li M, He J, et al. Identification of ADAMTS7 as a novel locus for coronary atherosclerosis and association of ABO with myocardial infarction in the presence of coronary atherosclerosis: two genome-wide association studies. Lancet 2011;377:383–92.

70 Ikram MA, Seshadri S, Bis JC, et al. Genomewide Association Studies of Stroke. N Engl J Med 2009;360:1718–28.

71 Horikoshi M, Beaumont RN, Day FR, et al. Genome-wide associations for birth weight and correlations with adult disease. Nature 2016;538:248–52.
